# Identifying acute illness phenotypes via deep temporal interpolation and clustering network on physiologic signatures

**DOI:** 10.1038/s41598-024-59047-x

**Published:** 2024-04-10

**Authors:** Yuanfang Ren, Yanjun Li, Tyler J. Loftus, Jeremy Balch, Kenneth L. Abbott, Matthew M. Ruppert, Ziyuan Guan, Benjamin Shickel, Parisa Rashidi, Tezcan Ozrazgat-Baslanti, Azra Bihorac

**Affiliations:** 1 Intelligent Clinical Care Center, University of Florida, Gainesville, FL USA; 2 Division of Nephrology, Hypertension, and Renal Transplantation, Department of Medicine, University of Florida, PO Box 100224, Gainesville, FL 32610-0254 USA; 3 Department of Medicinal Chemistry, College of Pharmacy, University of Florida, Gainesville, FL USA; 4 Center for Natural Products, Drug Discovery and Development, University of Florida, Gainesville, FL USA; 5 Department of Surgery, University of Florida, Gainesville, FL USA; 6 J. Crayton Pruitt Family Department of Biomedical Engineering, University of Florida, Gainesville, FL USA

**Keywords:** Health care, Public health

## Abstract

Using clustering analysis for early vital signs, unique patient phenotypes with distinct pathophysiological signatures and clinical outcomes may be revealed and support early clinical decision-making. Phenotyping using early vital signs has proven challenging, as vital signs are typically sampled sporadically. We proposed a novel, deep temporal interpolation and clustering network to simultaneously extract latent representations from irregularly sampled vital signs and derive phenotypes. Four distinct clusters were identified. Phenotype A (18%) had the greatest prevalence of comorbid disease with increased prevalence of prolonged respiratory insufficiency, acute kidney injury, sepsis, and long-term (3-year) mortality. Phenotypes B (33%) and C (31%) had a diffuse pattern of mild organ dysfunction. Phenotype B’s favorable short-term clinical outcomes were tempered by the second highest rate of long-term mortality. Phenotype C had favorable clinical outcomes. Phenotype D (17%) exhibited early and persistent hypotension, high incidence of early surgery, and substantial biomarker incidence of inflammation. Despite early and severe illness, phenotype D had the second lowest long-term mortality. After comparing the sequential organ failure assessment scores, the clustering results did not simply provide a recapitulation of previous acuity assessments. This tool may impact triage decisions and have significant implications for clinical decision-support under time constraints and uncertainty.

## Introduction

Every year in the United States, there are more than 36 million hospital admissions, with approximately seven hundred thousand in-hospital deaths, nearly one-fourth of which are potentially preventable^1–3^. In the early stages after hospital admission, significant sources of preventable harm come from the misdiagnosis of high-risk patients and their under-triage to general hospital wards^4,5^. In this crucial period, clinicians are required to make a series of decisions on limited data that can significantly influence the patient's clinical trajectory^1,6,7^. This series of decisions entails analysis of a variety of data representing essential physiologic processes^6–8^. For example, vital sign values and trends may indicate whether a patient requires intensive monitoring in an intensive care unit (ICU) or if they can be safely transferred to a hospital’s general ward. The trajectories of early vital signs may be useful for identifying distinct physiological signatures that are linked to specific patient phenotypes and clinical outcomes.

Clustering analyses of vital signs and other clinical variables have shown promise for helping clinicians identify novel clinical phenotypes for sepsis and acute respiratory distress syndrome^8–11^. However, these phenotypes have not been evaluated with large, heterogeneous cohorts that include all hospitalized patients. In addition, broader phenotyping based on vital signs has proven more challenging, in part because sampling occurs at irregular intervals, thus complicating the application of conventional time-series analyses and machine learning clustering techniques^12–15^. In the past decade, however, deep learning has garnered significant achievements in the healthcare domain to facilitate the clinical decision-making process with its superior capability to detect the intricate patterns inherent in raw clinical data and to approximate highly complex functions^16–18^. Although several advanced deep learning algorithms have been developed to manage the irregularly-sampled time-series data^19–26^, there remains a dearth of work specifically focused on the clinical phenotype identification, particularly using the early stage vital sign data.

To fill this gap and address the patient stratification challenge, our study presents a novel deep temporal interpolation and clustering (dTIC) network. This innovative tool is designed to extract latent representations from sparse and irregularly sampled time-series vital sign data, and concurrently stratify patients into distinct phenotypes. The dTIC network exhibits considerable potential to effectively facilitate clinical decision-making, offering a promising solution to existing limitations in patient phenotype identification during the critical initial hours of hospital admission.

## Related work

In this section, we review existing clustering approaches for patient phenotyping based on multivariate time-series data. These approaches fall into two distinct categories: conventional feature engineering-based machine learning clustering methods and data-driven deep learning based clustering methods.

Current machine learning clustering approaches for handling irregularly sampled multivariate time-series data typically involve resampling the data into evenly spaced time-series data and impute the missing values using mean values from training cohort or other straightforward methods such as carrying forward the last available measurement. Based on the filled data points, Bhavani et al. applied the group based trajectory model (GBTM) algorithm to time-series vital sign data and identified four sepsis subphenotypes^12,13^. GBTM is an application of mixture modeling and calculates the coefficients for the polynomial functions, which describe time-based trajectories of vital signs in each group. The vital sign data were split into eight 1-h block of time and mean measurement in each block was used for clustering analysis. Imputation for missing values is not required for GBTM algorithm. Ren et al. applied the consensus k-means clustering algorithm to six time-series vital sign data and identified four acute illness phenotypes^14^. Before the clustering analysis, resampling was performed along with data imputation using both forward and backward propagation. Xu et al. first used dynamic time wrapping to compute similarities of Sequential Organ Failure Assessment (SOFA) trajectory and then applied hierarchical agglomerative clustering method to identify sepsis subphenotypes^15^. The SOFA trajectory data was evenly spaced and missing SOFA score was imputed using both forward and backward propagation.

However, resampling the irregularly sampled time-series data into evenly spaced data is challenging to comprehensively capture the temporal pattern. Thus, some efforts have been investigated to develop data-driven deep learning based clustering approaches to capture the dynamic nature of real-world time-series physiological data without resampling and imputation. For example, Qin et al. proposed the T-Phenotype approach to discover phenotypes of predictive temporal patterns from labeled time-series data^22^. T-Phenotype consists of a Laplace encoder network transforming the time-series into a fixed-length latent embedding, a predictor network estimating the conditional label distribution and a graph constrained K-means clustering which generates the clustering using a similarity graph constructed from the pair-wise connectivity test in latent space. T-Phenotype was applied to identify phenotypes for Alzheimer’s disease and ICU admission. Chen et al. proposed a deep generative approach SubLign for disease phenotyping^23^. SubLign focuses on learning latent representations that accurately reflect disease progression by correcting for temporal misalignments in observational data. Additionally, the approach explores the conditions necessary for the accurate identification of subtypes and alignment values in the context of disease data analysis. Experiments on clinical datasets of heart failure and Parkinson’s disease demonstrated that SubLign can successfully correct for the error from interval censoring and recover clinical subtypes. Ghaderi et al. proposed a novel self-supervised learning based clustering approach SLAC-Time for multivariate time-series data with missing values^24–26^. SLAC-Time is transformer based approach and consists of two alternating modules, a clustering model learning feature representations and extracting pseudo-labels, and a prediction model that predicts the extracted pseudo-labels. Application to traumatic brain injury phenotyping identified three distinct physiological states and discovered specific clinical events can affect the physiological states.

Despite these promising studies for clustering real-world time-series physiological data, there still remains a dearth of work specifically focused on the clinical phenotype identification on large, heterogeneous cohorts that include all hospitalized patients, particularly using the early stages vital sign data. Our proposed dTIC network aims to fill this gap, which not only captures the clustering-friendly dynamic patterns of physiological data, but also identifies distinct acute illness phenotypes.

## Methods

### Data source and participants

By using electronic health records (EHR) of 75,762 hospital admissions of 43,598 unique patients that represent adults (18 years or older) of all demographics, we created a longitudinal dataset of adult patients in the University of Florida Health’s academic hospital, a 1111-bed tertiary care facility with 241 intensive care beds, who remained admitted for six hours or longer (including emergency department admission) between June 1, 2014, and April 1, 2016. We excluded patients without sufficient vital sign measurements within six hours of hospital admission—that is, when at least two of the six total vital sign measurements (heart rate, systolic blood pressure, diastolic blood pressure, respiratory rate, oxygen saturation, and temperature) were completely missing (eFig. 1). This study was approved by the University of Florida institutional review board as exempt with waiver of informed consent (IRB201901123). All methods were performed in accordance with relevant regulations and guidelines.

### Study design

To mitigate any consequences of dataset drift because of adjustments in clinical practice or patient population, we adhered to the guidelines of the Type 2b analysis category^27^ under the Transparent Reporting of a multivariate prediction model for Individual Prognosis or Diagnosis (TRIPOD) in order to split the dataset chronologically into three categories—training (patients admitted from June 1, 2014, to May 31, 2015, n = 41,502), validation (patients admitted from June 1, 2015, to October 31, 2015, n = 17,415), and testing (patients admitted from November 1, 2015, to April 1, 2016, n = 16,845)—which followed a previous paper setting^14^. Using the training cohort, we identified acute illness phenotypes by applying unsupervised machine learning clustering to chronologically ordered measurements of patient vital signs from the first six hours of hospital admission. We utilized the validation cohort to select the hyper-parameters of our dTIC model. Within the testing cohort, we assessed phenotype reproducibility by predicting phenotypes and analyzing phenotype frequency distributions and clinical outcomes.

### Identifying acute illness phenotypes via early physiologic signatures

We removed outliers from raw time-series vital signs and explored distributions, missingness, and correlation (eTable 1 and eFig. 2). When a time-series variable was entirely absent from a patient's record, we imputed it using the mean value derived from the training cohort, assigning a timestamp corresponding to the patient’s hospital admission time.

We proposed a dTIC network to extract representations from sparse and irregularly sampled time-series data such as vital signs and subsequently derive acute illness phenotypes. We begin describing the dTIC network by presenting notation, followed by the model architecture and learning criteria. We denote the dataset containing $N$ samples with $D=\{({{\varvec{t}}}^{i}, {{\varvec{X}}}^{i})|i=1,\ldots ,N\}$, where each sample $\left({{\varvec{t}}}^{i}, {{\varvec{X}}}^{i}\right)$ consists of an irregularly sampled multivariate time-series. Each sample can have observations at different times with various number of variables, as well as different total number of observations. We denote the number of observations for sample $\left({{\varvec{t}}}^{i}, {{\varvec{X}}}^{i}\right)$ with ${L}_{i}$. Thus, we represent sample $\left({{\varvec{t}}}^{i}, {{\varvec{X}}}^{i}\right)$ as follows: ${{\varvec{t}}}^{i}=[{t}_{1}^{i}, \ldots ,{t}_{{L}_{i}}^{i}] \in {\mathcal{R}}^{{L}_{i}}$ is a vector of ${L}_{i}$ time points at which observations are defined and ${{\varvec{X}}}^{i}=[ {{\varvec{x}}}_{1}^{i}, \ldots ,{{\varvec{x}}}_{{L}_{i}}^{i}{]}^{T}\in {\mathcal{R}}^{{L}_{i} \times d}$ is a matrix representing a time-series with $d$ variables of length ${L}_{i}$ where ${{\varvec{x}}}_{j }^{i}\in {\mathcal{R}}^{d}$ represents the *j*-th observations of all variables. Notice that the $d$-dimensional observations may have the missing values (i.e., at a time point, temperature was not observed), and we fill the missing values with default value 0. We introduce a mask matrix ${M}^{i}= [ {{\varvec{m}}}_{1}^{i}, \ldots ,{{\varvec{m}}}_{{L}_{i}}^{i}{]}^{T}$ where ${{\varvec{m}}}_{j}^{i} \in \{0, 1 {\}}^{d}$ denotes which variables are missing at *j*-th time point.

**Figure 1:**
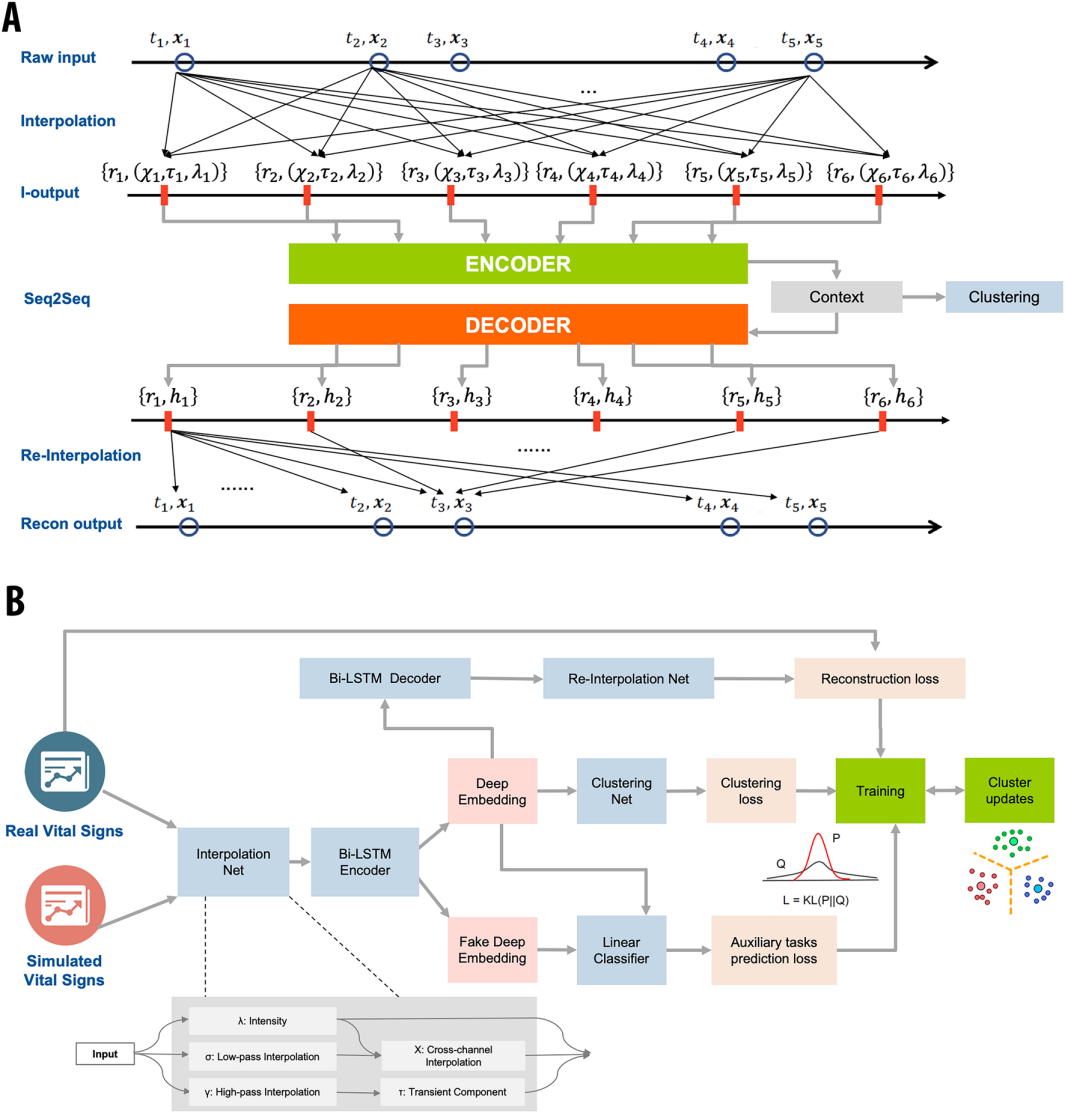
Schematic representation of deep temporal interpolation and clustering network architecture. (**A**) The detailed architecture of deep interpolation network, specifically tailored for handling sparse and irregularly sampled time-series data. ${t}_{i}$ is the time point of raw time-series data, and ${x}_{i}$ is the corresponding observed value. ${r}_{i}$ is the reference time point of interpolated time-series data, and $({\chi }_{i}, {\tau }_{i}, {\lambda }_{i})$ represents the three different channel outputs from interpolation network capturing the smooth trends, transient and local observation intensity respectively. ${h}_{i}$ is the hidden status from decoder network at reference time point ${r}_{i}$. (**B**) The full architecture of deep temporal interpolation and clustering network, designed to concurrently extract the feature representation and determine cluster assignments.

The dTIC network consists of four components: an interpolation model, a Seq2Seq model, a re-interpolation model, and a clustering model (Fig. 1 and eFig. 3). We detail the components of the dTIC as follows.

#### Interpolation model

It is common that the time-series vital sign data in electronic health records to be both sparse and irregularly sampled, which means large and irregular intervals widely exist between the data observation time points. Such sparsity and irregularity pose a significant challenge for machine learning and deep learning techniques to analyze the crucial vital sign data for improving the human health outcome. To deal with this challenge, we adopt the network proposed by Shukla and Marlin^20^ first to interpolate the raw time-series data to a regularly sampled meta-representation with pre-defined reference time points.

In our study, we utilize six vital signs multivariate time-series data. For simplicity, here we take one variable out of six as an example and we omit the superscript that denotes the sample index. For one sample, the raw time-series data is denoted as $e=\{({t}_{i}, {x}_{i})|i=1,\ldots ,L\}$, where $L$ represents the total number of observations, ${t}_{i}$ is the time point, and ${x}_{i}$ is the corresponding observed value. The time intervals between adjacent observation time points vary a lot. The interpolation model can map irregular $e$ value to the regular time-series data which is defined at the $T$ reference time points $r=\left[{r}_{1};\ldots ;{r}_{T}\right]$ with evenly spaced interval (see Fig. 1a).

The interpolation model consists of two layers, where the first layer separately performs the interpolation for each variable, and the second layer aggregates the information across all the studied variables. The model generates three different channel groups at each reference time point, which respectively represents smooth trends $\chi$, short time-scale transients $\tau$, and local observation frequencies $\lambda$. The interpolation model enables the single observation data point to be considered by all the reference time points and allows for the information to be shared across multiple variables. For more detailed interpolation mathematic denotation, the reader is referred to Shukla and Marlin^20^.

#### Seq2Seq model

With the interpolated time-series data as the input, we develop a Seq2Seq model to learn its low-dimensional representation, which can embed the contextual information over the full timeline (Fig. 1a). Seq2Seq model is a method of the encoder-decoder framework that maps an input of sequence to an output of sequence, and it is broadly used in machine translation, text summarization, conversational modeling, and some other tasks. With a single layer GRU network^28^ as the encoder, the input sequence is encoded to a fixed-length contextual vector ${h}_{T}$, which is the hidden state of the last time step. The hidden state of GRU updating mechanism, illustrated in the following equation, ensures that every internal hidden node state will be calculated by the previous state ${h}_{i-1}$ and current time step input $({\chi }_{i}, {\tau }_{i}, {\lambda }_{i})$.

$${h}_{i}= {GRU}_{Enc}(\left({\chi }_{i}, {\tau }_{i}, {\lambda }_{i}\right), {h}_{i-1})$$

A single-layer GRU network is also used for a decoder. At each time step, the decoder updates its current hidden state ${s}_{t}$ with the concatenated features incorporating the previous decoded output ${o}_{t-1}$ and global context vector ${h}_{T}$ as the input:

$${s}_{t}= {GRU}_{Dec}(\left[{o}_{t-1};{h}_{T}\right],{s}_{t-1})$$

#### Re-interpolation model

To unsupervised learn the useful representation, a common strategy is to build an auto-encoder learning framework by reconstructing the input itself from the extracted bottleneck representation. Therefore, on top of the Seq2Seq model, we develop a re-interpolation network to map the output with the evenly spaced intervals to the raw irregular time points (Fig. 1a). Similar to the interpolation model, the transformation is also based on a radial basis function network. Our re-interpolation model allows the embedded values at every reference time point to make a continuous contribution to reconstructed values at all the raw time points, but the contribution weight is exponentially decayed in terms of the distance between the referenced time point ${r}_{i}$ and target time point ${t}_{j}$:

$$w\left({r}_{i}, {t}_{j}, \theta \right)={\text{exp}}(-\theta ({r}_{i}, {t}_{j}{)}^{2})$$

where $\theta$ is learnable network parameters.

#### Clustering model

We stack a clustering network on top of the aforementioned feature extraction model to perform concurrent representation learning and clustering. The goal is to enhance the alignment of feature representations and cluster assignments^29^. This integrated approach demonstrates significant potential in learning clustering-friendly representations by which objects can be effectively grouped. The clustering network takes the low-dimensional features ${h}_{T}$ and initial cluster centroids as input. The initial cluster assignments are obtained using the contextual representation ${h}_{T}$ and k-means clustering^30^.

The clustering model simultaneously learns feature representations and cluster assignments using deep neural networks. It alternates between two steps: (1) computing a soft assignment between the embedded points and the cluster centroids by measuring the similarity between them; (2) refining the cluster centroids by minimizing the KL divergence between the soft assignment and the auxiliary target distribution. For more detailed description of clustering model, the reader is referred to Xie et al.^29^.

dTIC learns the feature representations and cluster assignments via a two-pass manner. In the first pass, dTIC learns the feature representations, determines the optimal number of clusters and thereby generates the initial cluster assignments. In the second pass, dTIC simultaneously learns feature representations and cluster assignments to refine the results.

Figure 1a provides the schematic representation of the dTIC architecture for feature learning process in the first learning pass. We first interpolate the raw time-series vital sign data to a regularly sampled meta-representation with pre-defined reference time points via the interpolation model. Then we feed the interpolated time-series data into a Seq2Seq model for feature embedding and extracting a unified context vector lying in the low-dimensional feature space by the encoder. The context vector contains the global time-series information and is further used by other downstream tasks (e.g., clustering, classification). The decoder in the Seq2Seq model learns from the context vector and outputs the time-series data with the same length of the Seq2Seq model’s input. Then, we deploy a radial basis function network-based model to re-interpolate the fixed-length output to the raw irregular time points for reconstructing the raw vital signs data at corresponding time points. To foster a more comprehensive representation of time-series data, we employ two auxiliary prediction tasks: (1) predicting the minimum values of systolic and diastolic blood pressure as well as oxygen saturation, along with the maximum values of heart rate, respiratory rate, and temperature within the seventh hour; and (2) predicting whether the learned representation originates from actual time-series data, by feeding both real and synthetic time-series data into the model. We generate the synthetic time-series data by randomly replacing values of real data at 50% time points. It worth noting that synthetic time series data is only used for classification and is not counted in the optimization of reconstruction loss and clustering loss. The full feature extraction model is end-to-end trained by minimizing the reconstruction loss measured with mean square error (real data only) and auxiliary prediction task loss measured with mean square error for task (1) and binary cross-entropy for task (2):

$${\mathcal{L}}_{reconstruction }= \frac{1}{\sum_{i=1}^{N}({{\varvec{M}}}^{i})}{\sum }_{i=1}^{N}{\Vert {{\varvec{M}}}^{i}\odot {{\varvec{X}}}^{i}-{{{\varvec{M}}}^{i}\odot \widehat{{\varvec{X}}}}^{i}\Vert }^{2}$$

$${\mathcal{L}}_{auxiliary\_1 }= \frac{1}{\sum_{i=1}^{N}({{\varvec{m}}}_{{t}{\prime}}^{i})}{\sum }_{i=1}^{N}{\Vert {{\varvec{m}}}_{{t}{\prime}}^{i}\odot {{\varvec{x}}}_{{t}{\prime}}^{i}-{{\varvec{m}}}_{{t}{\prime}}^{i}\odot {\widehat{{\varvec{x}}}}_{{t}{\prime}}^{i}\Vert }^{2}$$

$${\mathcal{L}}_{auxiliary\_2 }= -\frac{1}{2N}{\sum }_{j=1}^{2N}[{y}^{j}log\left({\widehat{y}}^{j}\right)+(1-{y}^{j})log\left(1-{\widehat{y}}^{j}\right)]$$

$$\mathcal{L}={\mathcal{L}}_{reconstruction }+ \alpha {\mathcal{L}}_{auxiliary\_1 }+\beta {\mathcal{L}}_{auxiliary\_2}$$

where ${\widehat{{\varvec{X}}}}^{i}$ is the reconstructed time-series data, ${{\varvec{m}}}_{{t}{\prime}}^{i}$ is the mask indicator at seventh hour, ${{\varvec{x}}}_{{t}{\prime}}^{i}$ is the vector of minimum/maximum vital sign values at seventh hour, ${\widehat{{\varvec{x}}}}_{{t}{\prime}}^{i}$ is the predicted vital sign value, ${y}^{j}$ is the indicator of whether the input time-series data is real and ${\widehat{y}}^{j}$ is the predicted indicator value. The extracted feature representation (context vector) will be used to determine the optimal number of clusters and obtain the initial cluster assignment.

Figure 1b provides the overall schematic representation of the dTIC architecture for feature representation learning and cluster assignment process. In addition to the feature extraction model, a clustering network is added in the second learning pass. The full dTIC model is end-to-end trained by minimizing the reconstruction loss, auxiliary prediction task loss and clustering loss. The clustering loss is measured with Kullback–Leibler (KL) divergence between an embedded distribution and a target data distribution.

$${\mathcal{L}}_{clustering }=KL(P|\left|Q\right)= \sum_{i}\sum_{j}{p}_{ij}log\frac{{p}_{ij}}{{q}_{ij}}$$

$$\mathcal{L}={\mathcal{L}}_{reconstruction }+ \alpha {\mathcal{L}}_{auxiliary\_1 }+\beta {\mathcal{L}}_{auxiliary\_2 }+\gamma {\mathcal{L}}_{clustering}$$

where $P$ is the target distribution, $Q$ is the soft assignment where ${q}_{ij}$ is the probability of assigning sample $i$ to cluster $j$. $\alpha$, $\beta$ and $\gamma$ are hyper-parameters that control the trade-off between the components of the objective function. For these hyper-parameters, we employ commonly used parameters and minimize dataset-specific tuning as much as possible. In our experiment, $\alpha$ is set to 0.5, $\beta$ is set to 1, and $\gamma$ is set to 10.

### Clinical outcomes

With every hospital admission, we extracted information for demographics, 19 clinical biomarkers that are assessed upon admission (eTable 2), acuity scores for both Modified Early Warning Score (MEWS) and SOFA, and patient outcomes^31,32^. The primary outcomes were 30-day mortality and 3-year mortality, and the median duration until follow-up was 4.3 years, according to calculations using the reverse Kaplan–Meier method. The secondary outcomes consisted of admission to an intensive care unit (ICU) or intermediate care unit (IMC), mechanical ventilation (MV), acute kidney injury (AKI), sepsis, venous thromboembolism, and renal replacement therapy (RRT). Data processing and outcome calculation details are explained in the eMethods section.

### Statistical methods

To ascertain the optimal number of phenotypes with the dTIC approach, we evaluated a combination of phenotype size, Davies-Bouldin index^33^, silhouette score^34^, elbow method^35^, and gap statistic method^36^. Once the optimal phenotype number was ascertained, patterns of vital signs were visualized by using t-distribution stochastic neighbor embedding (t-SNE) plots, ranked plots that show phenotype pairwise mean standardized differences, line plots with 95% confidence intervals, alluvial plots, and chord diagrams (see eMethods for a comprehensive description).

We assessed the reproducibility of derived phenotypes by assessing their frequency distributions and associated clinical characteristics in the testing cohort. The phenotype assignments for the testing cohort were determined based on the minimum Euclidean distance between individual patients and the phenotype’s centroid (eMethods).

To compare phenotypes, we used the χ^2^ test for categorical variables and analysis of variance as well as the Kruskal–Wallis test for continuous variables. We used Kaplan–Meier curves with a right-censoring time of August 22, 2019 to illustrate overall survival and the log-rank test to compare overall survival. Comparisons of adjusted hazard ratios (HR) were made for all phenotypes by using Cox proportional-hazards regression while controlling for demographics, comorbidities, and acuity score when admitted. Using the Bonferroni correction, all *p* values were adjusted for multiple comparisons. To ensure that the phenotypes did not simply recapitulate existing acuity scores, we used alluvial plots and chord diagrams to compare the phenotypes to patients’ SOFA scores within 24 h of hospital admission. Both R version 3.5.1 and Python version 3.7 were used to perform analyses.

## Results

### Patients

All three cohorts were comparable in clinical characteristics, biomarker distributions, and outcomes (eTables 3 and 4)^14^. Across the cohorts, sex was equally distributed and the patients’ average age was 54 years old. Nearly two-thirds of the admissions were urgent admission, 18% of the patients were transfers from other hospitals, 27% were admitted to the hospital’s ICU or IMC, and 28% underwent surgery while admitted. Of the 27% of patients who were admitted to the ICU or IMC, 22–27% had high SOFA (> 6) or MEWS (> 4) scores when admitted. Of the 73% of patients who were admitted to hospital wards, only 2–3% had high acuity scores. For all cohorts, the 30-day mortality rate was 4% and the 3-year mortality rate was 19%.

### Derivation and characteristics of phenotypes

**Figure 2:**
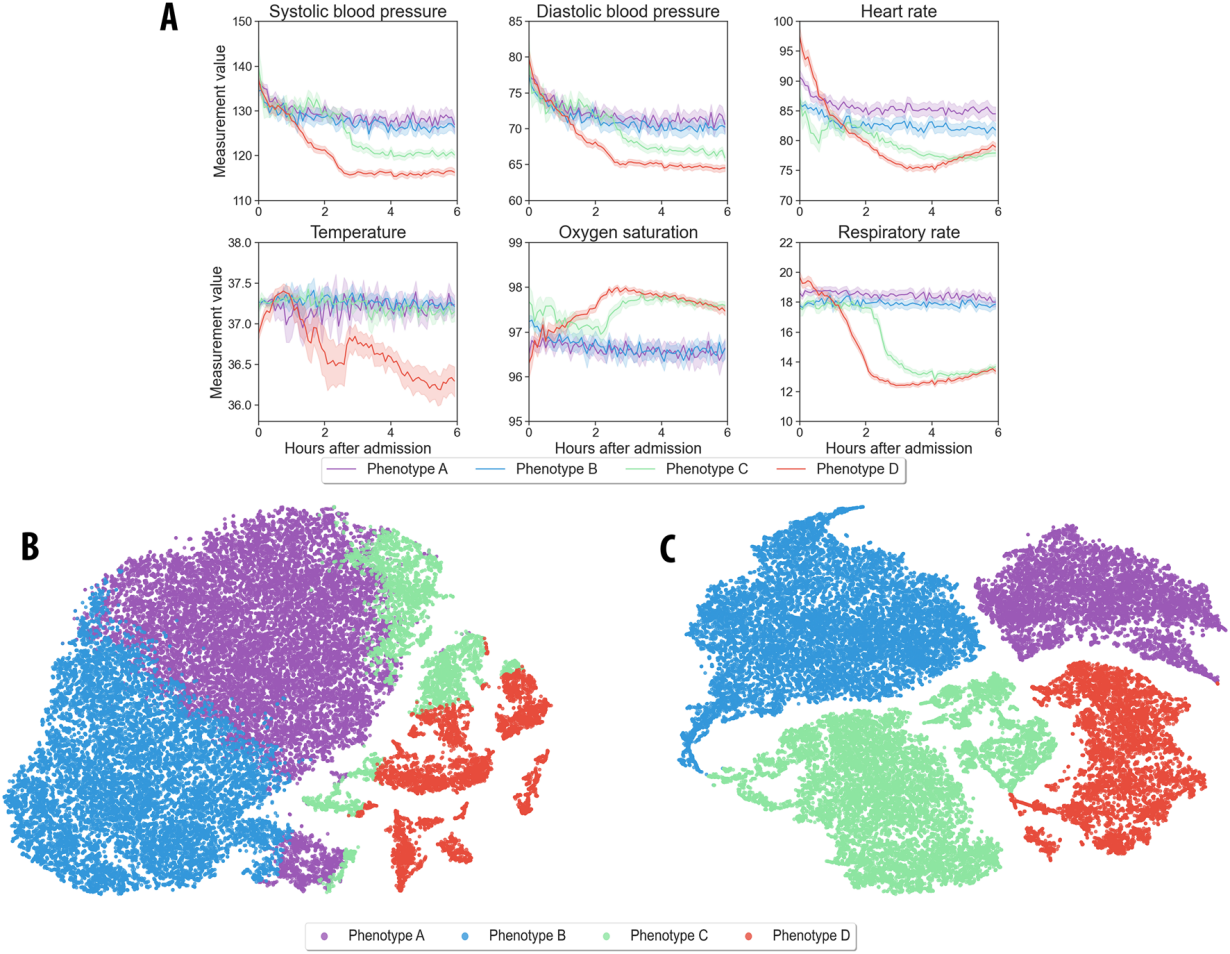
Vital sign representations across identified phenotypes. (**A**) Distribution of the vital signs recorded within the initial six hours following hospital admission. (**B**) Visualization of initial phenotypes, as assigned by the pre-trained deep temporal interpolation network, without the integration of the clustering network. The t-distributed stochastic neighbor embedding (t-SNE) technique was utilized to reduce the original 128-dimensional vital sign representations to two dimensions. Each dot signifies an individual patient, with separate colors indicating different phenotypes. (**C**) Visualization of final phenotypes, as assigned by the deep temporal interpolation and clustering network utilizing the t-SNE technique. The network simultaneously learns feature representation and cluster assignments, thus facilitating clustering-friendly representation learning.

In the training cohort, the dTIC model determined an optimal fit with a four-class model, optimizing a combination of metrics including phenotype size, Davies-Bouldin index, silhouette score, and elbow and gap statistic methods (eFig. 4 and eTable 5). These four phenotypes were associated with distinct pathophysiological signatures and clinical outcomes (Tables 1 and 2, eTables 6 and 7, Fig. 2). The phenotypes were categorized as phenotype A (18% of the cohort), B (33% of the cohort), C (31% of the cohort), and D (17% of the cohort) in relation to the descending systolic blood pressure value (Fig. 2A).

**Table 1:** Phenotype biomarkers and clinical characteristics.

Variables	Total	Acute illness phenotypes			
		Phenotype A	Phenotype B	Phenotype C	Phenotype D
Number of encounters (%)	41,502	7647 (18)	13,710 (33)	12,901 (31)	7244 (17)
**Clinical characteristics—preadmission**					
Age, mean (SD), years	54 (19)	57 (19)^a^	53 (19)^a^	51 (19)	57 (17)^a^
Female sex, n (%)	22,745 (55)	3963 (52)^a^	7595 (55)^a^	7391 (57)	3796 (52)^a^
Race, n (%)					
White	29,076 (70)	5203 (68)^a^	9421 (69)	9021 (70)	5431 (75)^a^
African American	9634 (23)	2036 (27)^a^	3411 (25)^a^	2930 (23)	1257 (17)^a^
Primary insurance, n (%)					
Private	9591 (23)	1323 (17)^a^	2917 (21)^a^	3314 (26)	2037 (28)^a^
Medicare	18,499 (45)	3839 (50)^a^	6120 (45)^a^	5158 (40)	3382 (47)^a^
Medicaid	9231 (22)	1641 (21)^a^	3213 (23)	3104 (24)	1273 (18)^a^
Uninsured	4181 (10)	844 (11)	1460 (11)	1325 (10)	552 (8)^a^
**Residing neighborhood characteristics**					
Distance from hospital (mile), median (IQR)	18 (3, 34)	14 (3, 27)^a^	14 (3, 32)^a^	18 (3, 36)	23 (9, 40)^a^
Proportion below poverty (%), mean (SD)	22.7 (10.1)	23.8 (10.1)^a^	23.1 (10.1)^a^	22.5 (10.0)	21.2 (9.8)^a^
Proportion of African Americans (%), mean (SD)	18.7 (17.5)	19.6 (17.8)^a^	19.3 (17.8)^a^	18.6 (17.5)	17.2 (16.1)^a^
**Comorbidities**					
Hypertension, n (%)	21,639 (52)	4129 (54)^a^	7205 (53)	6704 (52)	3601 (50)^a^
Chronic kidney disease, n (%)	6518 (16)	1450 (19)^a^	2467 (18)^a^	1802 (14)	799 (11)^a^
Diabetes mellitus, n (%)	10,111 (24)	1972 (26)^a^	3370 (25)	3100 (24)	1669 (23)
Cardiovascular disease, n (%)^b^	12,058 (29)	2413 (32)^a^	3991 (29)	3682 (29)	1972 (27)
**Admission characteristics of patients**					
Transfer from different hospital, n (%)	7115 (17)	1986 (26)^a^	3014 (22)^a^	1087 (8)	1028 (14)^a^
Emergent admission^c^, n (%)	30,177 (73)	7257 (95)^a^	11,764 (86)^a^	8064 (63)	3092 (43)^a^
**Diagnostic groups of primary admissions**					
Circulatory system diseases	7719 (19)	1934 (25)^a^	2425 (18)^a^	1834 (14)	1526 (21)^a^
Digestive and genitourinary system diseases	5184 (12)	789 (10)^a^	1686 (12)^a^	1876 (15)	833 (11)^a^
Infectious and respiratory diseases	3306 (8)	961 (13)^a^	1161 (8)^a^	705 (5)	479 (7)^a^
Musculoskeletal, connective tissue, and skin diseases	3651 (9)	317 (4)^a^	1042 (8)^a^	1222 (9)	1070 (15)^a^
Neoplasms	2743 (7)	93 (1)^a^	665 (5)^a^	1138 (9)	847 (12)^a^
Childbirth and pregnancy complications	3148 (8)	391 (5)^a^	1147 (8)^a^	1248 (10)	362 (5)^a^
**Clinical biomarkers and interventions within 24 h of admission**					
ICU or IMC admission within initial 24 h, n (%)	9426 (23)	2372 (31)^a^	1979 (14)	1921 (15)	3154 (44)^a^
Surgery on day admitted, n (%)	8644 (21)	272 (4)^a^	813 (6)^a^	3466 (27)	4093 (57)^a^
**Cardiovascular system**					
Hypertension (SBP > 160 mmHg) at any point, n (%)	14,838 (36)	2923 (38)^a^	3684 (27)^a^	4272 (33)	3959 (55)^a^
Duration, median (IQR), # of minutes	120 (27, 356)	174 (52, 445)^a^	214 (73, 477)^a^	114 (19, 336)	44 (9, 165)^a^
Hypotension (MAP < 60 mmHg) at any point, n (%)	14,470 (35)	2234 (29)^a^	2903 (21)^a^	4445 (34)	4888 (67)^a^
Duration, median (IQR), # of minutes	57 (15, 168)	86 (30, 224)^a^	92 (30, 233)^a^	33 (10, 120)	37 (10, 129)
Vasopressors used, n (%)	7531 (18)	421 (6)^a^	641 (5)^a^	2633 (20)	3836 (53)^a^
Outside of the operating room	1403 (3)	242 (3)^a^	146 (1)^a^	229 (2)	786 (11)^a^
Troponin, tested, n (%)	14,616 (35)	4502 (59)^a^	4862 (35)^a^	3055 (24)	2197 (30)^a^
Abnormal result in those tested, n (%)	3398 (23)	1109 (25)^a^	884 (18)	585 (19)	820 (37)^a^
**Respiratory system**					
Maximum administered FiO_2_, median (IQR), %	0.21 (0.21, 0.40)	0.21 (0.21, 0.29)^a^	0.21 (0.21, 0.28)^a^	0.21 (0.21, 0.40)	0.40 (0.29, 0.40)^a^
Room air only, n (%)	23,963 (58)	4615 (60)	10,130 (74)^a^	7874 (61)	1344 (19)^a^
0.22–0.40, n (%)	14,790 (36)	2496 (33)^a^	3252 (24)^a^	4484 (35)	4558 (63)^a^
> 0.40, n (%)	2749 (7)	536 (7)^a^	328 (2)^a^	543 (4)	1342 (19)^a^
P_a_O2/FiO_2_, tested with arterial blood gas, n (%)	6113 (15)	1352 (18)^a^	1033 (8)^a^	1273 (10)	2455 (34)^a^
< 200 in those tested, n (%)	2265 (37)	496 (37)	301 (29)	430 (34)	1038 (42)^a^
Mechanical ventilation, n (%)	2123 (5)	434 (6)^a^	191 (1)^a^	314 (2)	1184 (16)^a^
**Acid–base and kidney status**					
Preadmission estimated glomerular filtration rate^d^ (mL/min per 1.73 m^2^), median (IQR)	95 (78, 111)	93 (74, 109)^a^	96 (77, 111)^a^	97 (80, 113)	93 (79, 106)^a^
Maximum/reference creatinine^d^ ratio, mean (SD)	1.24 (0.66)	1.30 (0.70)^a^	1.22 (0.59)^a^	1.20 (0.67)	1.28 (0.74)^a^
Maximum anion gap, median (IQR), mmol/L	14 (12, 17)	15 (12, 18)^a^	14 (12, 16)	14 (11, 16)	15 (12, 18)^a^
Arterial blood gas tested, n (%)	6115 (15)	1353 (18)^a^	1033 (8)^a^	1274 (10)	2455 (34)^a^
Maximum base deficit among tested, mean (SD), mmol/L	4.8 (4.7)	5.4 (5.0)^a^	4.5 (4.6)	4.4 (4.3)	4.8 (4.6)
pH < 7.3 among tested, n (%)	1437 (23)	298 (22)	160 (15)	242 (19)	737 (30)^a^
Lactate, tested, n (%)	15,447 (37)	3935 (51)^a^	4308 (31)^a^	3706 (29)	3,498 (48)^a^
2–4 mmol/L among tested, n (%)	3,739 (24)	978 (25)	956 (22)	870 (23)	935 (27)^a^
> 4 mmol/L among tested, n (%)	1,374 (9)	331 (8)^a^	207 (5)	216 (6)	620 (18)^a^
Renal replacement therapy, n (%)	641 (2)	160 (2)^a^	168 (1)	175 (1)	138 (2)^a^
**Inflammation**					
Maximum white blood cell count, median (IQR), ×10^9^/L	9 (7, 13)	9 (7, 13)	9 (6, 12)^a^	9 (7, 12)	11 (8, 15)^a^
Maximum premature neutrophils (bands), median (IQR), %	10 (4, 20)	12 (4, 22)^a^	7 (3, 15)	9 (3, 18)	15 (7, 26)^a^
Minimum lymphocytes, median (IQR), %	16 (9, 24)	15 (8, 24)^a^	16 (10, 25)	17 (10, 26)	10 (6, 18)^a^
Erythrocyte sedimentation rate, tested, n (%)	3903 (9)	775 (10)	1591 (12)^a^	1253 (10)	284 (4)^a^
Maximum erythrocyte sedimentation rate, median (IQR), mm/h	40 (19, 73)	42 (18, 72)	41 (20, 75)	39 (19, 71)	32 (15, 66)
C-reactive protein, tested, n (%)	5862 (14)	1246 (16)^a^	2396 (17)^a^	1759 (14)	461 (6)^a^
Maximum C-reactive protein, median (IQR), mg/L	18 (5, 77)	20 (5, 89)^a^	18 (5, 73)^a^	15 (4, 70)	39 (7, 112)^a^
Minimum temperature, mean (SD), Celsius	36.7 (1.0)	36.7 (0.8)	36.8 (0.7)^a^	36.7 (0.7)	36.3 (1.7)^a^
Maximum temperature, mean (SD), Celsius	37.7 (0.6)	37.7 (0.6)	37.6 (0.6)^a^	37.7 (0.6)	37.9 (0.6)^a^
38–39, n (%)	8633 (21)	1519 (20)	2238 (16)^a^	2486 (19)	2390 (33)^a^
> 39, n (%)	1548 (4)	354 (5)^a^	453 (3)	368 (3)	373 (5)^a^
**Hematologic**					
Minimum hemoglobin, mean (SD), g/dL	11.5 (2.3)	11.6 (2.4)	11.7 (2.3)	11.6 (2.2)	10.9 (2.3)^a^
Maximum RDW, mean (SD), %	15.5 (2.1)	15.6 (2.1)^a^	15.6 (2.3)^a^	15.4 (2.1)	15.3 (1.9)
Minimum platelets, median (IQR), × 10^9^/L	210 (161, 269)	209 (160, 271)^a^	216 (165, 277)	214 (165, 270)	195 (150, 247)^a^
Platelets < 200, n (%), × 10^9^/L	16,707 (40)	3289 (43)^a^	5347 (39)^a^	4769 (37)	3302 (46)^a^
< 100	2643 (16)	526 (7)	910 (7)^a^	715 (6)	492 (7)
100–200	14,064 (84)	2763 (84)	4437 (83)^a^	4054 (85)	2810 (85)
International normalized ratio, tested, n (%)	20,357 (49)	4830 (63)^a^	6942 (51)^a^	5201 (40)	3384 (47)^a^
≥ 2	1836 (9)	432 (9)	666 (10)	433 (8)	305 (9)
**Neurologic**					
Glasgow Coma Scale (GCS) score, n (%)					
Severe neurologic dysfunction (≤ 8)	1482 (4)	359 (5)^a^	144 (1)^a^	207 (2)	772 (11)^a^
Moderate neurologic dysfunction (9–12)	1708 (4)	385 (5)^a^	340 (2)	284 (2)	699 (10)^a^
**Liver and metabolic**					
Maximum glucose, median (IQR), mg/dL	126 (104, 170)	127 (105, 175)^a^	120 (101, 161)	123 (101, 164)	144 (116, 187)^a^
Bilirubin tested, n (%), mg/dL	21,183 (51)	4759 (62)^a^	8018 (58)^a^	5894 (46)	2512 (35)^a^
≥ 2	1427 (7)	271 (6)	542 (7)	395 (7)	219 (9)^a^
Albumin, tested, n (%)	21,368 (51)	4780 (63)^a^	8070 (59)^a^	5951 (46)	2567 (35)^a^
< 2.5	1243 (6)	304 (6)^a^	419 (5)	260 (4)	260 (10)^a^
2.5–3.5	6904 (32)	1678 (35)^a^	2515 (31)^a^	1719 (29)	992 (39)^a^

ICU: intensive care unit; IMC: intermediate care unit; MAP: mean arterial pressure; SBP: systolic blood pressure; SD: standard deviation; RDW: red cell distribution width; IQR: interquartile range.

^a^The p values represent significant differences (p < 0.05) compared to phenotype C, adjusted for multiple comparisons using the Bonferroni method. The supplementary tables provide p values for every comparison within groups.

^b^For the consideration of cardiovascular disease, the following were taking into consideration: history of congestive heart failure, peripheral vascular disease, and coronary artery disease.

^c^Emergent admissions include emergent admissions, urgent admissions and trauma center admissions.

^d^Race correction was not applied to derive the reference glomerular filtration rate and reference creatinine (refer to eMethods for further information).

**Table 2:** Phenotype illness severity, resource use, and clinical outcomes.

Variables	Total	Acute illness phenotypes			
		Phenotype A	Phenotype B	Phenotype C	Phenotype D
Number of encounters (%)	41,502	7647 (18)	13,710 (33)	12,901 (31)	7244 (17)
**Acuity scores, first 24 h of admission**					
SOFA score > 6, n (%)	3506 (8)	656 (9)^a^	508 (4)^a^	768 (6)	1574 (22)^a^
ICU or IMC patients, SOFA score ≤ 6, n (%)	6882 (17)	1822 (24)^a^	1690 (12)	1514 (12)	1856 (26)^a^
ICU or IMC w/ SOFA score > 6, n (%)	2544 (6)	550 (7)^a^	289 (2)^a^	407 (3)	1298 (18)^a^
Ward w/ SOFA score ≤ 6, n (%)	31,114 (75)	5169 (68)^a^	11,512 (84)^a^	10,619 (82)	3814 (53)^a^
Ward w/ SOFA score > 6, n (%)	962 (2)	106 (1)^a^	219 (2)^a^	361 (3)	276 (4)^a^
MEWS score ≥ 5, n (%)	2828 (7)	873 (11)^a^	575 (4)^a^	387 (3)	993 (14)^a^
ICU or IMC w/MEWS score ≤ 4, n (%)	7235 (17)	1703 (22)^a^	1618 (12)	1643 (13)	2271 (31)^a^
ICU or IMC w/MEWS score > 4, n (%)	2191 (5)	669 (9)^a^	361 (3)	278 (2)	883 (12)^a^
Ward w/MEWS score ≤ 4, n (%)	31,439 (76)	5071 (66)^a^	11,517 (84)	10,871 (84)	3980 (55)^a^
Ward w/MEWS score > 4, n (%)	637 (2)	204 (3)^a^	214 (2)^a^	109 (1)	110 (2)^a^
**Resource use throughout hospitalization**					
Days in hospital, median (IQR)	4 (2, 7)	4 (2, 7)^a^	4 (2, 7)^a^	3 (2, 6)	4 (3, 7)^a^
Surgery during hospital stay, n (%)	11,634 (28)	860 (11)^a^	2006 (15)^a^	4452 (35)	4316 (60)^a^
ICU or IMC^b^ admission, n (%)	11,121 (27)	2700 (35)^a^	2673 (19)	2446 (19)	3302 (46)^a^
Days in ICU or IMC^c^, median (IQR)	4 (2, 7)	4 (3, 7)^a^	4 (3, 7)^a^	4 (2, 6)	4 (3, 8)^a^
More than 48 h in ICU/IMC^c^, n (%)	8332 (75)	2068 (77)^a^	2008 (75)^a^	1751 (72)	2505 (76)^a^
Mechanical ventilation, n (%)	3218 (8)	695 (9)^a^	554 (4)^a^	628 (5)	1341 (19)^a^
Hours on mechanical ventilation^d^, median (IQR)	35 (14, 113)	44 (17, 127)^a^	35 (13, 116)	25 (11, 101)	33 (14, 113)
More than 48 h on mechanical ventilation^d^, n (%)	1661 (52)	403 (58)^a^	284 (51)	302 (48)	672 (50)
Renal replacement therapy (RRT), n (%)	1262 (3)	314 (4)^a^	396 (3)	322 (2)	230 (3)^a^
**Complications**					
Acute kidney injury (AKI), n (%)	6905 (17)	1598 (21)^a^	2279 (17)^a^	1680 (13)	1348 (19)^a^
Community-acquired AKI, n (%)	3839 (56)	924 (58)	1200 (53)	897 (53)	818 (61)^a^
Hospital-acquired AKI, n (%)	3066 (44)	674 (42)	1079 (47)	783 (47)	530 (39)^a^
Worst AKI staging, n (%)					
Stage 1	4360 (63)	961 (60)^a^	1479 (65)	1112 (66)	808 (60)^a^
Stage 2	1362 (20)	346 (22)^a^	425 (19)	300 (18)	291 (22)
Stage 3	848 (12)	206 (13)	270 (12)	202 (12)	170 (13)
Stage 3 with RRT	335 (5)	85 (5)	105 (5)	66 (4)	79 (6)
Venous thromboembolism, n (%)	1257 (3)	261 (3)^a^	481 (4)^a^	334 (3)	181 (2)
Sepsis, n (%)	3750 (9)	1049 (14)^a^	1102 (8)^a^	754 (6)	845 (12)^a^
Hospital disposition, n (%)					
Hospital mortality	1141 (3)	294 (4)^a^	278 (2)^a^	184 (1)	385 (5)^a^
Different, LTAC, SNF, or Hospice	4475 (11)	1140 (15)^a^	1591 (12)^a^	932 (7)	812 (11)^a^
Short-term rehabilitation or home	35,886 (86)	6213 (81)^a^	11,841 (86)^a^	11,785 (91)	6047 (83)^a^
30-day mortality, n (%)	1633 (3.9)	439 (6)^a^	458 (3)^a^	278 (2)	458 (6)^a^
Three-year mortality, n (%)	8013 (19)	1892 (25)^a^	2861 (21)^a^	1975 (15)	1285 (18)^a^

ICU: intensive care unit; IMC: intermediate care unit; IQR: interquartile range; MEWS: modified early warning score; SOFA: sequential organ failure assessment.

^a^The p values represent significant differences (p < 0.05) compared to phenotype C, adjusted for multiple comparisons using the Bonferroni method. The supplementary tables provide p values for all comparisons within groups.

^b^ICU/IMC admission rate was derived from data collected at any point during the hospitalization period. When an encounter had multiple ICU admissions throughout the hospitalization, the admission rate will only be evaluated once.

^c^The length of ICU stay and prolonged ICU stay (greater than 48 h) were derived for only patients who were admitted to the ICU or IMC.

^d^The duration of receiving mechanical ventilation and prolonged mechanical ventilation (greater than 48 h) were derived for only patients who required mechanical ventilation.

#### Phenotype A

Phenotype A had the greatest burden of comorbid disease, such as hypertension (54%) and cardiovascular disease (32%), the highest proportion of African American race (27% vs. 17–25% in other phenotypes) and emergent admissions (95%). Phenotype A had the highest rate of prolonged respiratory insufficiency (9% received MV, 58% of whom received ventilator support for more than 2 days), AKI (21%), sepsis (14%), and 3-year mortality (25%). Patients in phenotype A had the second greatest incidence of admission to ICU/IMC (35%), hospital mortality (4%), and 30-day mortality (6%).

#### Phenotype B

Phenotype B exhibited physiological signatures similar to those of phenotype A, but displayed a diffuse pattern of mild organ dysfunction with persistent, uncorrected blood pressure abnormalities during the first six hours. Phenotype B had favorable short-term clinical outcomes, manifested by the second lowest rates of ICU or IMC admission (19%), AKI (17%), sepsis (8%), hospital mortality (2%), and 30-day mortality (3%). Phenotype B corresponded to the second highest rate of 3-year mortality.

#### Phenotype C

Phenotype C exhibited low early physiological derangement with a diffuse pattern of mild organ dysfunction. Phenotype C exhibited favorable clinical outcomes, which manifested as the lowest rate of ICU/IMC admission (19%), AKI (13%), sepsis (6%), hospital mortality (1%), 30-day mortality (2%), and 3-year mortality (15%). They had the second highest rate of surgery within 24 h of admission (27%) but similar rates of admission to wards as phenotype B.

#### Phenotype D

Phenotype D was characterized by early and persistent hypotension, a high incidence of vasopressor support (53%), and the highest proportion requiring early surgery (57%). Phenotype D had significant biomarker incidence of inflammation, evidenced by the highest median white blood cell count (11 × 10^9^/L compared with 9 × 10^9^/L in other phenotypes), premature neutrophils (15% vs. 7–12% in other phenotypes), and C-reactive protein (39 mg/L vs. 15–20 mg/L in other phenotypes); and the lowest median lymphocytes (10%). Phenotype D had the highest rate of ICU/IMC admission (46%), MV (19%), hospital mortality (5%), and 30-day mortality (6%). They had the second highest incidence of AKI (19%) and sepsis (12%). In spite of early and severe illness, phenotype D had favorable long-term outcomes with the second lowest 3-year mortality (18%).

### Patterns of vital signs

**Figure 3:**
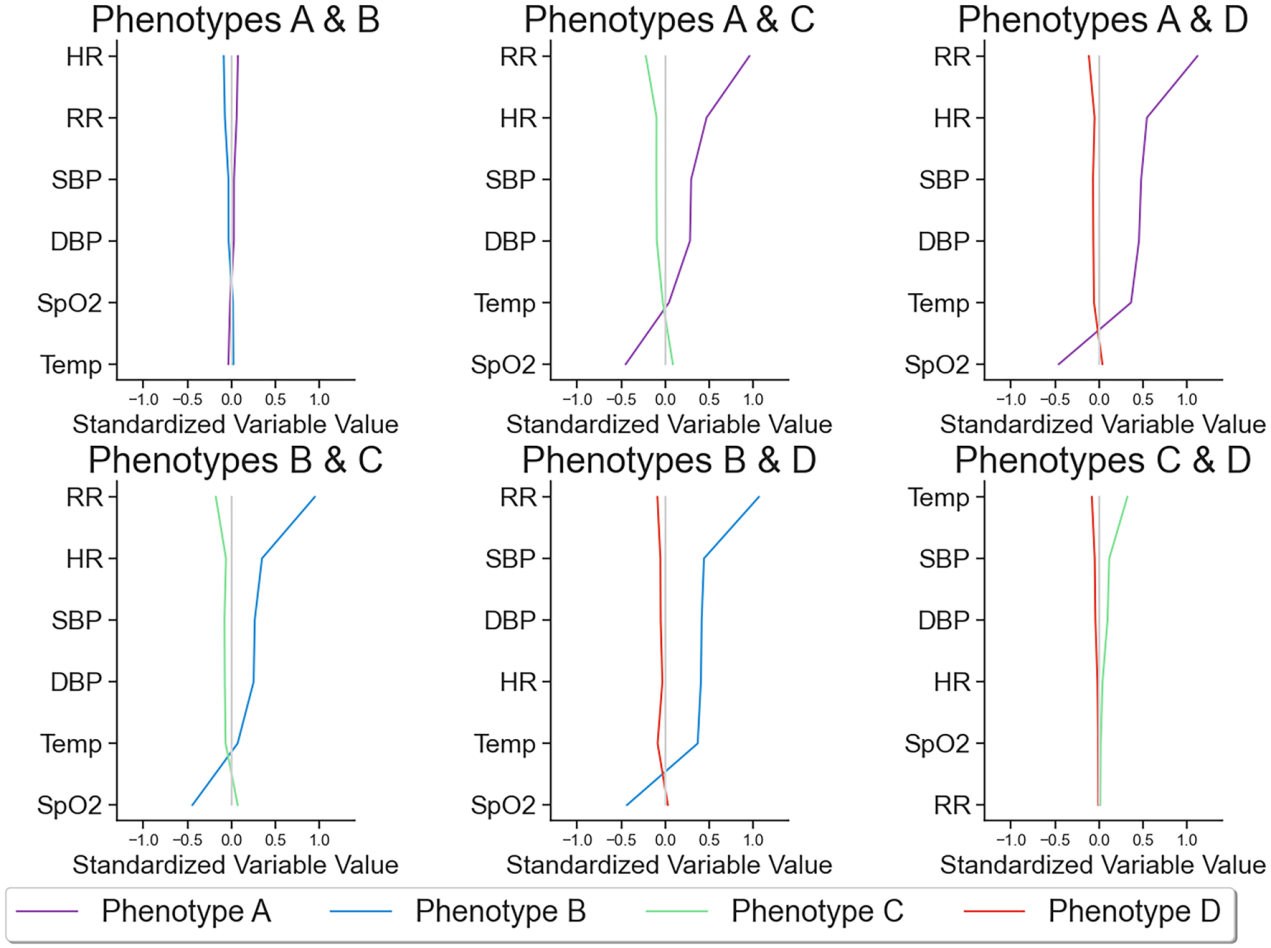
Contributions of vital signs to assigned clusters. The pairwise phenotype comparisons of vital sign values, which have been standardized to a mean of 0 and standard deviation of 1. The comparison reveals that oxygen saturation and temperature contribute the least to the differences between phenotypes. Conversely, respiratory rate and heart rate exhibit considerable variation across all phenotypes, with the exception of phenotypes C and D, where these vital signs appear more consistent. Temp: temperature; SpO2: peripheral capillary oxygen saturation; DBP: diastolic blood pressure; SBP: systolic blood pressure; RR: respiratory rate; HR: heart rate.

In order to determine which vital signs had the most notable effect on cluster designations, we compared the standardized mean differences between pairs of phenotypes (Fig. 3). The smallest contributors to the differences in phenotypes were temperature and oxygen saturation. Respiratory rate and heart rate differed significantly across phenotypes except for C and D, which manifested as differences in temperature.

### Relationship with organ support and admission diagnoses

The association between phenotypes and the highest SOFA score recorded within 24 h after admission is depicted in eFig. 5; the SOFA components for every phenotype are depicted with chord diagrams in eFig. 6. The highest percentages of patients with cardiovascular and respiratory dysfunction were found in Phenotypes C and D; however, every phenotype had significant percentages of patients from the entire spectrum of SOFA scores and component subscores; the clustering into phenotypes did not only restate prior SOFA acuity assessments.

The frequency distribution of admission diagnoses across phenotypes is shown in Table 1, eFig. 7 and eTable 8. Nonspecific chest pain was the most common diagnosis in phenotypes A and B, with abdominal pain being most frequent in phenotype C, and osteoarthritis in phenotype D. However, we observed that no specific presentation or group of diagnoses (cardiac, trauma, intra-abdominal, or musculoskeletal) appeared disproportionately in one phenotype over another (see eFig. 7 and eTable 8).

### Relationship with survival probabilities

**Figure 4:**
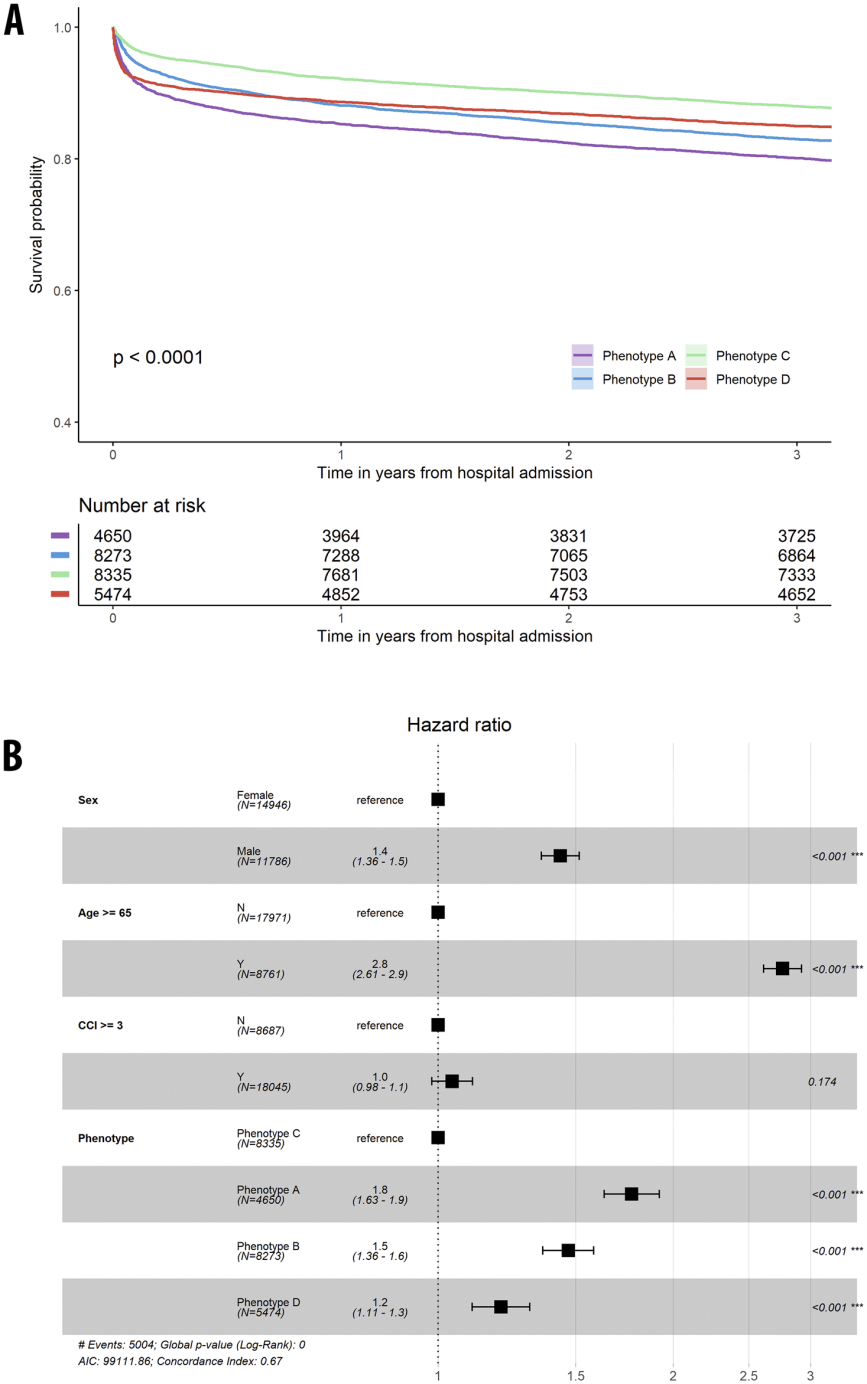
Survival curves and adjusted Cox proportional hazards modeling. (**A**) The survival curves for each phenotype, considering adjustments for both comorbidities and demographic information. (**B**) The adjusted Cox proportional hazards models, incorporating comorbidities and demographic information into the analysis. CCI: Charlson Comorbidity Index.

Long-term survival (3 years) adjusted for demographics and comorbidities (Fig. 4, eFig. 8A and B) was significantly lower for males (HR 1.4, 95% CI 1.4–1.5) and for patients 65 years or older (HR 2.8, 95% CI 2.6–2.9). With phenotype C as reference, survival probability was lower for phenotype A (HR 1.8, 95% CI 1.6–1.9), B (HR 1.5, 95% CI 1.4–1.6), and D (H 1.2, 95% CI 1.1–1.3, all p < 0.001). Similar 3-year survival probability adjusted for additional SOFA score was modeled (eFig. 8C and D), demonstrating substantial associations between higher SOFA score and lower survival probability (SOFA 2–4: HR 1.8, 95% CI 1.6–1.9; SOFA 5 or greater: HR 3.1, 95% CI 2.8–3.3, all p < 0.001). After being adjusted for SOFA, phenotypes A and B had much stronger associations with lower survival probability (HR 1.9, 95% CI 1.8–2.1; HR 1.7, 95% CI 1.6–1.9, all p < 0.001), but phenotype D had a stronger association with improved survival probability (HR 0.9, 95% CI 0.8–1.0, p = 0.035).

Three-year survival probability for clinical entities of sepsis, AKI, and surgical patients were modeled after adjusting for demographics and comorbidities (eFigs. 9–11). Using phenotype C as a reference, the survival probability for patients with sepsis was found to be lower for phenotype A (HR 1.4, 95% CI 1.2–1.7, p < 0.001), B (HR 1.1, 95% CI 0.9–1.3, p = 0.27), and D (HR 1.7, 95% CI 1.4–2.0, p < 0.001); for patients with AKI, probability of survival was lower for phenotype A (HR 1.3, 95% CI 1.1–1.4, p < 0.001), B (HR 1.1, 95% CI 1.0–1.3, p = 0.052), and D (HR 1.4, 95% CI 1.2–1.6, p < 0.001); for patients undergoing surgery, probability of survival was lower for phenotype A (HR 2.1, 95% CI 1.7–2.6) and B (HR 1.8, 95% CI 1.6–2.2, all p < 0.001), but higher for phenotype D (HR 0.9, 95% CI 0.8–1.1, p = 0.349).

### Reproducibility

In the training and testing cohorts, the percentage of patients in each phenotype was stable (phenotype A: 18% and 18%; phenotype B: 33% and 35%; phenotype C: 31% and 31%; phenotype D: 17% and 16%). Phenotypes were reproducible in the testing cohort. Within the testing cohort, the phenotypes were similar to the training cohort in terms of clinical characteristics, biomarkers, and patient outcomes (eFigs. 12 and 13, eTables 9 and 10). Across the training and testing cohorts, there were similar distributions of SOFA scores, survival scores, and diagnosis groups (eFigs. 14–17, eTable 11).

### Evaluation of representation learning of deep interpolation network

The efficacy of the dTIC network in learning cluster-friendly feature representations from sparse, irregularly sampled time-series data was clearly depicted through a visual representation of acuity illness phenotypes using t-SNE (Fig. 2B and C). In the raw data space, phenotypes, particularly phenotype A and phenotype B, appeared visually indistinguishable (Fig. 2A). Utilizing the pre-trained dTIC network, which does not incorporate the clustering network, to transform low-dimensional data into a non-linear, high-dimensional feature space, we obtained phenotypes that were separable in general (Fig. 2B). By integrating the clustering network with pre-trained network and training them jointly, we achieved a feature representation more conducive to clustering, as illustrated in Fig. 2C.

The reconstruction error of physiologic signatures, presented in eFigs. 18 and 19 demonstrated that the dTIC network can accurately regenerate the input physiologic signatures across all vital signs, where the disparity between the observed and reconstructed data was negligible.

## Discussion

To the best of our knowledge, this is the first instance of using deep interpolation network clustering to phenotype a diverse cohort of hospitalized patients based on early vital sign measurements, a universally available measure of patient acuity that can be leveraged to make timely triage decisions. Although previous studies have demonstrated the efficacy of clustering in identifying patient subgroups within larger cohorts with similar clinical presentations, such as sepsis and diastolic heart failure^10,37^, this approach of employing a deep interpolation network is unique.

Our group has also previously employed consensus clustering to early vital sign measurements and distinguished four phenotypes, albeit without the use of a deep interpolation network^14^. In that study, each vital sign sequence was resampled on an hourly basis by averaging the multiple measurements within a one-hour window and imputing the missing vital signs through forward and backward propagation. Resampling techniques for processing irregularly sampled vital signs could introduce bias, however, since the frequency of measurements and dynamic patterns of vitals within each one-hour window is ignored. Unlike the previous approach, this present study fully capitalizes on irregularly sampled time-series circumventing the error-prone resampling. Both studies identified a phenotype with highest incidence of sepsis, prolonged respiratory insufficiency, AKI, and 3-year mortality. Moreover, both identified a phenotype exhibiting early and persistent hypotension along with a need for early surgery. Several differences emerged, however, when comparing the identified phenotypes, especially in our study. Phenotype D exhibited worse short-term clinical outcomes. These comparative findings suggest that deep representation of irregularly sampled early vital signs can potentially unveil diverse patient subgroups, differing from what conventional machine learning approaches might reveal.

Other researchers have also investigated patient phenotyping. Seymour et al.^10^ carried out clustering analyses on septic patients, hypothesizing that the pathophysiology of sepsis is inherently heterogeneous and recognizing that targeted therapies may be enabled by distinct sepsis phenotypes. The inconclusiveness of most sepsis drug trials supports this rationale. The clustering in the Seymour et al. study was performed on clinical variables and immune response biomarkers, resulting in the identification of four distinct clusters. They conducted simulations in which varying proportions of each cluster were introduced to previously reported randomized controlled trials, indicating unique treatment responses across different clusters. Along these same lines, Shah et al.^37^ executed clustering analyses for patients with preserved ejection fraction and heart failure. For their clustering, Shah et al. used a combination of echocardiogram and electrocardiogram data in addition to clinical variables, identifying three unique phenotypes with distinct clinical outcomes. This was true even after accounting for traditional risk factors. Findings such as these suggest that clustering methods have the capacity to distinguish subgroups of patient phenotypes that traditional clinical parameters might not be able to identify, and these subgroups could potentially exhibit different clinical outcomes and treatment responses.

The present study attempts to make use of real-world data available to clinicians at the time of initial patient assessment. Vital signs, while sparse, are affordable and universally obtained. Current practice often relies on clinical gestalt to assess patient acuity and project a hospital course and patients often are first encountered by healthcare providers in training or those with less experience. Having readily available phenotypes may improve triage decisions. Among the four identified phenotypes, phenotype D appeared to represent patients who were demonstrably ill upon admission and received appropriate level of care with surgical source control or correction of their underlying pathology, consequently exhibited improved 30-day and 3-year survival. Phenotype A also appeared to have a high degree of illness severity, though this was likely related to acute on chronic exacerbation of underlying comorbidities. These patients typically present a clinical challenge to their healthcare providers. Phenotypes B and C had less acuity of illness and were most often admitted to general wards. Phenotype C underwent surgery more often, possibly representing patients with urgent, but not emergent, correctable surgical and medical pathology. Phenotype B, much like phenotype A, may include patients with chronic comorbidities though of less severity.

Our study has several limitations. First, the potential to generalize our findings may be somewhat limited due to our study’s reliance on data from a single institution. While it is recognized that patient populations can vary across different institutions, we argue that vital signs, being a direct expression of physiological status, would maintain consistency across diverse healthcare contexts. Second, for this study, we confined our input features to vital signs from the first six hours following hospital admission. Minimum sampling time of data balancing accurate patient stratification with the need for timely decision-making, which is crucial for the implementation of decision support system, has not yet been thoroughly investigated in our study. In addition, it is important to note that critical lab results and imaging findings, which can substantially impact patient clustering, are frequently available within this timeframe. Future iterations of our model will explore the optimal setting of minimum sampling time of data and potential advantages of incorporating other data for a more reliable and comprehensive understanding of patient physiology. Last, the potential of early clustering to supplement clinical prognosis and decision-making, though promising, is still largely theoretical until the clustering can be evaluated in the setting of a prospective clinical trial.

## Conclusions

For this study, we developed and evaluated a novel, deep temporal interpolation and clustering network for extracting latent representations from time-series data that was sparse and sampled irregularly—specifically, vital sign measurements obtained during the first six hours of hospital admission—and identified a total of four unique patient phenotypes. Each phenotype exhibited distinct pathophysiological signatures and associated clinical outcomes, and did not simply repeat known, recognized clinical phenotypes, such as SOFA score. Our algorithm has the potential to significantly enhance early clinical decision-making, such as triage decisions, especially in situations where data availability is limited. Future efforts will focus on incorporating this model with historical patient data and additional elements from the EHR, along with external validation of these findings in clinical trials.

### Supplementary Information

Supplementary Information.

## Data Availability

The vitals data used in this analysis include both date and time stamps. In order to prevent patient privacy compromises because of identifiers in the data, our data cannot be publicly shared in a repository. Upon reasonable request, data can be shared by the University of Florida Integrated Data Repository at IRBDataRequest@ahc.ufl.edu and the University of Florida Intelligent Clinical Care Center at ic3-center@ufl.edu.

## Code availability

The programming code used in our analysis is provided on GitHub for open access and can be found at https://github.com/Prisma-pResearch/Deep_Interpolation_Clustering.
