# Supplementary material for: Identifying acute illness phenotypes via deep temporal interpolation and clustering network on physiologic signatures
Source: Sci Rep. 2024 Apr 10;14:8442. doi: 10.1038/s41598-024-59047-x (PMC11006654; doi:10.1038/s41598-024-59047-x)
Supplement: Supplementary file 1 — Supplementary Information. [file 41598_2024_59047_MOESM1_ESM.docx]

**Supplementary Online Content**

Yuanfang Ren, Yanjun Li, Tyler J. Loftus et al. Physiologic signatures within six hours of hospitalization identify acute illness phenotypes via deep temporal interpolation and clustering network.

This supplementary material has been provided by the authors to give readers additional information about their work.

[eMethods 3](#_Toc162257722)

[eFigure 1. Cohort selection and exclusion criteria 7](#_Toc162257723)

[eFigure 2. Spearman correlation heat map for the training cohort (N=41,502) 8](#_Toc162257724)

[eFigure 3. Deep temporal interpolation and clustering network algorithm 9](#_Toc162257725)

[eFigure 4. Gap statistic and elbow approaches showing the optimal number of clusters 10](#_Toc162257726)

[eFigure 5. Alluvial plot showing distribution of phenotypes across worst SOFA scores of patients within first 24 hours of admission in the training cohort 11](#_Toc162257727)

[eFigure 6. Chord diagrams showing the distribution of patients with higher SOFA scores (i.e., 2+) within first 24 hours of admission of six organ systems by phenotypes in the training cohort 12](#_Toc162257728)

[eFigure 7. Chord diagrams showing the distribution of nine most common admission diagnosis groups by phenotype in the training cohort 13](#_Toc162257729)

[eFigure 8. Survival curves and Cox proportional hazards modeling by phenotypes in the training cohort 14](#_Toc162257730)

[eFigure 9. Survival curves and Cox proportional hazards modeling by sepsis patients in the training cohort 15](#_Toc162257731)

[eFigure 10. Survival curves and Cox proportional hazards modeling by AKI patients in training cohort 16](#_Toc162257732)

[eFigure 11. Survival curves and Cox proportional hazards modeling by surgical patients in training cohort 17](#_Toc162257733)

[eFigure 12. Distribution of vital signs during the first six hours of hospital admission in the testing cohort 18](#_Toc162257734)

[eFigure 13. t-SNE plot of phenotype assignments in the testing cohort 19](#_Toc162257735)

[eFigure 14. Alluvial plot showing distribution of phenotypes across worst SOFA scores of patients within first 24 hours of admission in the testing cohort 20](#_Toc162257736)

[eFigure 15. Chord diagrams showing the distribution of patients with higher SOFA scores (i.e., 2+) within first 24 hours of admission of six organ systems by phenotypes in the testing cohort 21](#_Toc162257737)

[eFigure 16. Survival curves and Cox proportional hazards modeling by phenotypes in the testing cohort 22](#_Toc162257738)

[eFigure 17. Chord diagrams showing the distribution of nine most common admission diagnosis groups by phenotype in the testing cohort 23](#_Toc162257739)

[eFigure 18. Reconstruction error of physiologic signatures measured within six hours of hospital admission in training cohort using deep temporal interpolation and clustering network 24](#_Toc162257740)

[eFigure 19. Reconstruction error of physiologic signatures measured within six hours of hospital admission in testing cohort using deep temporal interpolation and clustering network 25](#_Toc162257741)

[eTable 1. Processing of vital sign time series 26](#_Toc162257742)

[eTable 2. Used LOINCS, range of values, direction of abnormal values for lab variables 27](#_Toc162257743)

[eTable 3. Clinic characteristics and biomarkers of the cohorts 31](#_Toc162257744)

[eTable 4. Illness severity, clinical outcomes, and resource use of the cohorts 35](#_Toc162257745)

[eTable 5. Statistic output from the deep interpolation network modeling in the training cohort. 37](#_Toc162257746)

[eTable 6. Phenotype clinical characteristics and biomarkers in the training cohort 38](#_Toc162257747)

[eTable 7. Phenotype illness severity, clinical outcomes, and resource use in the training cohort 42](#_Toc162257748)

[eTable 8. Frequency distribution of admission diagnoses across phenotypes in the training cohort 44](#_Toc162257749)

[^*^ The classification of diagnosis code was generated based on clinical classification software for ICD-10-CM v2019.1^19^. eTable 9. Phenotype clinical characteristics and biomarkers in the testing cohort 48](#_Toc162257750)

[eTable 10. Phenotype illness severity, clinical outcomes, and resource use in the testing cohort 53](#_Toc162257751)

[eTable 11. Frequency distribution of admission diagnoses across phenotypes in the testing cohort 55](#_Toc162257752)

# eMethods

1. **Data source and participants and study design**

**Data source and participants**

This project was approved by the University of Florida institutional review board under a waiver of informed consent and with authorization under the Health Insurance Portability and Accountability Act. Transparent Reporting of a multivariable prediction model for Individual Prognosis Or Diagnosis (TRIPOD) recommendations were followed under the Type 2b analysis category (nonrandom split-sample development and validation). Using the University of Florida Health (UFH) Integrated Data Repository as Honest Broker, we created a longitudinal dataset from electronic health records of all adults (age ≥18 years) admitted to the 1000-bed academic hospital at UFH between June 1, 2014 and April 1, 2016. The dataset includes structured and unstructured clinical data, demographic information, vital signs, laboratory values, medications, diagnoses, and procedures. Patients completely missing at least two of the six vital sign measurements used for clustering (systolic and diastolic blood pressure, heart rate, respiratory rate, temperature, and peripheral capillary oxygen saturation) in the first six hours of admission were excluded from the analysis (eFigure 1). The final cohort consisted of 75,762 hospital admissions for 43,598 patients.

**Study design**

We non-randomly split the dataset by admission dates into three cohorts: training (admissions between June 1, 2014 and May 31, 2015, n = 41,502, 55% of all admissions), validation (admissions between June 1, 2015 and October 31, 2015, n = 17,415, 22% of all admissions), and testing (admissions between November 1, 2015 and April 1, 2016, n = 16,845, 23% of all admissions). To determine acute illness phenotypes using early physiologic signatures, we derived the clinical phenotypes using unsupervised clustering methods that were applied to the repeated measurements of six vital signs available within the first six hours of hospital presentation in the training cohort. We selected hyper-parameters of clustering model using validation cohort. We assessed phenotype reproducibility by predicting phenotypes in the testing cohort and assessing phenotype frequency distributions and clinical outcomes.

1. **Approach to preprocess electronics health records (EHR) data**

EHR vital data elements in our cohort studies were irregularly sampled time series. Prior to clustering algorithms, we excluded outliers based on the expert-defined ranges (eTable 1). For the time series missing entirely, which is due to having no measurements during the hospitalization in the plausible range for a variable, we assigned the starting point (time t=0) value of the time series to the mean value of corresponding variables in the training cohort, as listed in eTable 1. We standardized values using Min-Max scaler (eTable 1). We directly input these irregular sampled time series to our clustering algorithm without any time interval imputation.

1. **Data visualization**
   - Chord plots

Chord diagram is widely used to represent connection and relationship between several entities. We generated two sets of chord diagrams to visualize the patients’ distribution regarding different studied variables.

One set of chord diagrams were created to visualize the distribution of phenotypes across worst SOFA scores of six organ systems within first 24 hours of admission. These six organ systems include:

- - - Cardiovascular
    - Respiratory
    - Coagulation
    - Liver
    - Neurologic
    - Renal

For each organ system, percent of patients with organ dysfunction, that is with SOFA score of 2 or more were calculated. For each phenotype, the larger percent of patients with higher score of that organ system, the border the ribbon. Phenotypes are shown in separate colors.

The other set of chord diagrams were created to visualize the distribution of nine most common admission diagnosis groups by phenotypes. These most common admission diagnosis groups vary from cohorts, including:

- - - Nonspecific chest pain
    - Abdominal pain
    - Complication of device; implant or graft
    - Other and unspecific lower respiratory disease
    - Septicemia (except in labor)
    - Acute cerebrovascular disease
    - Cardiac dysrhythmias.
    - Congestive heart failure; nonhypertensive
    - Malaise and fatigue
    - Osteoarthritis
    - Other complications of pregnancy

For each phenotype, the larger percentage of patients with that admission diagnosis group, the border the ribbon. Phenotypes are shown in separate colors. Diagrams were generated with Circlize R package.^1^

- - Alluvial plots

Alluvial plots were generated to visualize distribution of phenotypes across worst Sequential Organ Failure Assessment Score (SOFA) scores of patients within first 24 hours of admission. Phenotypes were grouped in the left column and the total SOFA scores were categorized into 3 levels (0-1, 2-4 and 5+) listed in the right column. Ribbons connect the phenotypes and SOFA categories, which indicates a percentage of patients in a phenotype fall into a particular SOFA category and vice versa. The larger percentage of patients, the boarder the ribbon. Phenotypes are shown in separate colors. Plots were generated with Alluvial R package.^2^

- - t-SNE plots

t-Distributed Stochastic Neighbor Embedding (t-SNE) is a nonlinear dimensionality reduction technique well-suited for embedding high-dimensional data for visualization in a low-dimensional space. In our work, the t-SNE plots depicted the 2 dimensional feature space of the patients’ vital signs after reducing their original dimension from 36 to 2 by t-SNE algorithm. Each dot represents a patient, and patients in different phenotypes are colored differently. Plots were generated by scikit-learn t-SNE Python package.^3^

- - Line plots

Line plots were generated to visualize the time-series vital sign data as it is well-suited for analyzing trends of different variables along time. We created a line plot for each vital sign studied in our work. To better observe the trends of different vital signs, for each encounter, we resampled the raw time series data to 5-minute frequency by averaging multiple measurements every 5 minutes. Then line plots of phenotypes for each vital sign were created by plotting the mean value and 95% confidence interval around the mean. The six vital signs include:

- - - Systolic blood pressure
    - Diastolic blood pressure
    - Heart rate
    - Temperature
    - Blood oxygen saturation
    - Respiratory rate

Phenotypes are shown in separate colors. Plots were created by Seaborn lineplot Python package.^4^

1. **Predicting cluster members in new datasets**

In the testing cohort, we used a prospective approach to assign phenotype membership to subject based upon clinical characteristics of typical cluster members in the training cohort.

To accomplish this, we first preprocessed the data using the procedure above (B). We then predicted phenotype assignments by calculating the Euclidean distance from each testing cohort admission to the centroid of each phenotype from training cohort. Consider the *i*th subject with *p* features. We represent it as $X_{i}=[x_{i1}, x_{i2}, \cdots, x_{ip}]$. We denote the mean of the kth phenotype with $\mu_{k}=[\mu_{k1}, \mu_{k2},\cdots,\mu_{kp}]$ and represent it as the center of the phenotype. Thus, we calculate the Euclidean distance of the *i*th admission to the center of the kth phenotype, $d_{i, k}$ as:

$$d_{i, k}= \sqrt{\sum_{j=1}^{p} {(x_{ij-}\mu_{kj})}^{2}}$$

We calculate distances of all admissions to all phenotype centroids and assigned each admission to its nearest phenotype.

1. **Definition of clinical characteristics**

Chronic disease burden was characterized by Charlson-Deyo comorbidity index scores.^5^ Chronic kidney disease was determined from medical histories obtained prospectively at the time of enrollment and from a validated combination of International Classification of Diseases codes from electronic health records^6^. Severity of illness was characterized by SOFA and Modified Early Warning Score (MEWS) based on worst values within first 24 hours of hospital admission.^7^ Missing SOFA and MEWS scores were imputed with 0.

Measurements for clinical biomarkers that fell outside of expert-defined ranges were considered outliers and were removed from the data. All measurements within 24 hours of hospital admission were used to detect highest or lowest value. Ranges of outliers and directionality of worst values are listed in eTable 2. Only results among patients with measurements were reported. We presented continuous variables as mean (SD) and median values with interquartile ranges and as frequencies and percentages for categorical variables.

For blood pressure, invasive measurements were used, and in absence of invasive measurements at a specific date and timestamp, noninvasive measurements were used. Duration of blood pressure below certain cutoff was determined in minutes after forward-propagating previous values. We identified number of pressors and need for inotrope in the first 24 hours of admission based on medications file where dopamine, droxidopa, midodrine, ephpedrine, epinephrine, norepinephrine, phenylephrine, and vasopressin were considered for vasopressors and dobutamine and milrinone for inotrope. Troponin measurements includes Troponin T and Troponin I. In order to determine FiO2 value at each date and time stamp, formulas were used to imputed FiO2 from oxygen delivery device and corresponding oxygen flow rate.^7^ If no oxygen flow rate is given, default FiO2 was imputed based on respiratory device. If oxygen flow rate is outside specified range, minimum and maximum flow rate were used for imputing FiO2. If formula result is greater than maximum per-device FiO2, the maximum FiO2 was imputed. In absence of PaO2 to calculate PaO2/FiO2 ratio, SpO2/FiO2 to PaO2/FiO2 conversion was used.^7,8^

To determine reference creatinine, we used previously validated modification of the NHS England alert algorithm.^9^ For patients with available preadmission measurements, reference value was defined as either the lowest in the last 7 days or a median of values from the preceding 8 to 365 days depending on availability of previous results. For patients with no available preadmission measurements and no history of chronic kidney disease (CKD), we used the lowest of admission creatinine and estimated baseline creatinine using the Modification of Diet in Renal Disease Study equation assuming that baseline estimated glomerular filtration rate (eGFR) is 75 ml/min per 1.73 m2. For patients with known history of CKD and no available preadmission measurements we used lowest creatinine value on admission day. After first seven days of hospitalization, minimum serum creatinine measurements in preceding 7 days was used as the reference creatinine. Reference creatinine was used to estimate preadmission reference glomerular filtration rate using Chronic Kidney Disease Epidemiology Collaboration equation.^10^ Chronic kidney disease was determined from medical histories obtained prospectively at the time of enrollment and from a validated combination of International Classification of Diseases codes from electronic health records.^10^ Chronic kidney disease stages were determined based on reference eGFR according to guidelines.^11,12^

**Diagnosis codes**

We determined category of admission diagnosis codes, which are assigned either as International Classification of Diseases, 9th Revision, Clinical Modification (ICD-9-CM) or International Classification of Diseases, 10th Revision, Clinical Modification (ICD-10-CM) code. We used general equivalence mappings to assist with the conversion ICD-10-CM codes to ICD-9-CM codes.^13^ The Clinical Classification Software (CCS)^11^ consists of two related classification systems, single-level and multi-level, which are designed to meet different needs. We used multi-level CCS which expands the single-level CCS into a hierarchical system and enables evaluating larger aggregations of conditions and procedures or exploring them in greater detail. The multi-level system has four levels for diagnoses and three levels for procedures, which provide the opportunity to examine general groupings or to assess very specific conditions and procedures. We showed distribution of most common Level 1 and Level 2 codes for each cluster as well as distribution of all admission diagnosis codes that are present in at least 1% proportion of patients.

1. **Definition of clinical outcomes**

We determined complications occurring anytime during hospitalization, including infectious and mechanical wound complications (wound complications), acute kidney injury (AKI), mechanical ventilation (MV) and intensive care unit (ICU) admission for greater than 48 hours, cardiovascular (CV) complications, neurological complications and/or delirium, sepsis, and venous thromboembolism (VTE). We used the exact dates and times to calculate the duration of MV, ICU, and hospital stay. In order to determine the duration of invasive mechanical ventilation, we developed an algorithm to identify the start and stop times for ventilation based on flowsheet data. Patient was determined to be on mechanical ventilation at a time point if the respiratory device is recorded as ventilator or endotracheal tube (ETT) or there is a recorded measurement value for tidal volume, end-tidal carbon dioxide (etCO2), positive end-expiratory pressure (PEEP), mechanical respiratory rate, or ventilator mode. We identified need for pressors or inotropes (dobutamine, dopamine, droxidopa, midodrine, milrinone, ephpedrine, epinephrine, norepinephrine, phenylephrine, or vasopressin) during hospitalization based on detailed medication records data as binary variable. Acute kidney injury (AKI) was determined using available clinical information according to Kidney Disease: Improving Global Outcomes criteria (0.3 mg/dl increase in serum creatinine within 48 hours or 50% increase from baseline within seven days or decrease in urine output to less than 0.5 ml/kg/hr for six hours).^11^ Community-acquired AKI was defined as development of AKI within 24 hours of hospital admission. Delirium was defined as at least one positive Confusion Assessment Method (CAM) score or having ICD-9 or ICD-10 codes for delirium. The International Classification of Diseases, Ninth and Tenth Revision, Clinical Modification (ICD-9-CM, ICD-10-CM) were used to the remaining complications.^14-18^ Date of death was determined using hospital records and the Social Security Death Index database was used to confirm death dates and obtain death dates for subjects who were not in hospital records. Thirty-day and three-year mortality were defined if the death date is thirty days or three year from the discharge date of the index hospital admission.

# eFigure 1. Cohort selection and exclusion criteria


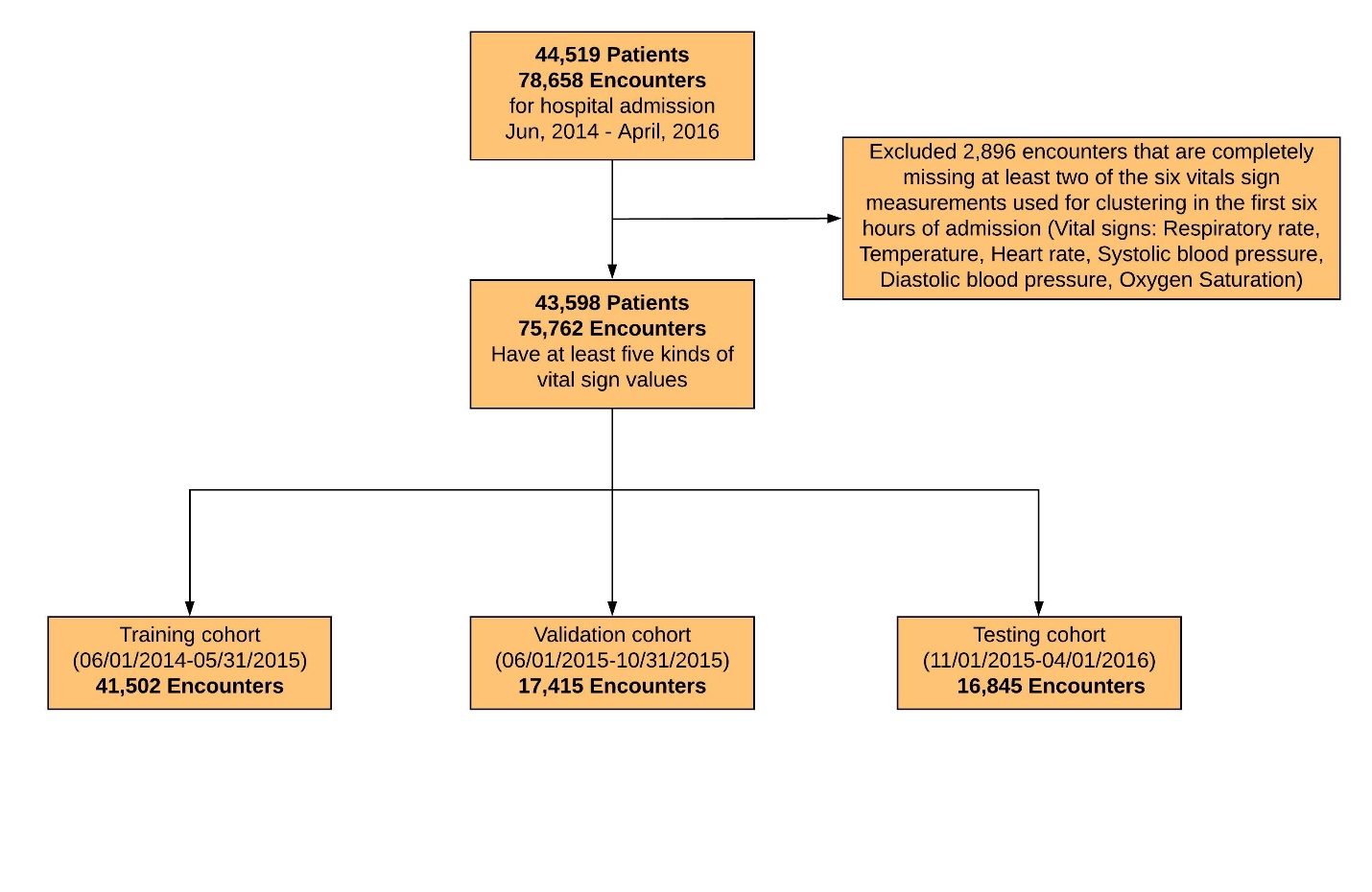


# eFigure 2. Spearman correlation heat map for the training cohort (N=41,502)


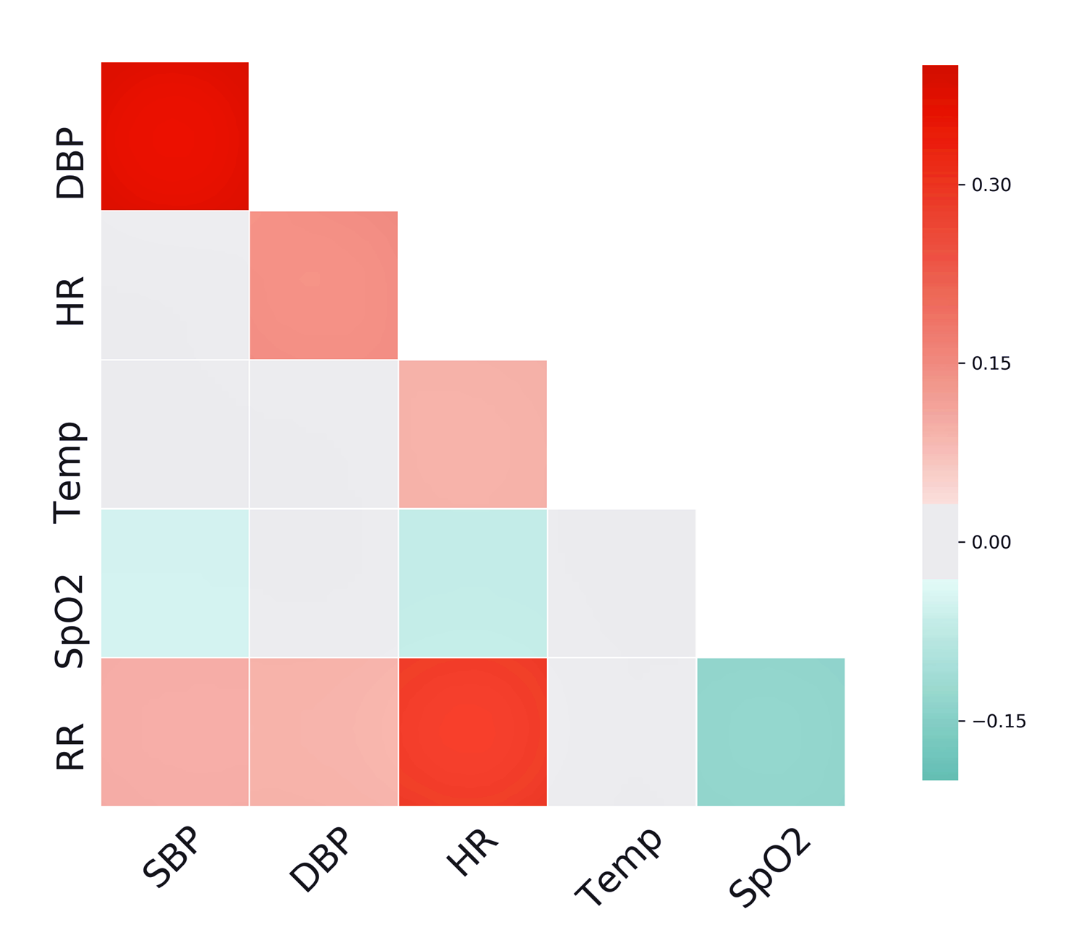


Spearman correlation heat map shows the pairwise spearman rank order correlation coefficient among the 6 vital signs studied in our paper. The darker red color, the higher correlation in positive direction.

Abbreviations: RR: respiratory rate; SpO2: peripheral capillary oxygen saturation; Temp: temperature; HR: heart rate; SBP: systolic blood pressure; DBP: diastolic blood pressure.

# eFigure 3. Deep temporal interpolation and clustering network algorithm


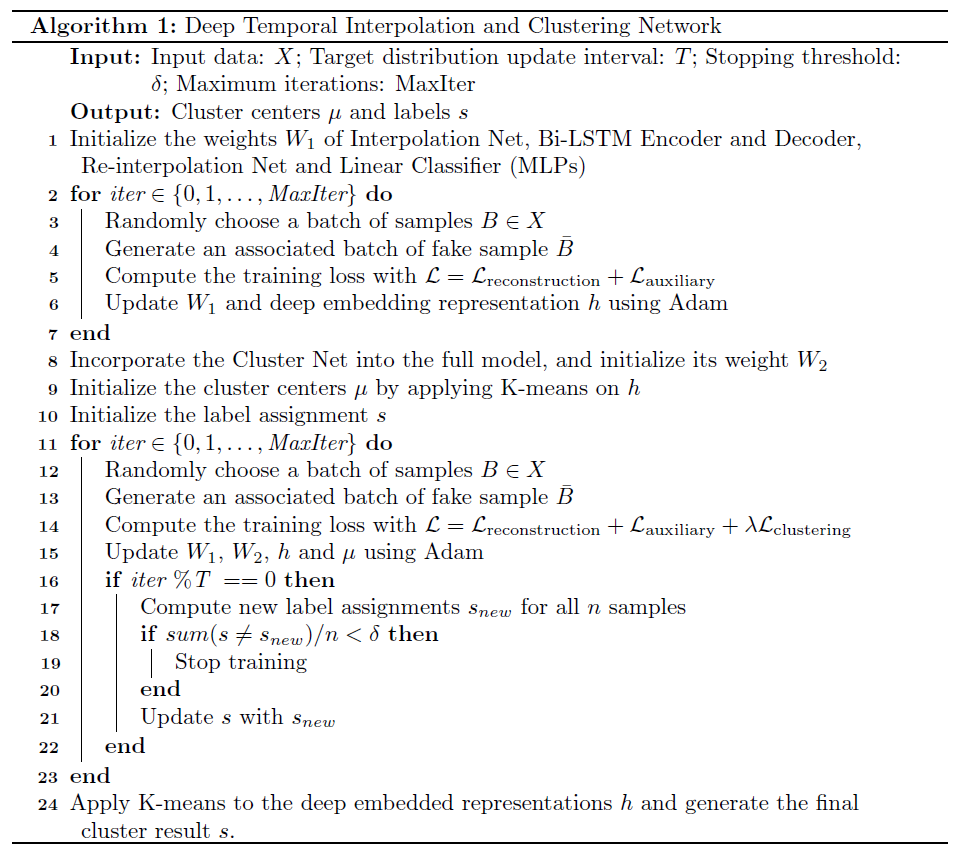


# eFigure 4. Gap statistic and elbow approaches showing the optimal number of clusters


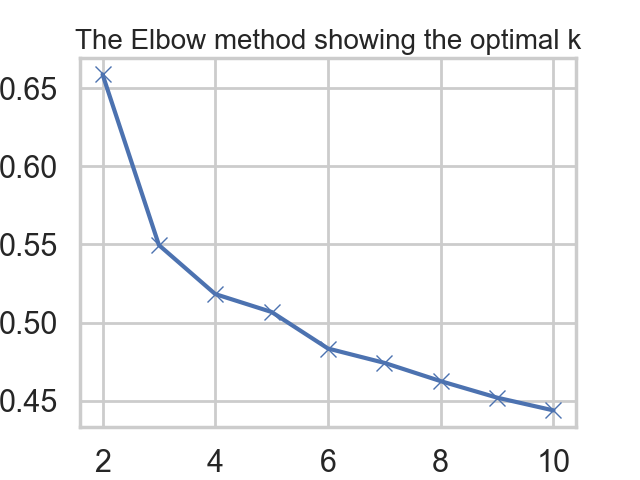
 (**A)**.


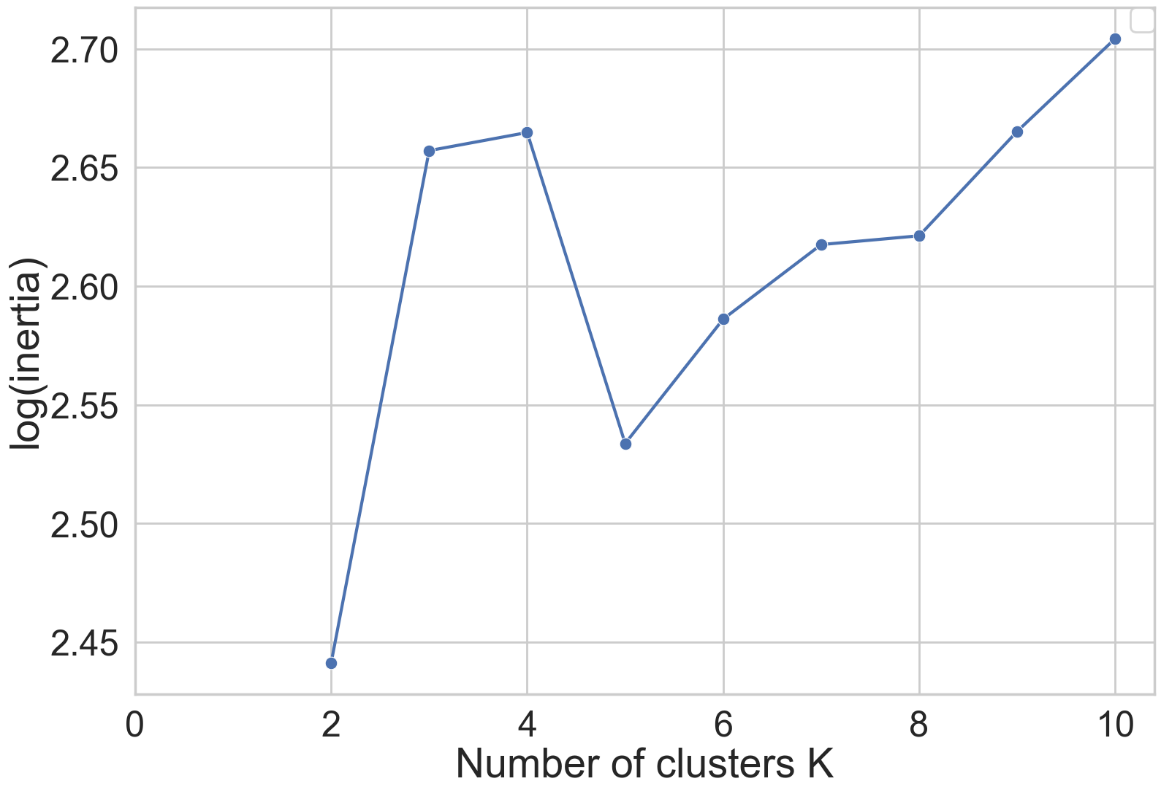


**(B)**.

(A) Elbow approach shows the optimal number of clusters. The value of k at the “elbow”, the point after which the distortion starts decreasing in a linear fashion, suggests the optimal number of clusters. (B) Gap statistic approach shows the optimal number of clusters. Higher gap statistic value suggests the optimal number of clusters.

# eFigure 5. Alluvial plot showing distribution of phenotypes across worst SOFA scores of patients within first 24 hours of admission in the training cohort


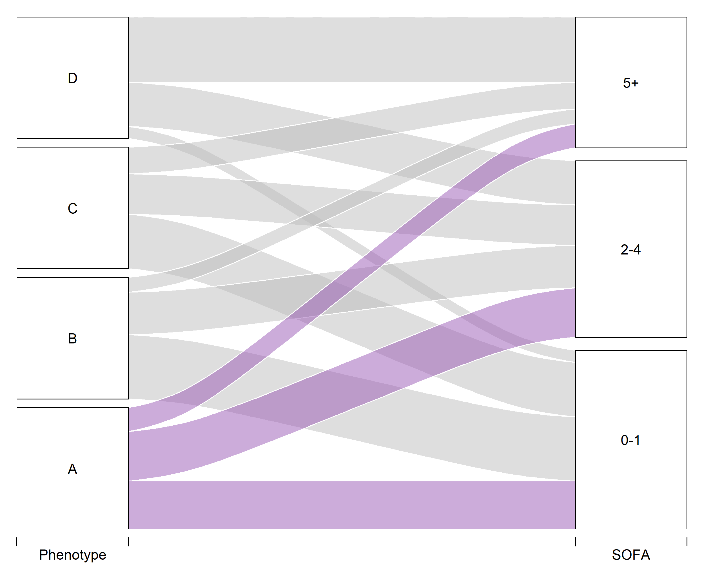

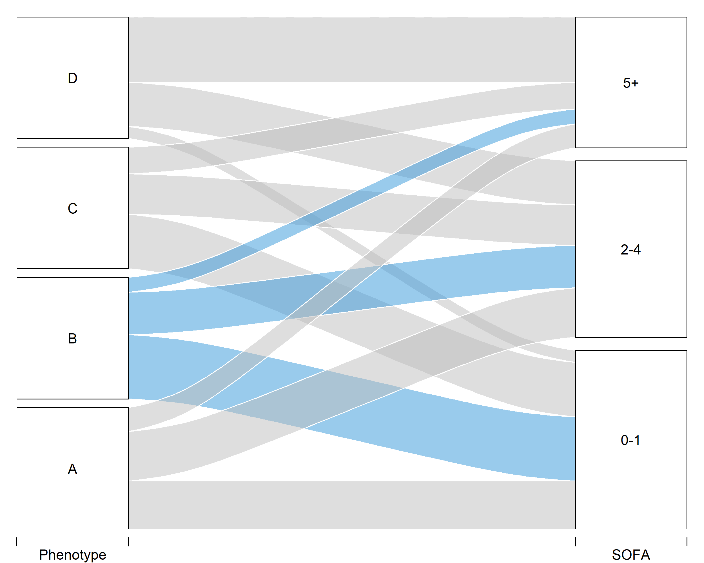


(A) Phenotype A (B) Phenotype B


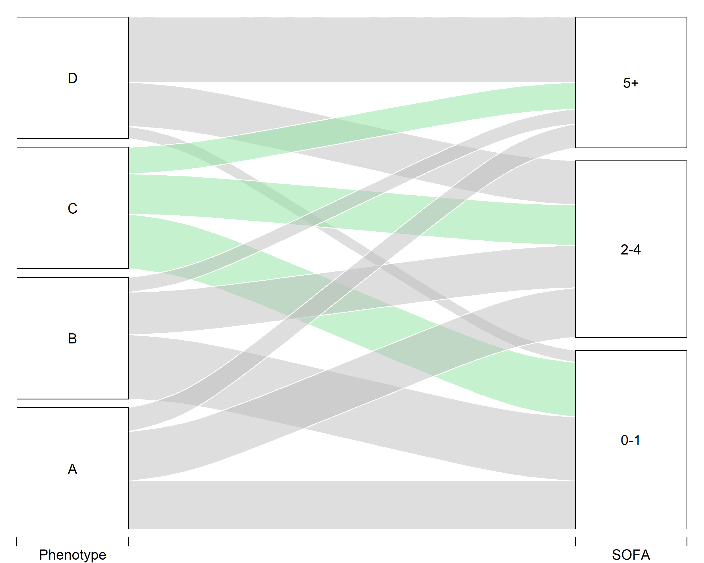

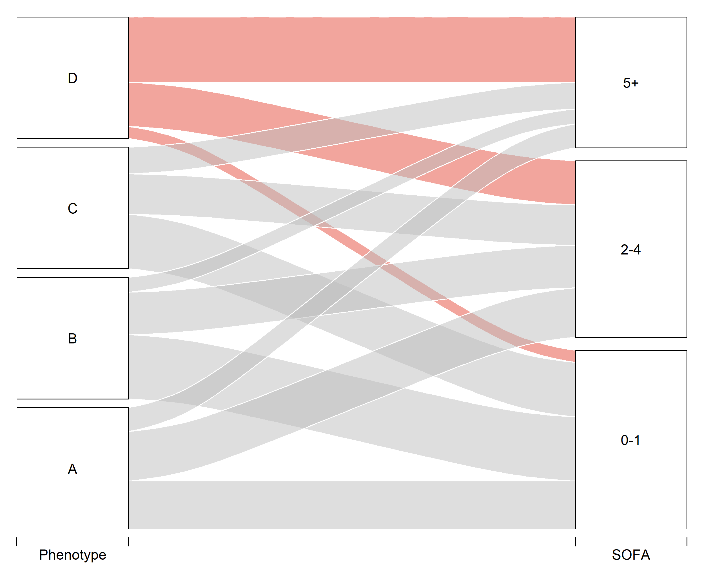


(C) Phenotype C (D) Phenotype D

For each phenotype, the larger percentage of patients with that score, the broader the ribbon.

# eFigure 6. Chord diagrams showing the distribution of patients with higher SOFA scores (i.e., 2+) within first 24 hours of admission of six organ systems by phenotypes in the training cohort


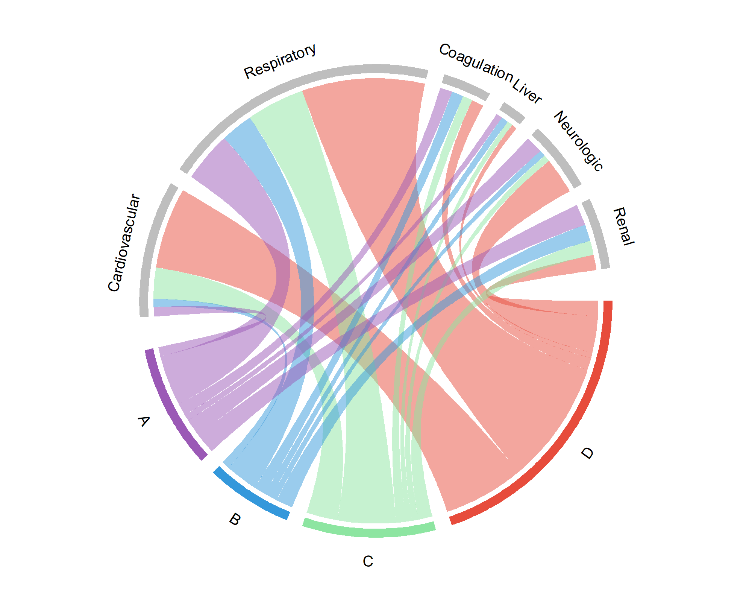


(i) All phenotypes


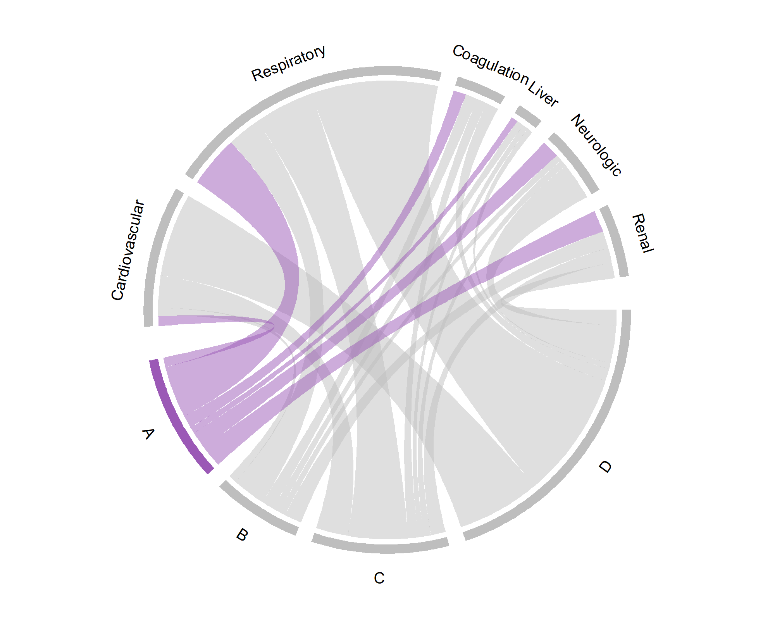


(ii) Phenotype A


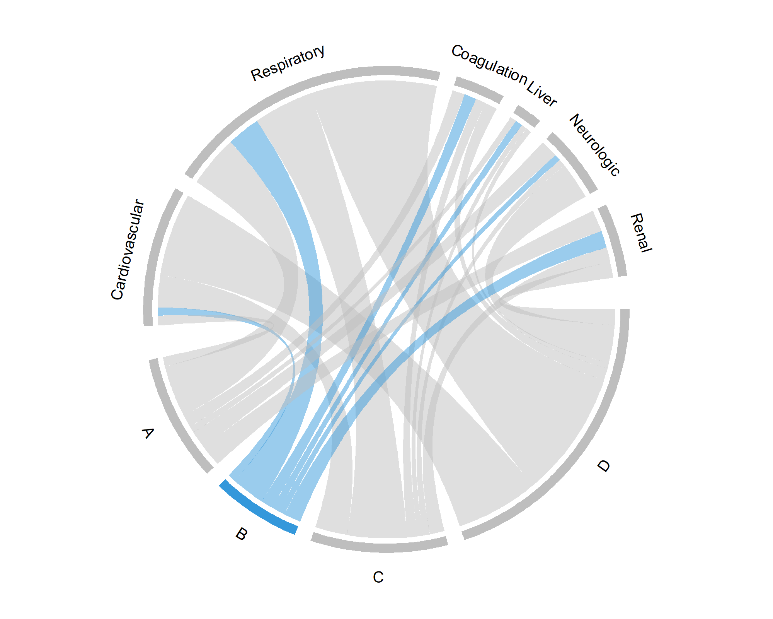


(iii) Phenotype B


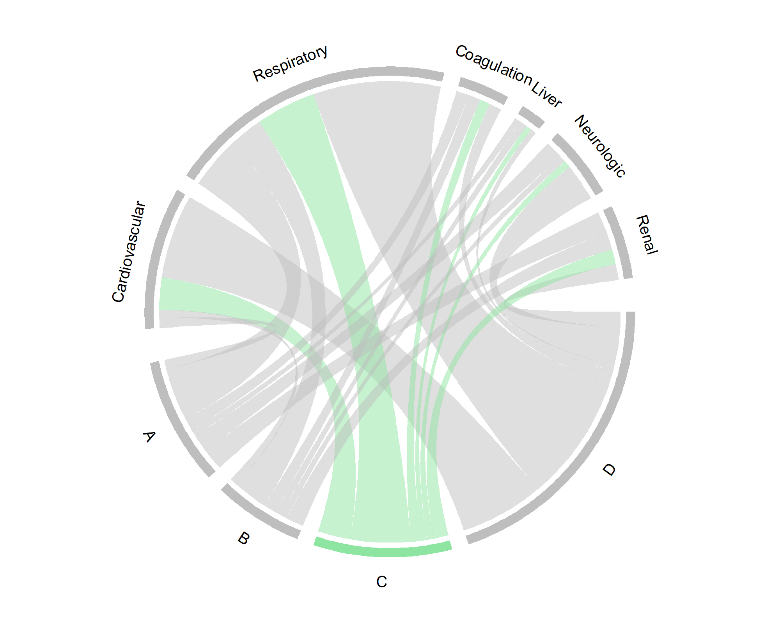


(iv) Phenotype C


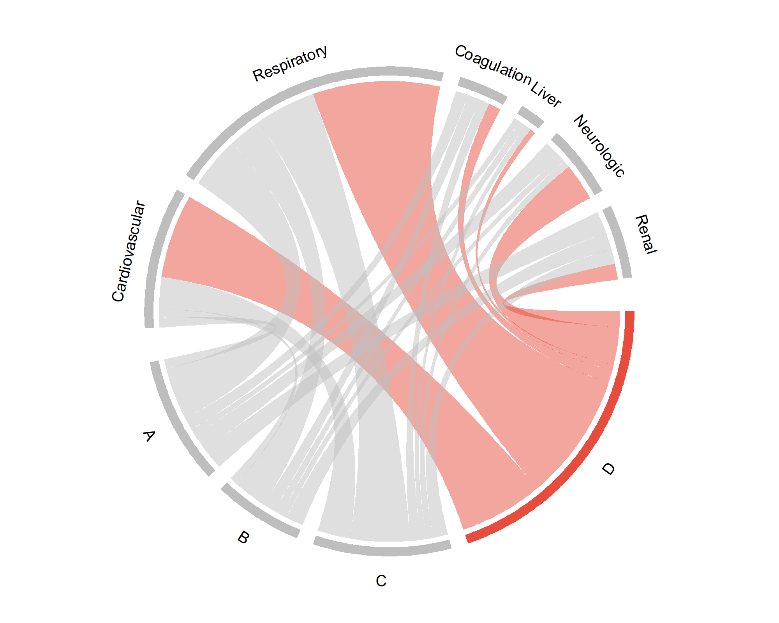


(v) Phenotype D

For each phenotype, the larger percentage of patients with higher score of that organ system, the border the ribbon.

# eFigure 7. Chord diagrams showing the distribution of nine most common admission diagnosis groups by phenotype in the training cohort

(i) All phenotypes


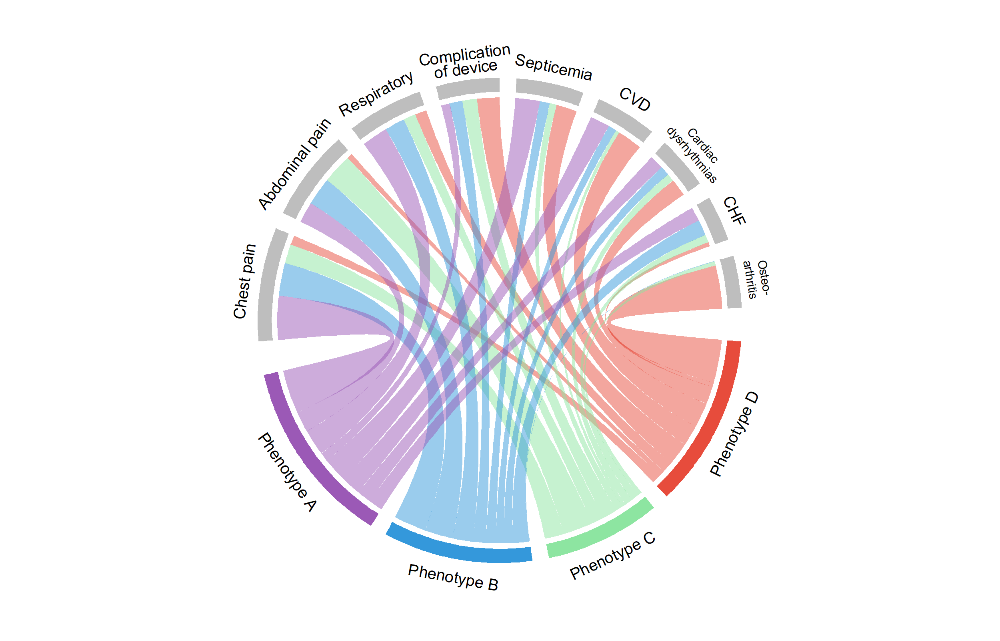


(ii) Phenotype A


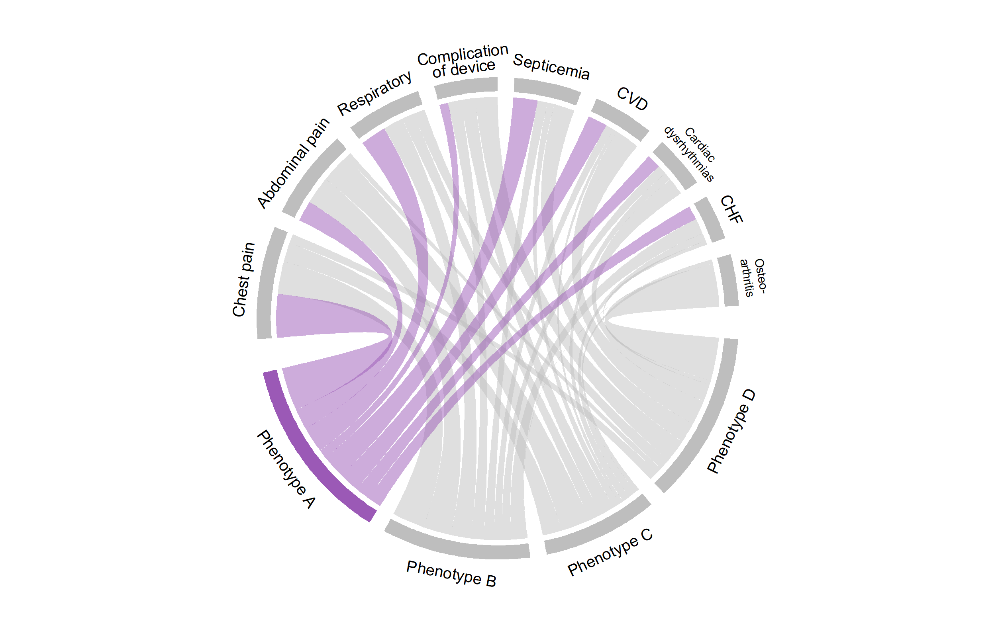


(iii) Phenotype B


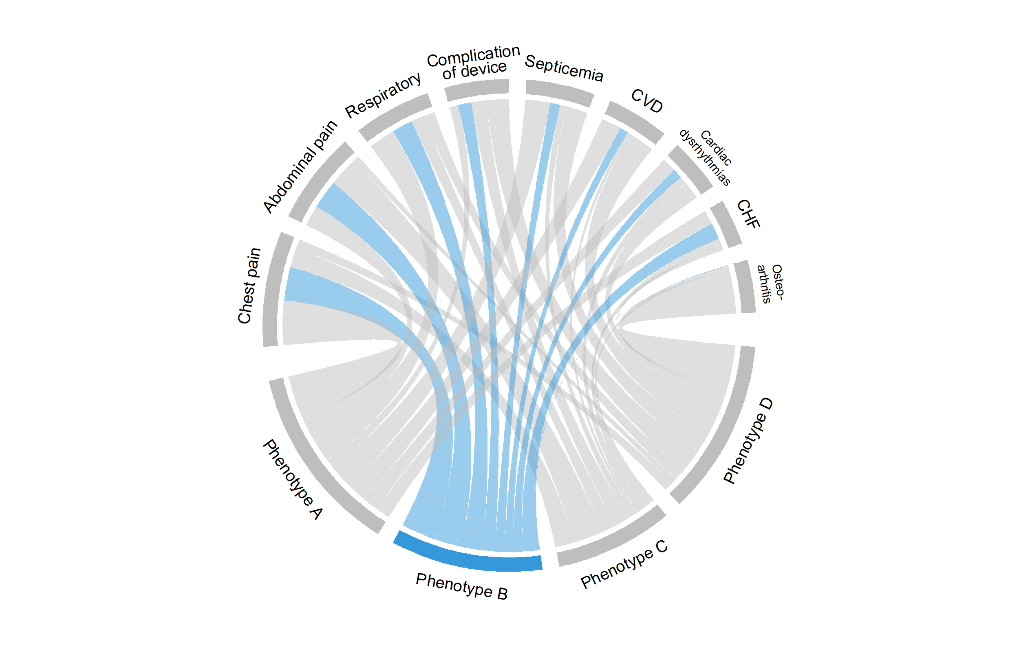


(iv) Phenotype C


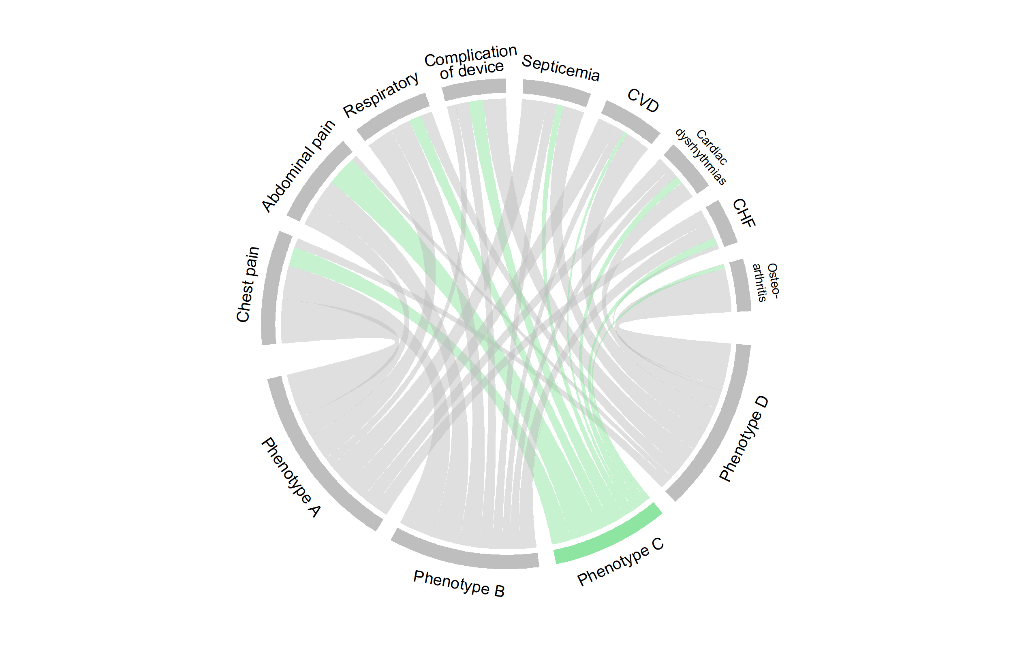


(v) Phenotype D


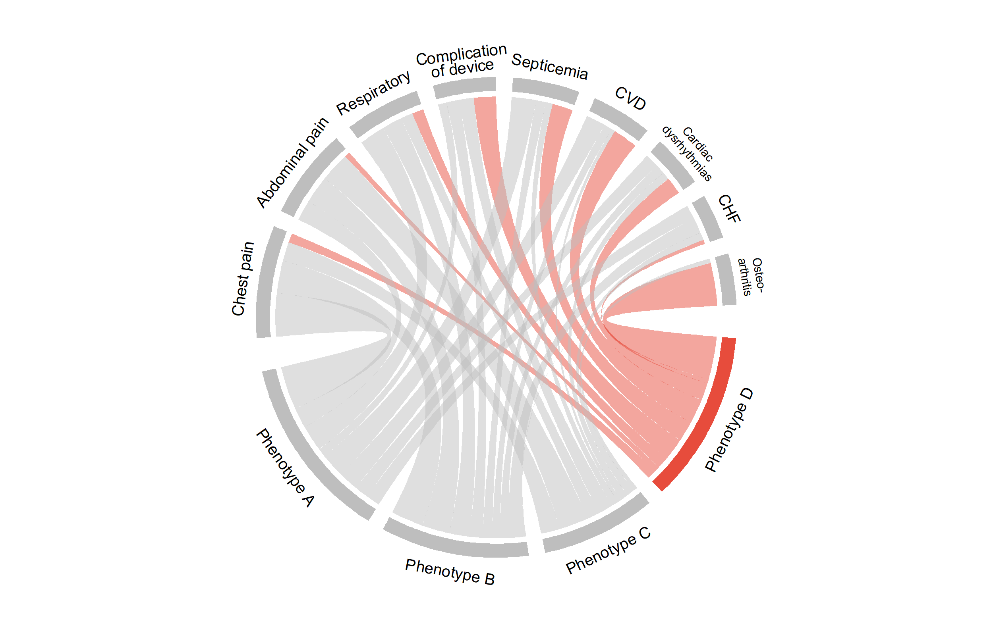


Diagnosis groups are shown in order of frequencies of all patients. For each phenotype, the larger percentage of patients with that diagnosis, the border the ribbon. Detailed diagnosis groups from left to right are: Nonspecific chest pain, Abdominal pain, Other and unspecific lower respiratory disease, Complication of device; implant or graft, Septicemia (except in labor), Acute cerebrovascular disease, Cardiac dysrhythmias, Congestive heart failure; nonhypertensive, and Osteoarthritis.

# eFigure 8. Survival curves and Cox proportional hazards modeling by phenotypes in the training cohort


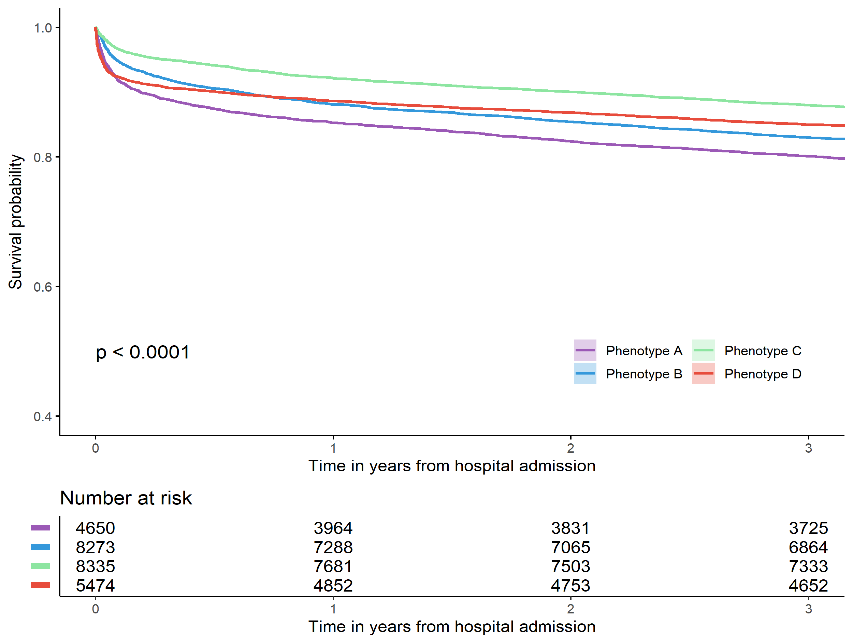


(A)


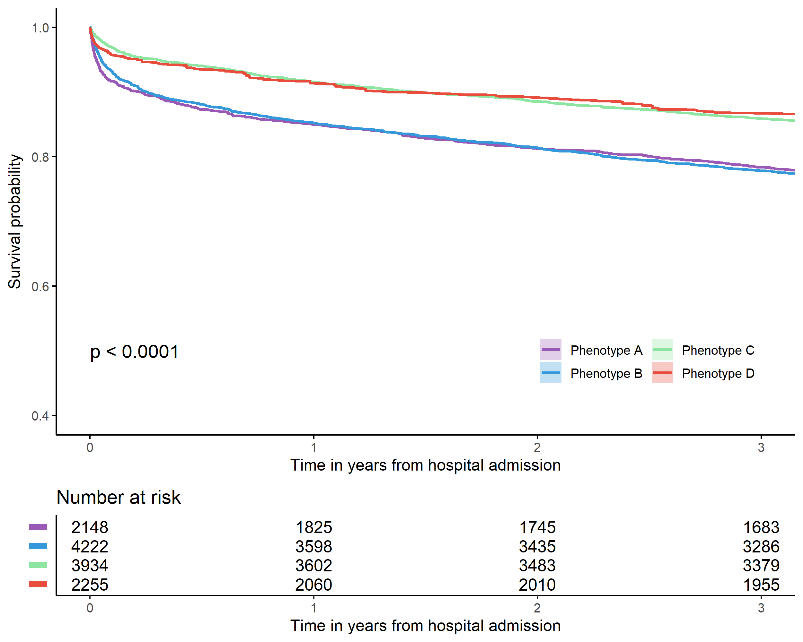


(C)


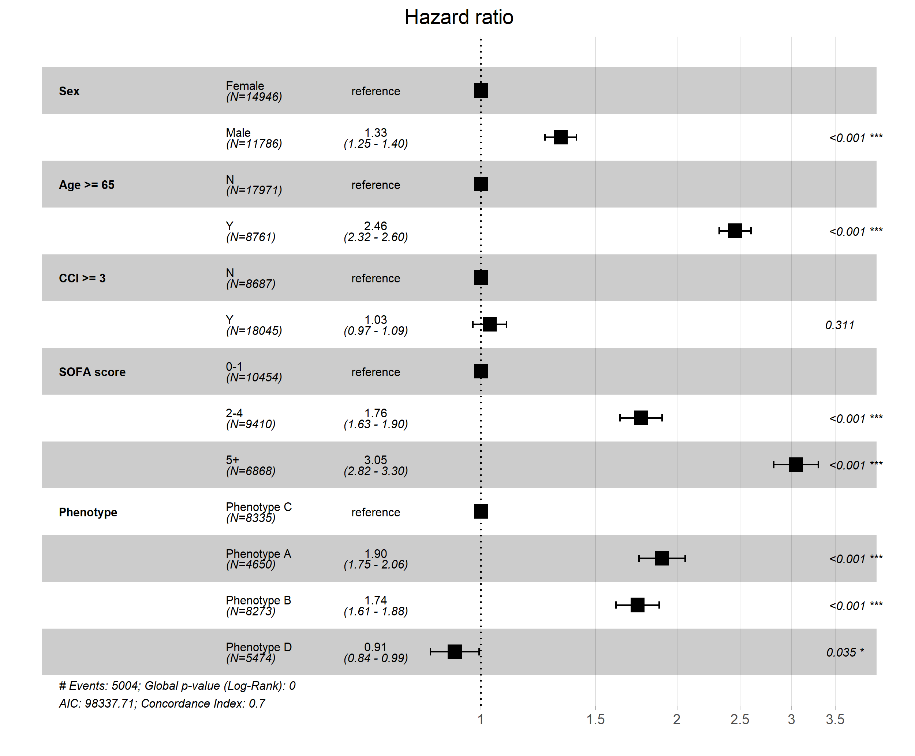


(D)


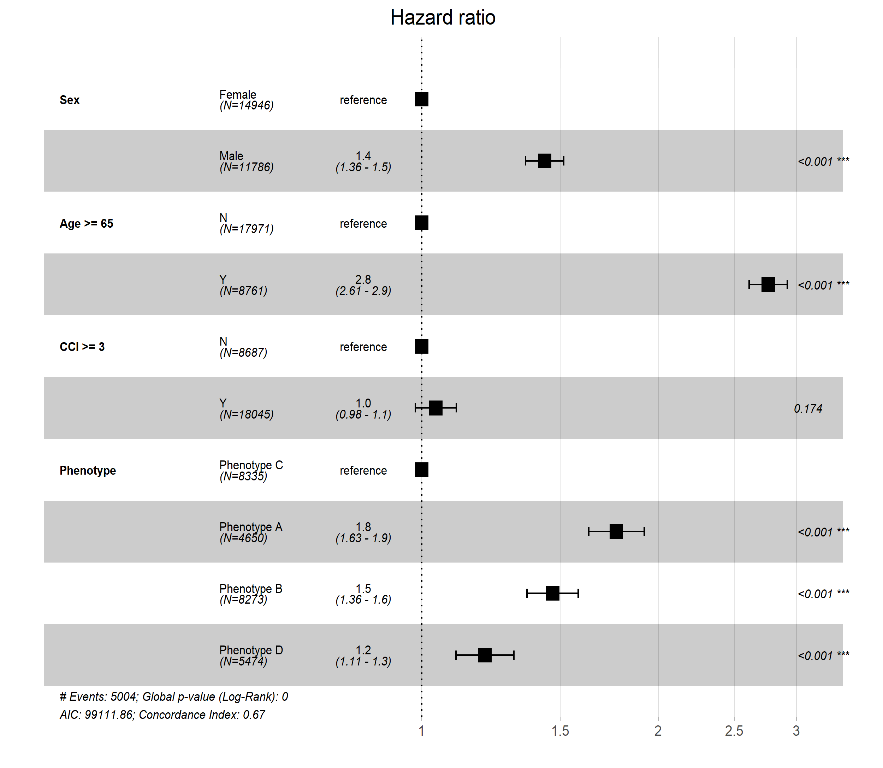


(B)

(A) Phenotype survival curves adjusted using demographic information and comorbidities. (B) Adjusted Cox proportional hazards models using demographic information and comorbidities. (C) Phenotype survival curves adjusted using demographic information, comorbidities, and SOFA scores. (D) Adjusted Cox proportional hazards model using demographic information, comorbidities, and SOFA scores. Abbreviation: CCI: Charlson comorbidity index; SOFA: sequential organ failure assessment.

# eFigure 9. Survival curves and Cox proportional hazards modeling by sepsis patients in the training cohort


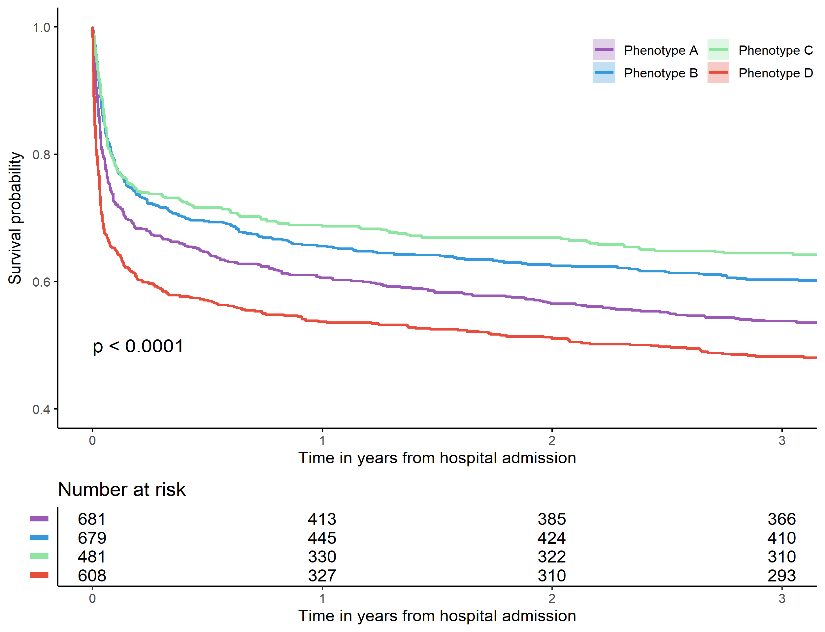


(A)


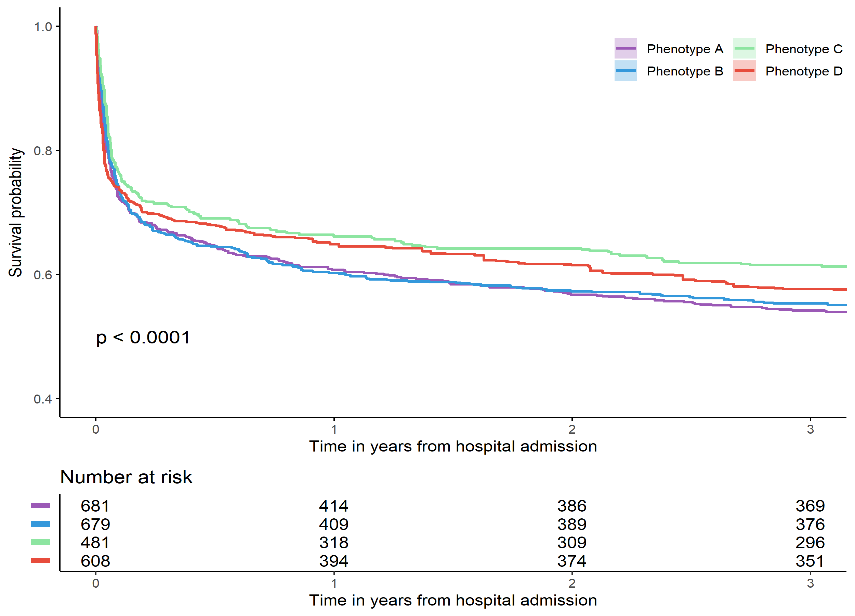


(C)


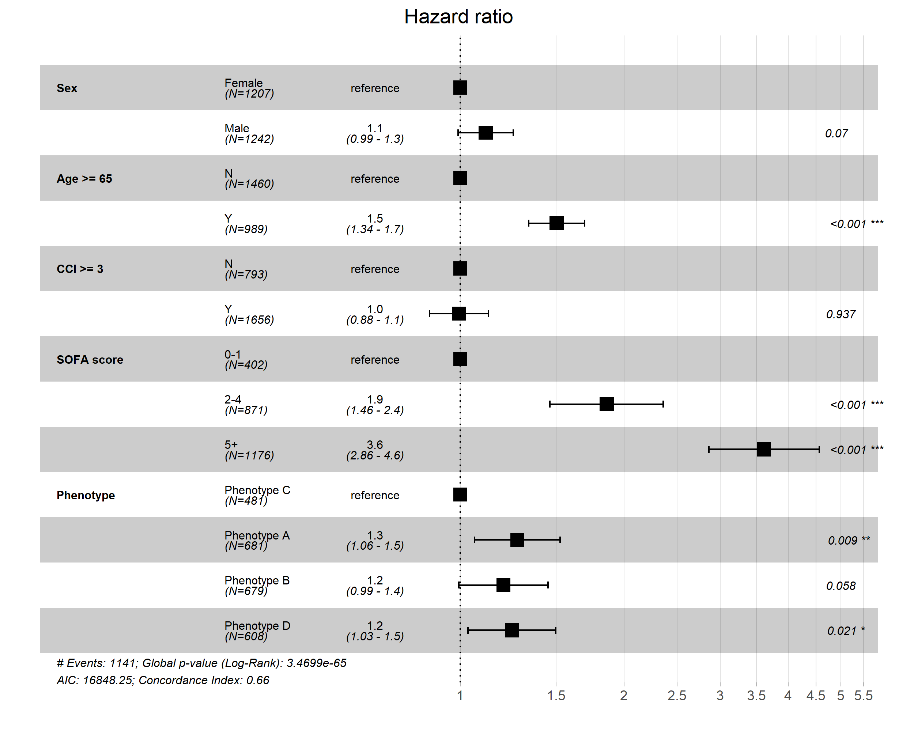


(D)


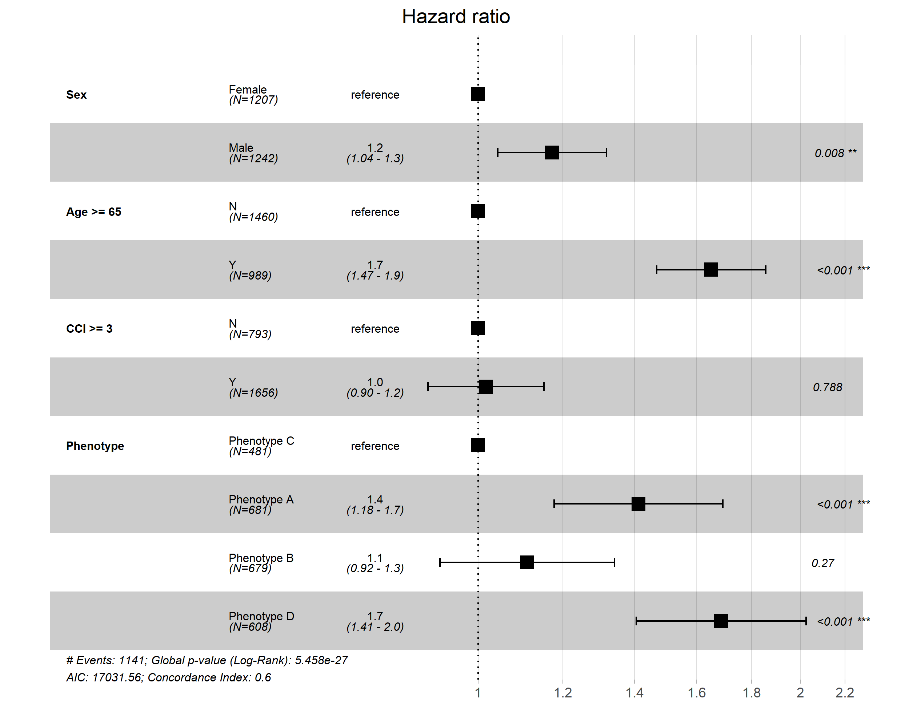


(B)

(A) Phenotype survival curves adjusted using demographic information and comorbidities. (B) Adjusted Cox proportional hazards models using demographic information and comorbidities. (C) Phenotype survival curves adjusted using demographic information, comorbidities, and SOFA scores. (D) Adjusted Cox proportional hazards model using demographic information, comorbidities, and SOFA scores. Abbreviation: CCI: Charlson comorbidity index; SOFA: sequential organ failure assessment.

# eFigure 10. Survival curves and Cox proportional hazards modeling by AKI patients in training cohort


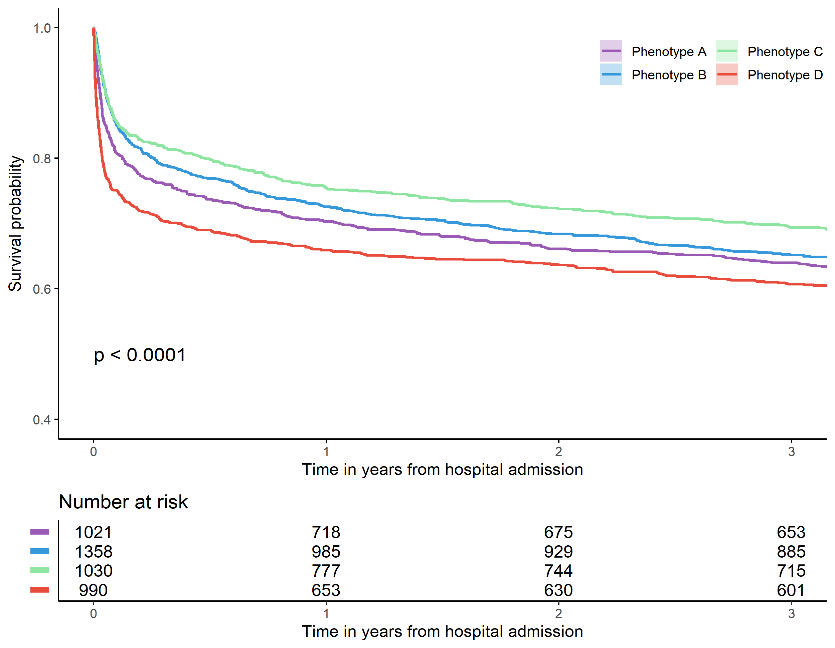


(A)


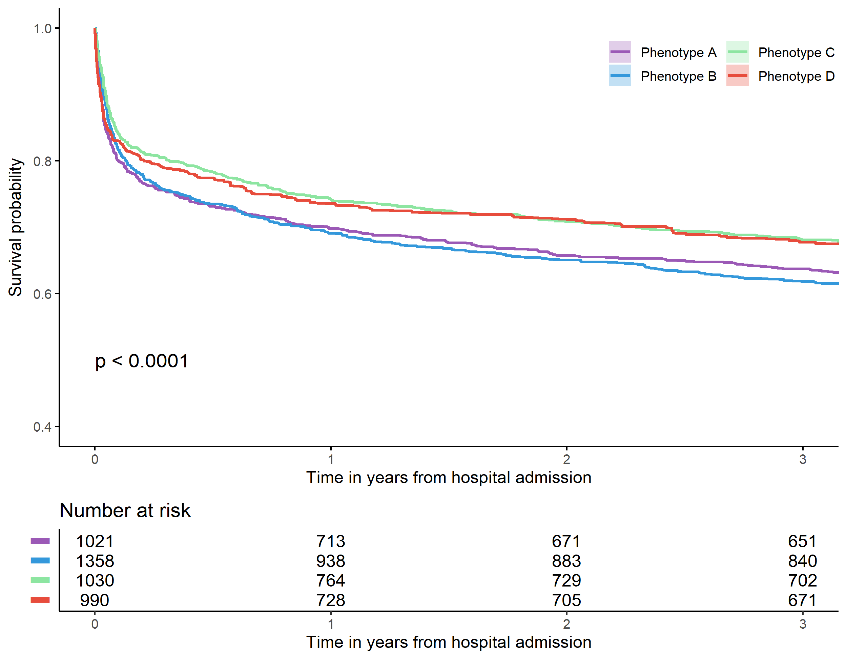


(C)


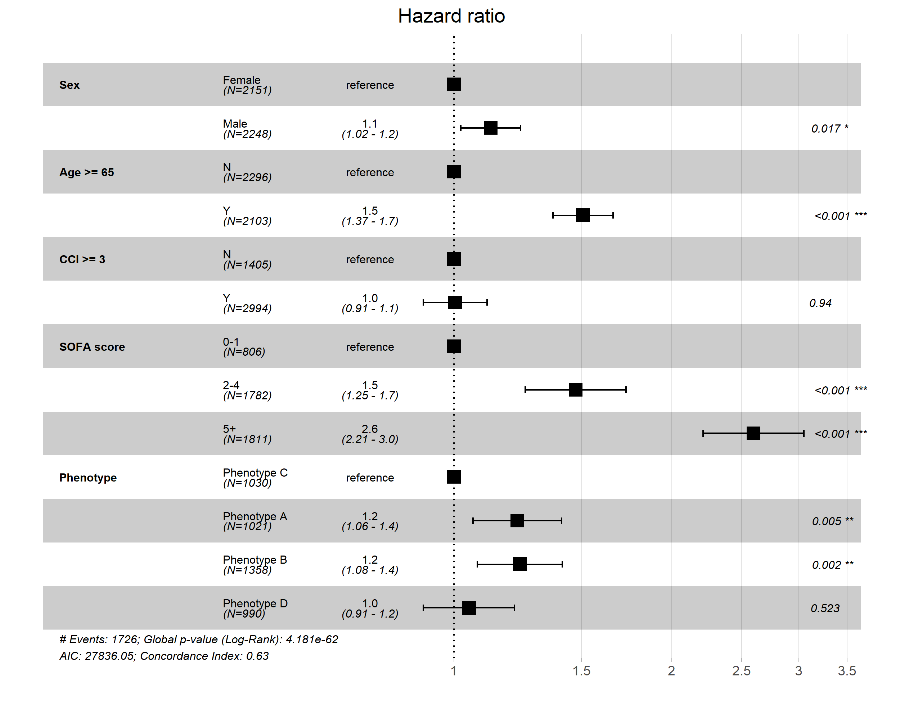


(D)


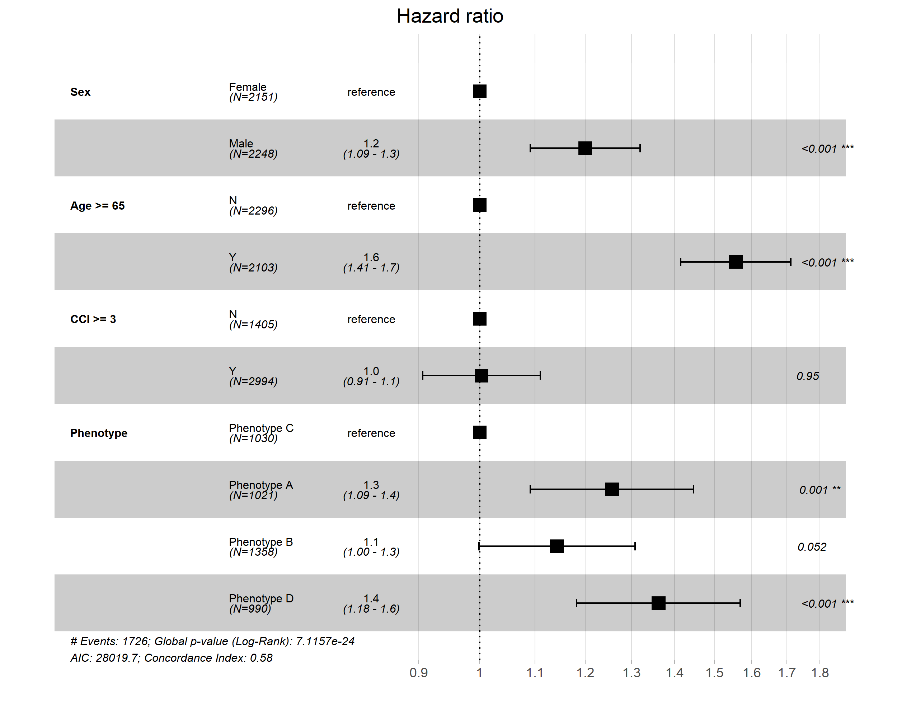


(B)

(A) Phenotype survival curves adjusted using demographic information and comorbidities. (B) Adjusted Cox proportional hazards models using demographic information and comorbidities. (C) Phenotype survival curves adjusted using demographic information, comorbidities, and SOFA scores. (D) Adjusted Cox proportional hazards model using demographic information, comorbidities, and SOFA scores. Abbreviation: CCI: Charlson comorbidity index; SOFA: sequential organ failure assessment.

# eFigure 11. Survival curves and Cox proportional hazards modeling by surgical patients in training cohort


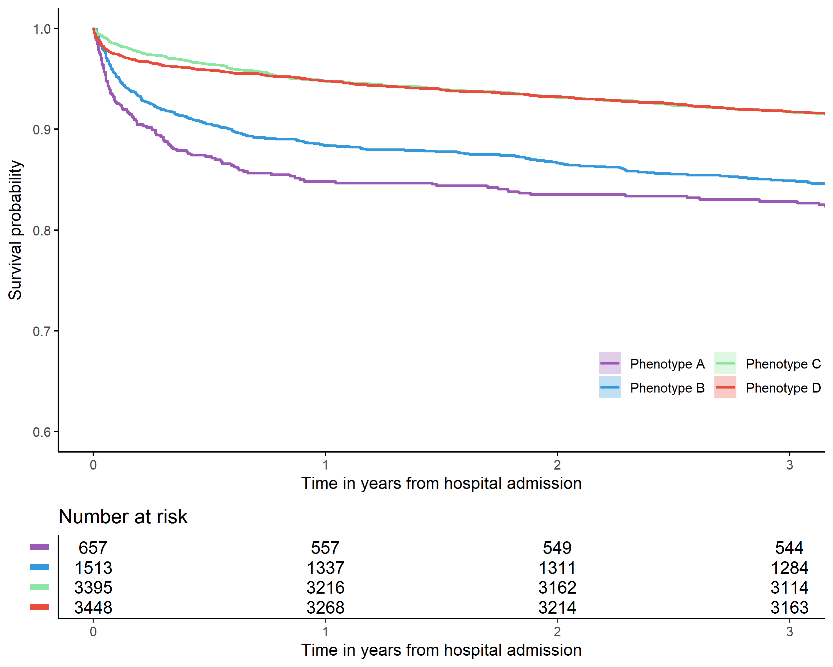


(A)


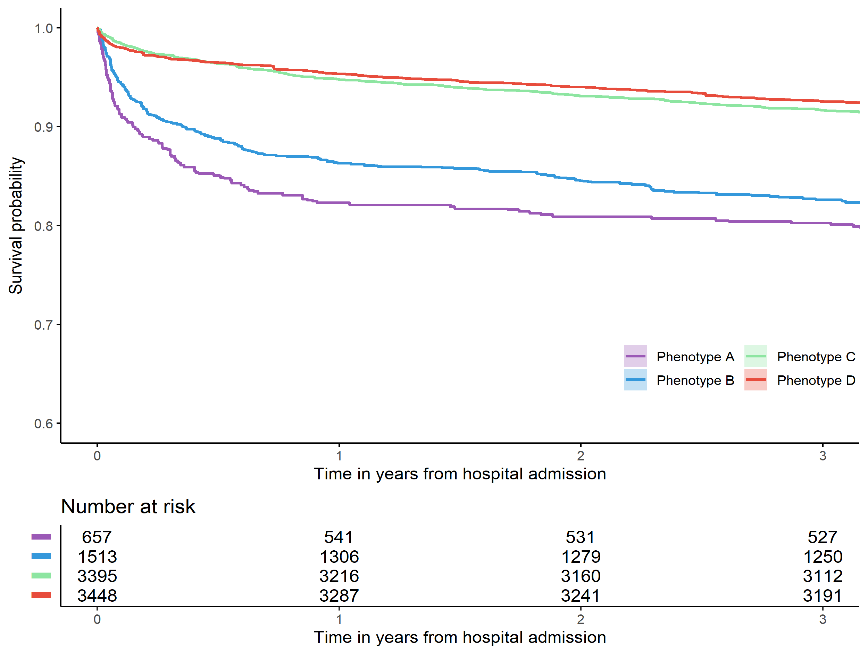


(C)


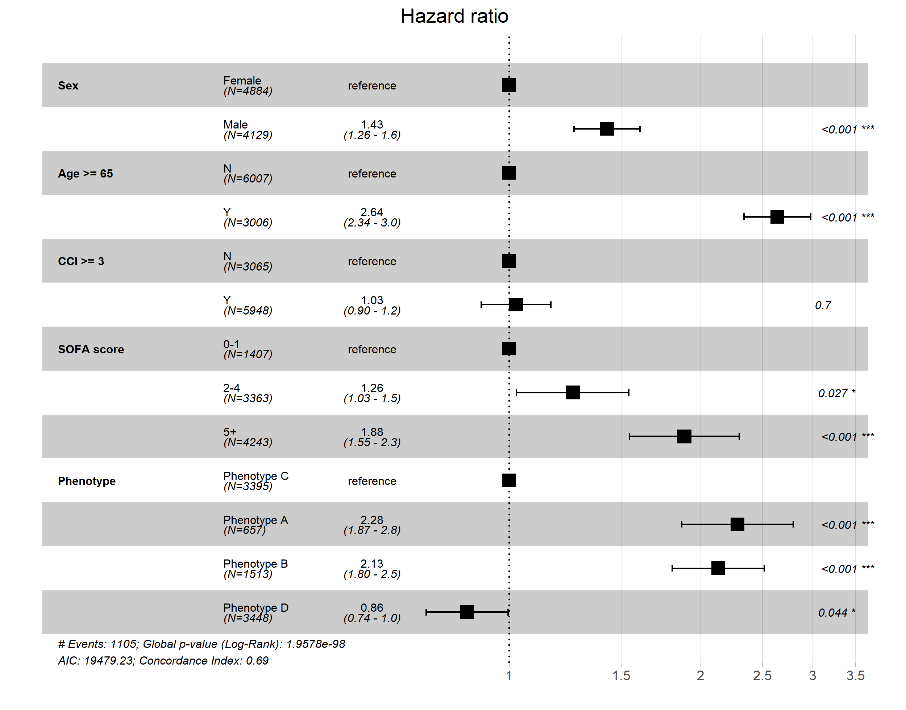


(D)


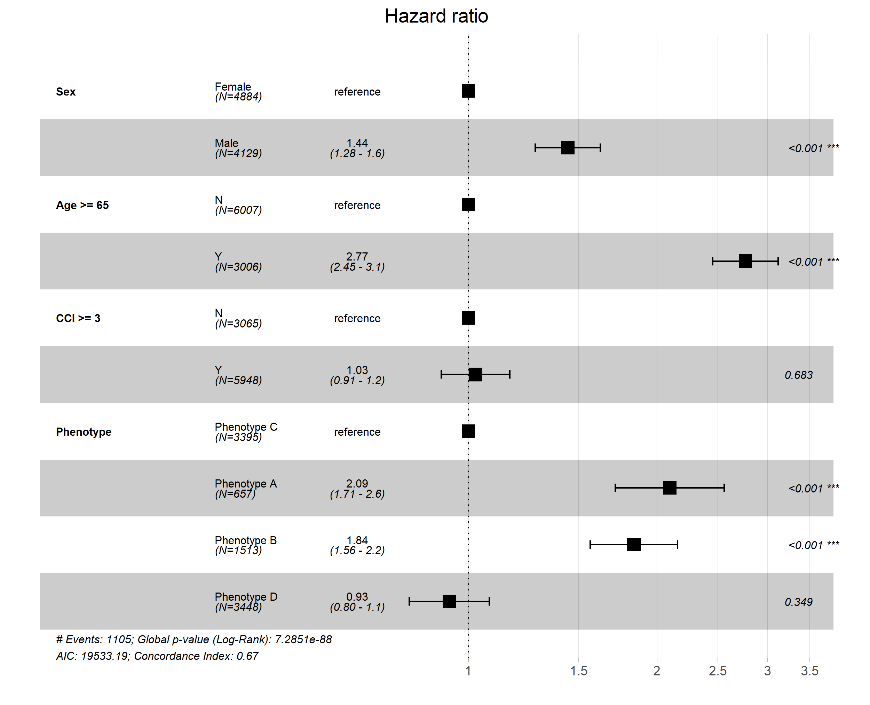


(B)

(A) Phenotype survival curves adjusted using demographic information and comorbidities. (B) Adjusted Cox proportional hazards models using demographic information and comorbidities. (C) Phenotype survival curves adjusted using demographic information, comorbidities, and SOFA scores. (D) Adjusted Cox proportional hazards model using demographic information, comorbidities, and SOFA scores. Abbreviation: CCI: Charlson comorbidity index; SOFA: sequential organ failure assessment.

# eFigure 12. Distribution of vital signs during the first six hours of hospital admission in the testing cohort


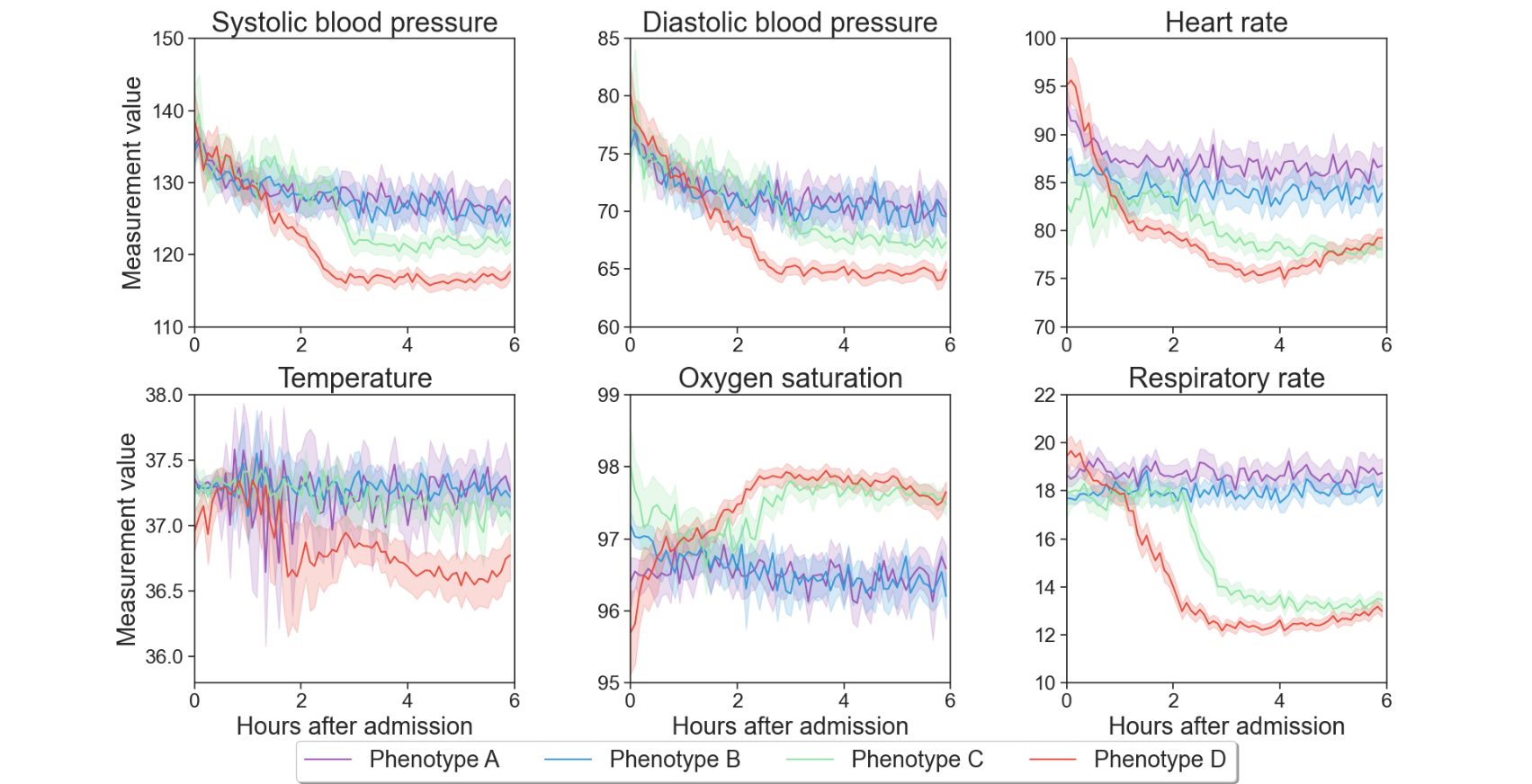


# eFigure 13. t-SNE plot of phenotype assignments in the testing cohort


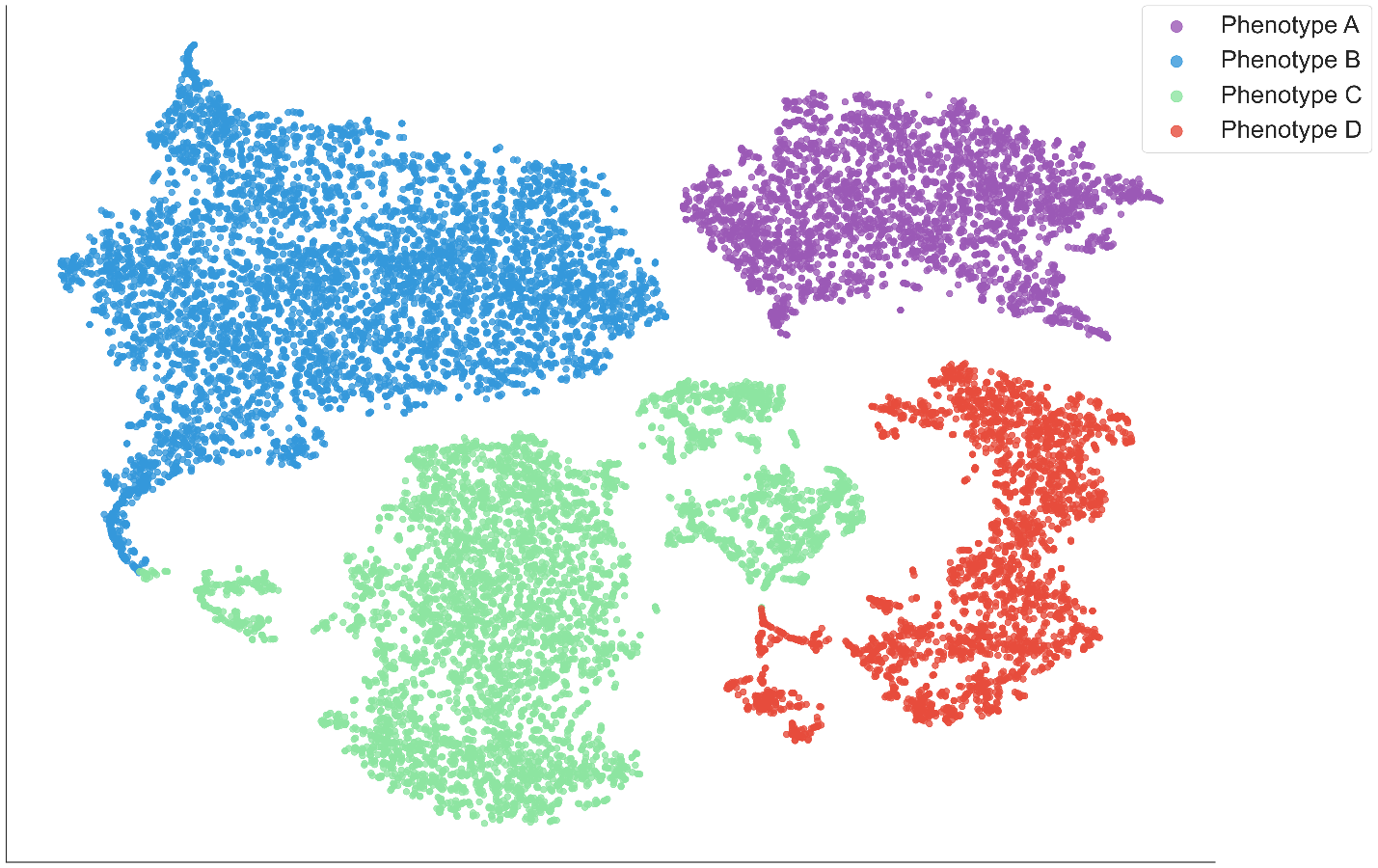


Starting from the original 128 dimensional vital sign representations, we run the t-SNE to reduce to 2 dimensions. Each dot represents a patient. Phenotypes are show in separate colors.

# eFigure 14. Alluvial plot showing distribution of phenotypes across worst SOFA scores of patients within first 24 hours of admission in the testing cohort


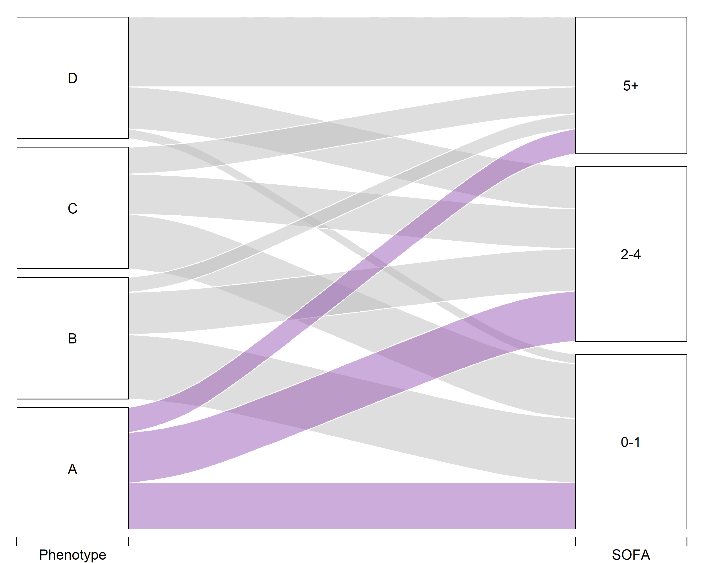

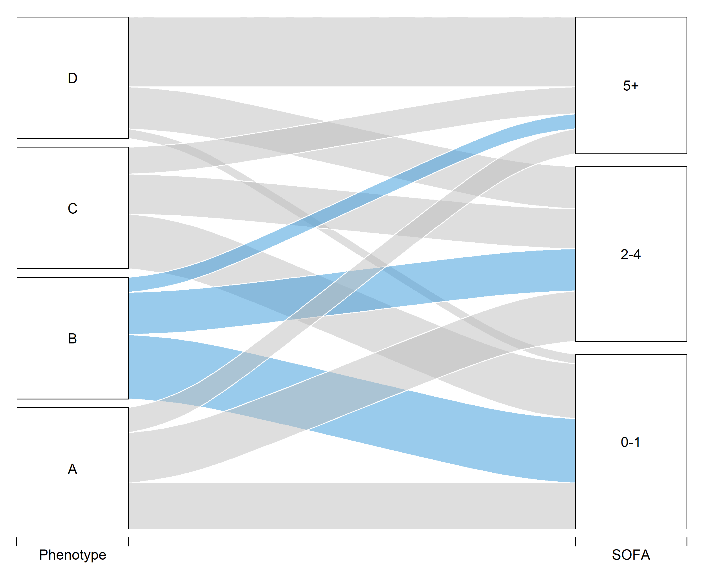


(A) Phenotype A (B) Phenotype B


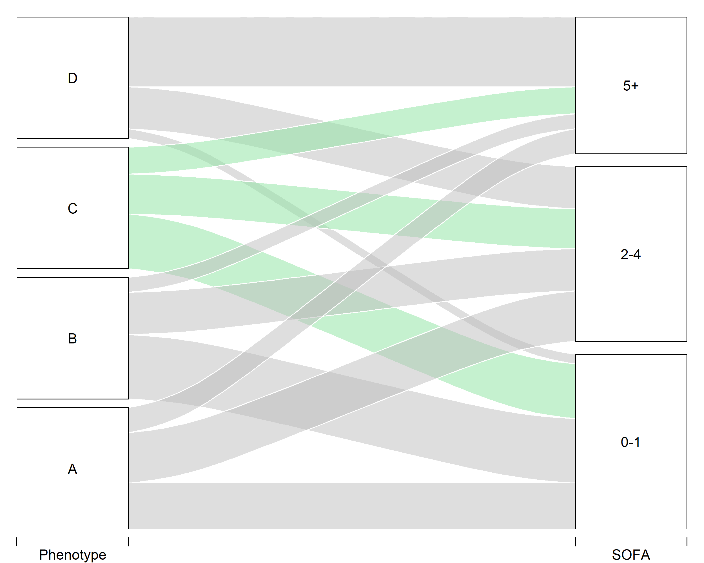

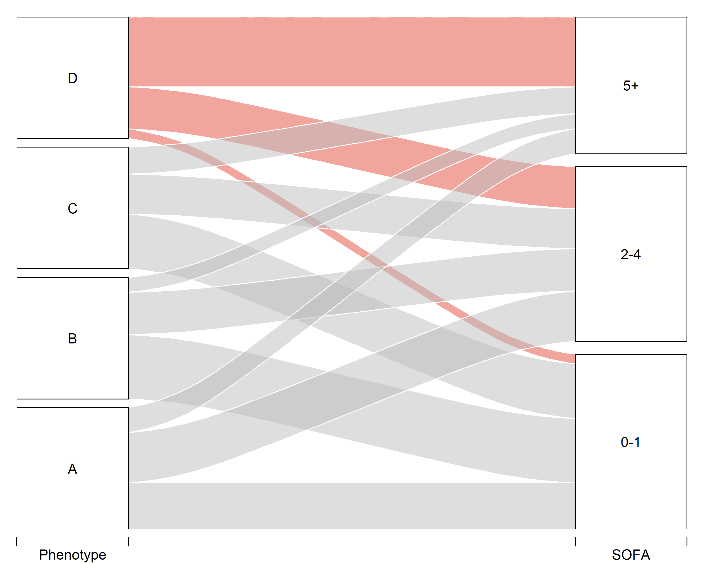


(C) Phenotype C (D) Phenotype D

For each phenotype, the larger percentage of patients with that score, the broader the ribbon.

# eFigure 15. Chord diagrams showing the distribution of patients with higher SOFA scores (i.e., 2+) within first 24 hours of admission of six organ systems by phenotypes in the testing cohort


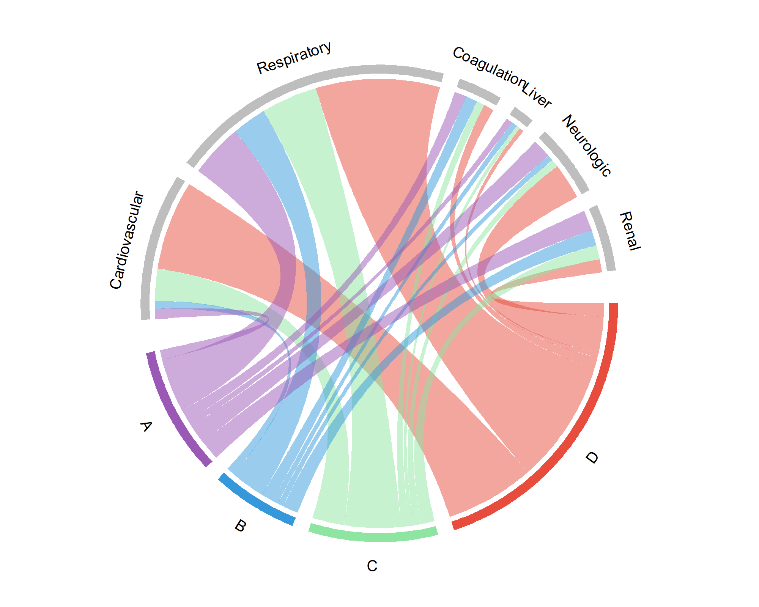


(i) All phenotypes


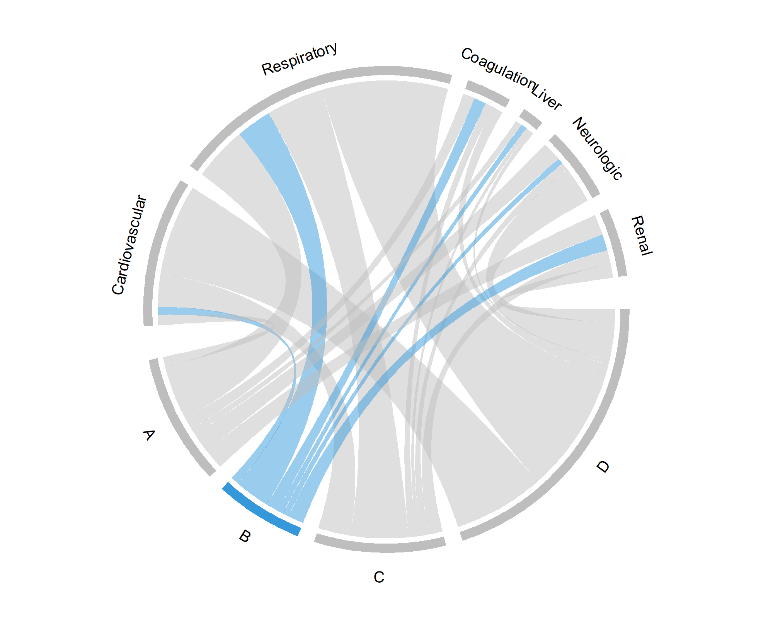


(iii) Phenotype B


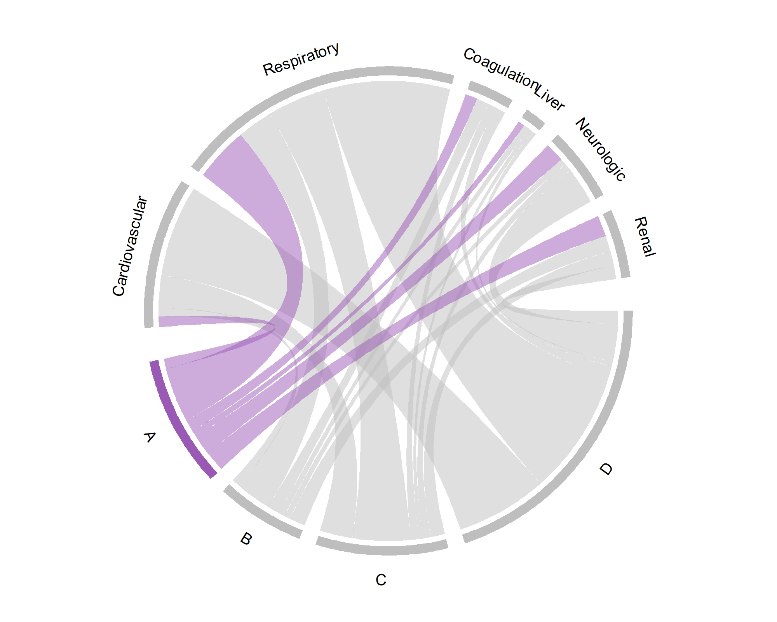


(ii) Phenotype A


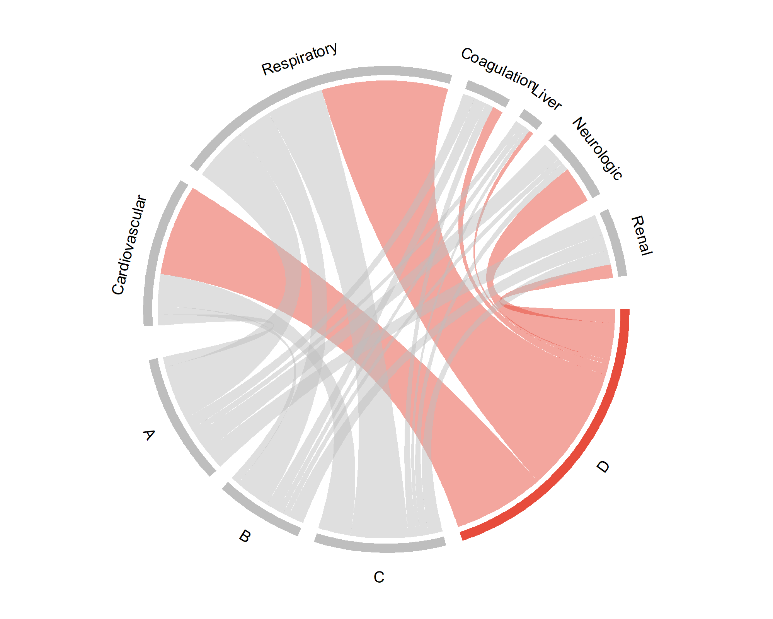


(v) Phenotype D


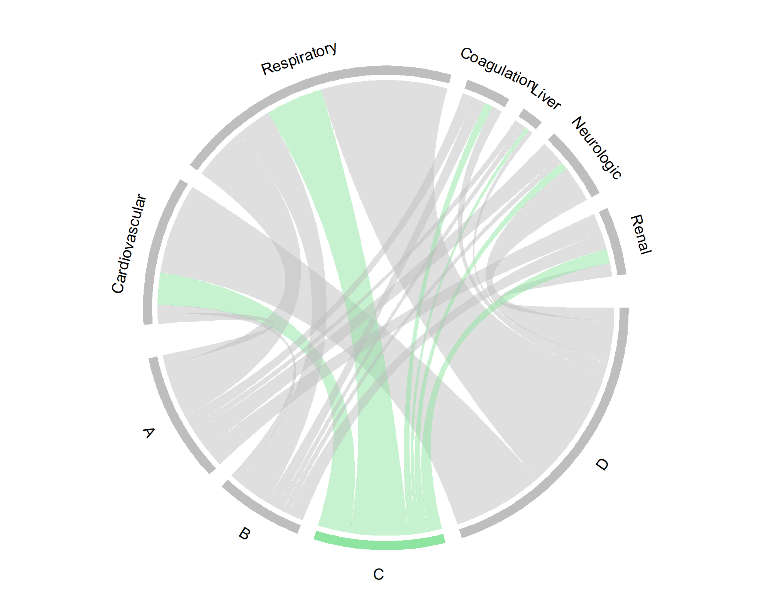


(iv) Phenotype C

For each phenotype, the larger percentage of patients with higher score of that organ system, the border the ribbon.

# eFigure 16. Survival curves and Cox proportional hazards modeling by phenotypes in the testing cohort


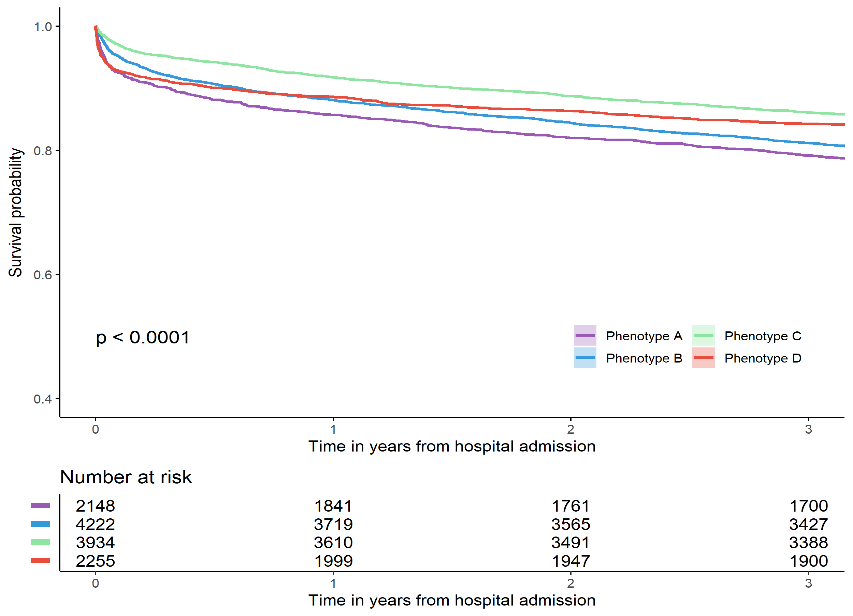


(A)


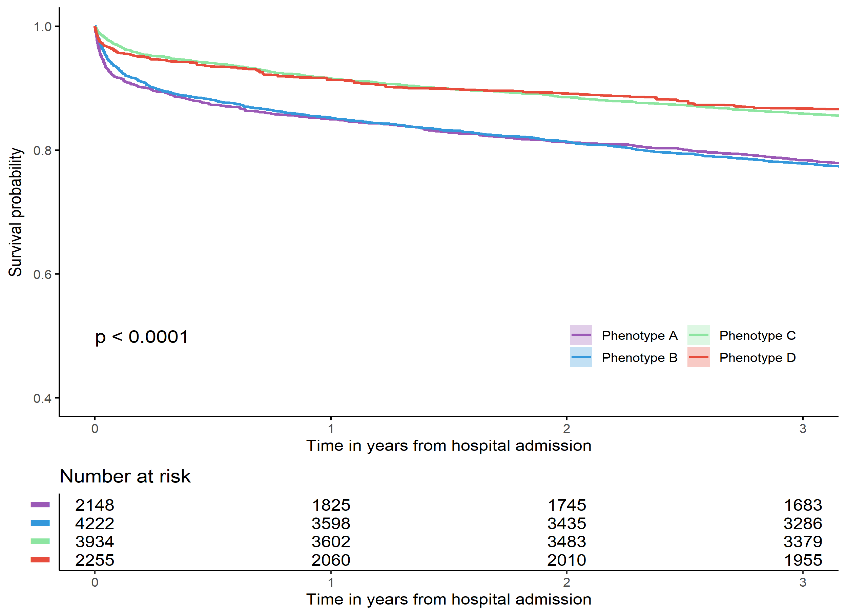


(C)


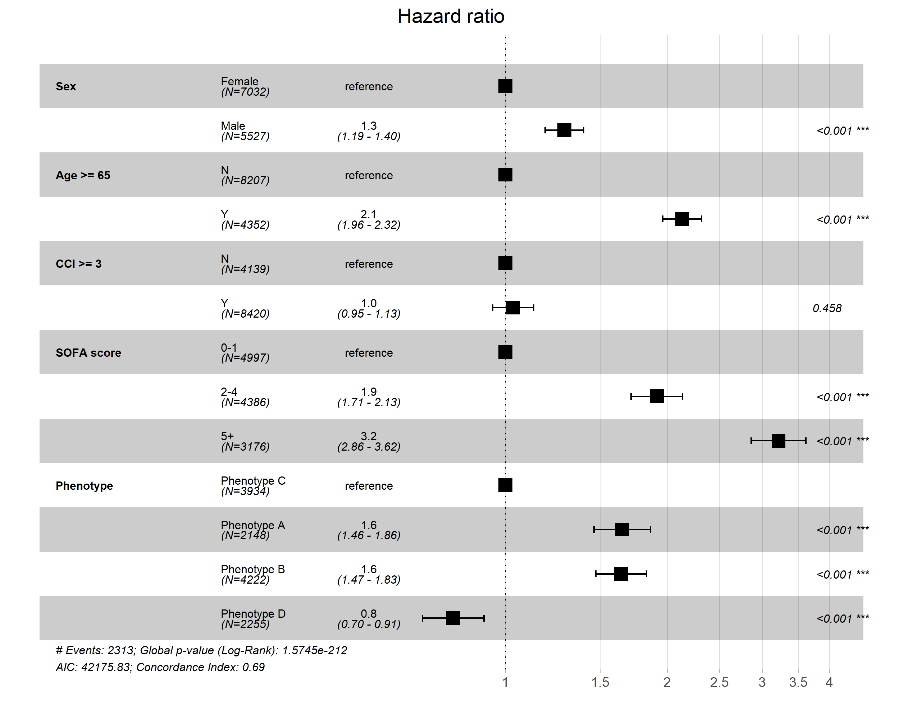


(D)


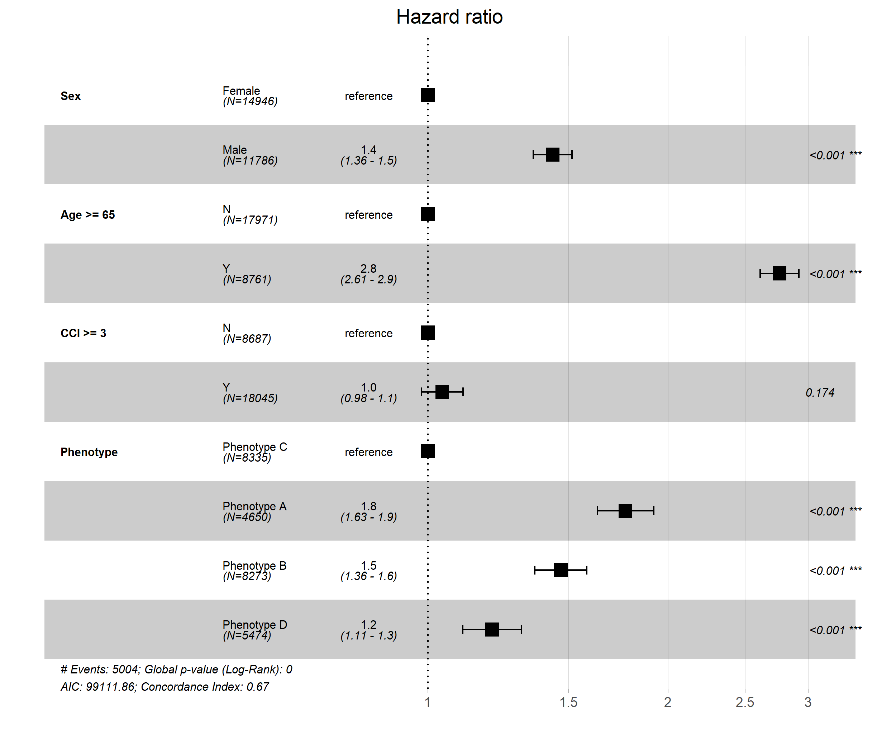


(B)

(A) Phenotype survival curves adjusted using demographic information and comorbidities. (B) Adjusted Cox proportional hazards models using demographic information and comorbidities. (C) Phenotype survival curves adjusted using demographic information, comorbidities, and SOFA scores. (D) Adjusted Cox proportional hazards model using demographic information, comorbidities, and SOFA scores. Abbreviation: CCI: Charlson comorbidity index; SOFA: sequential organ failure assessment.

# eFigure 17. Chord diagrams showing the distribution of nine most common admission diagnosis groups by phenotype in the testing cohort

(i) All phenotypes


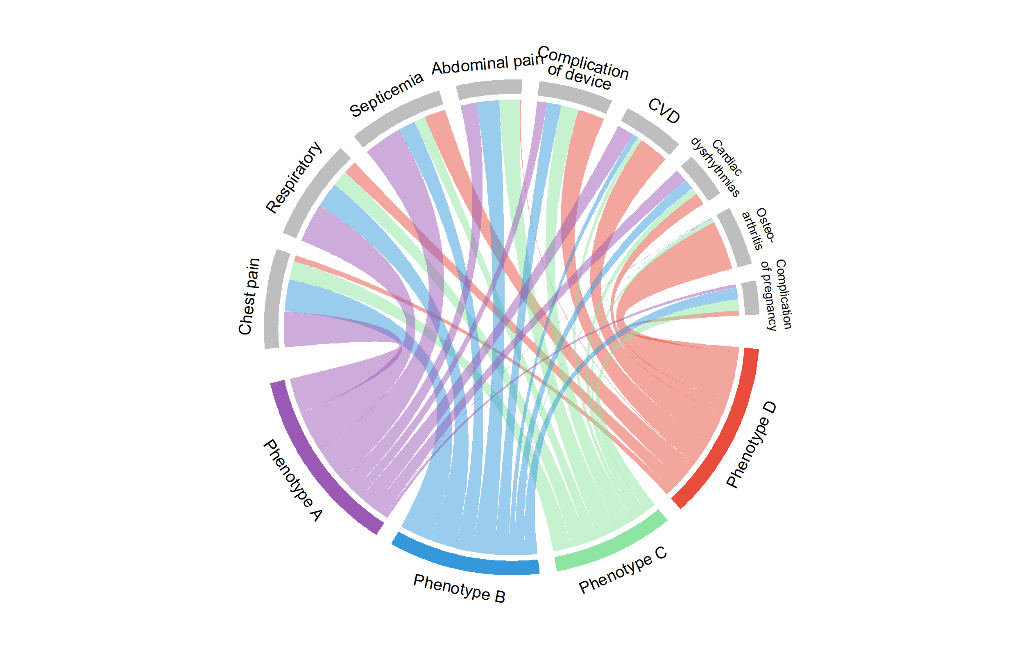


(ii) Phenotype A


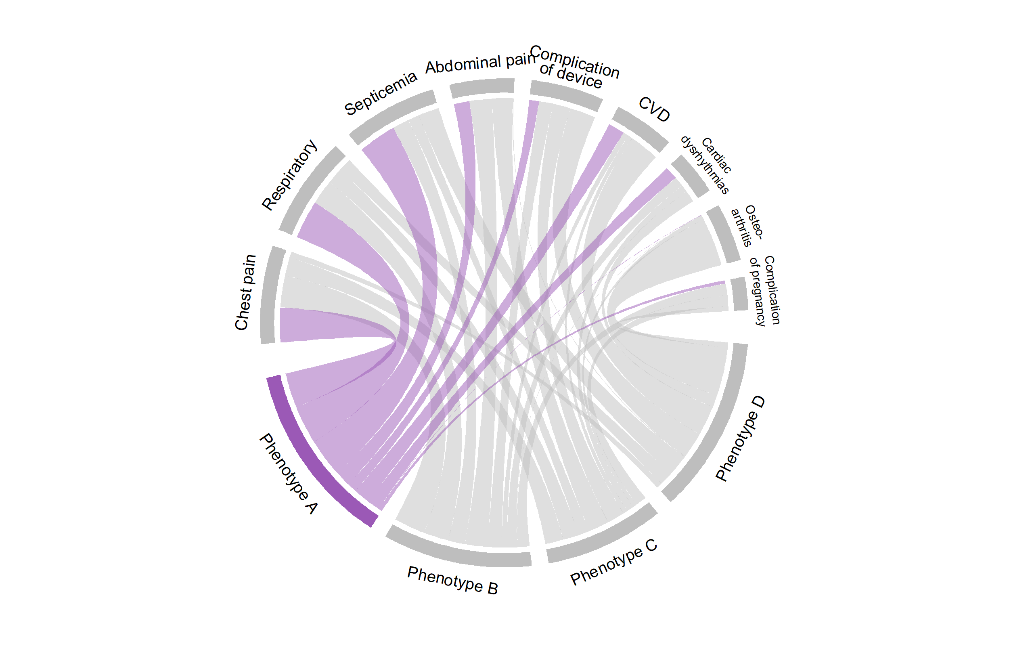


(iii) Phenotype B


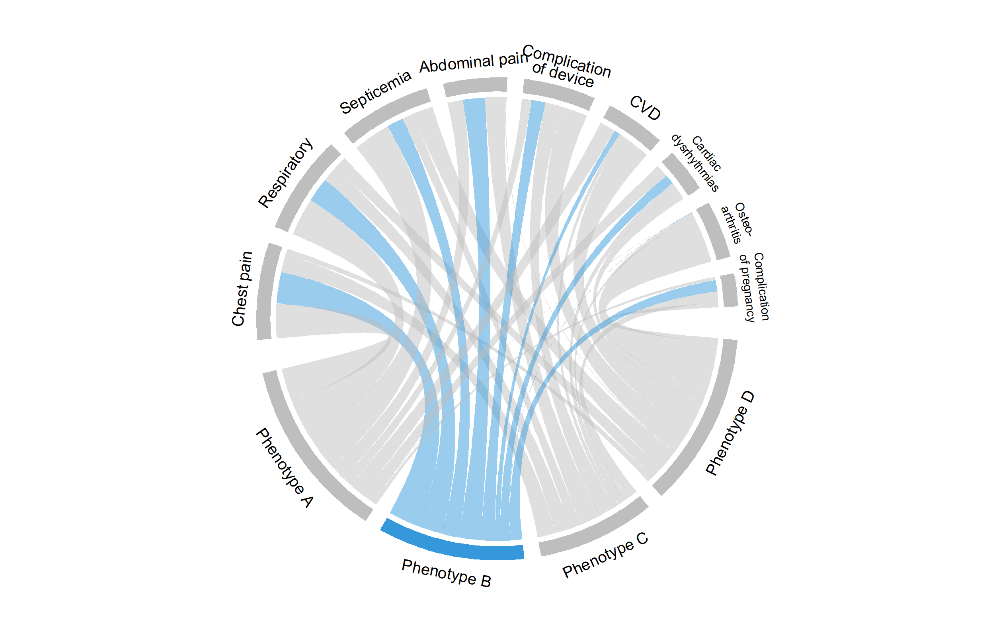

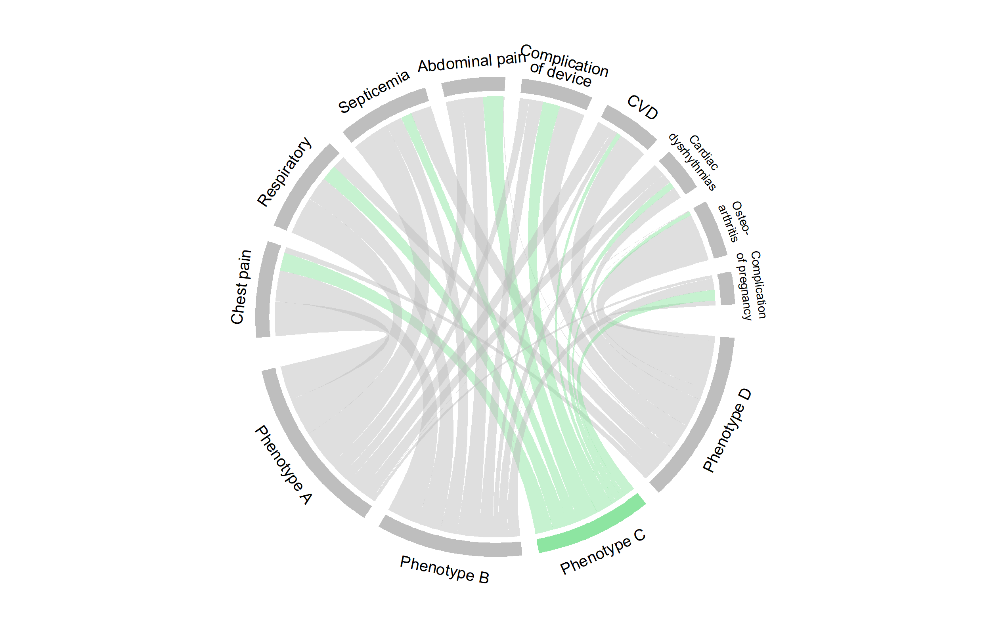

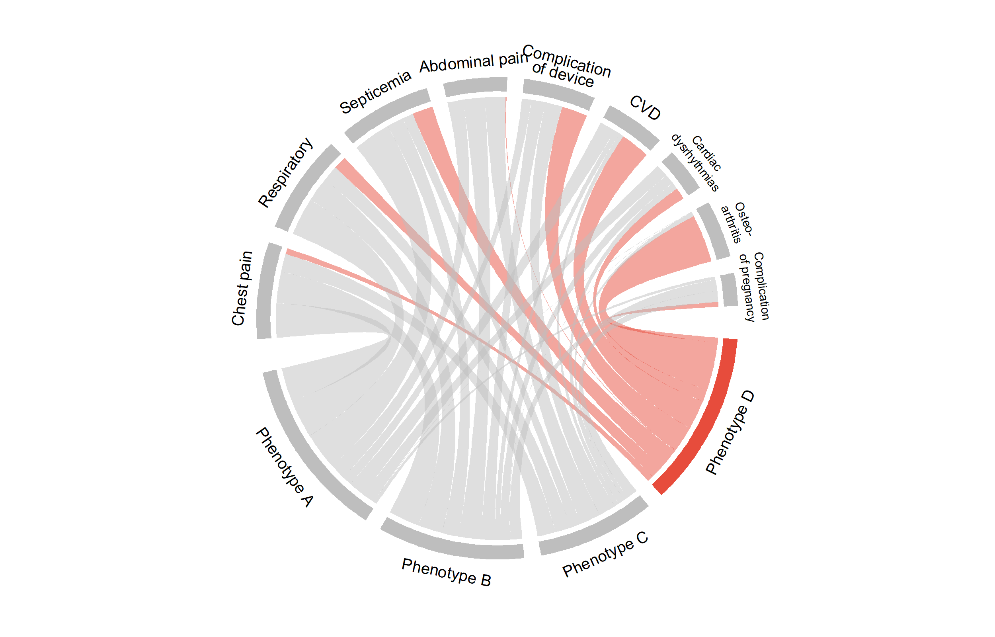


(v) Phenotype D

(iv) Phenotype C

Diagnosis groups are shown in order of frequencies of all patients. For each phenotype, the larger percentage of patients with that diagnosis, the border the ribbon. Detailed diagnosis groups from left to right are: Nonspecific chest pain, Other and unspecific lower respiratory disease, Septicemia (except in labor), Abdominal pain, Complication of device; implant or graft, Acute cerebrovascular disease, Cardiac dysrhythmias, Osteoarthritis, and Other complications of pregnancy.

# eFigure 18. Reconstruction error of physiologic signatures measured within six hours of hospital admission in training cohort using deep temporal interpolation and clustering network


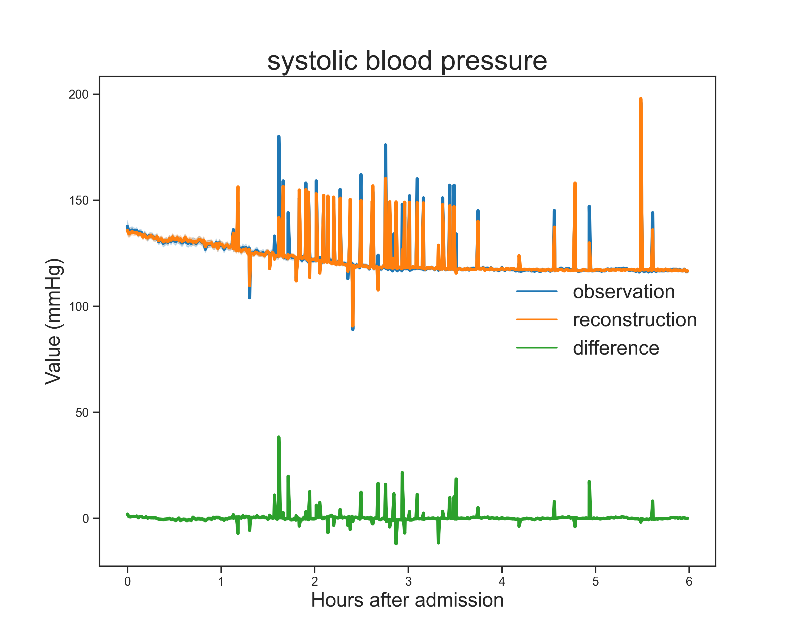

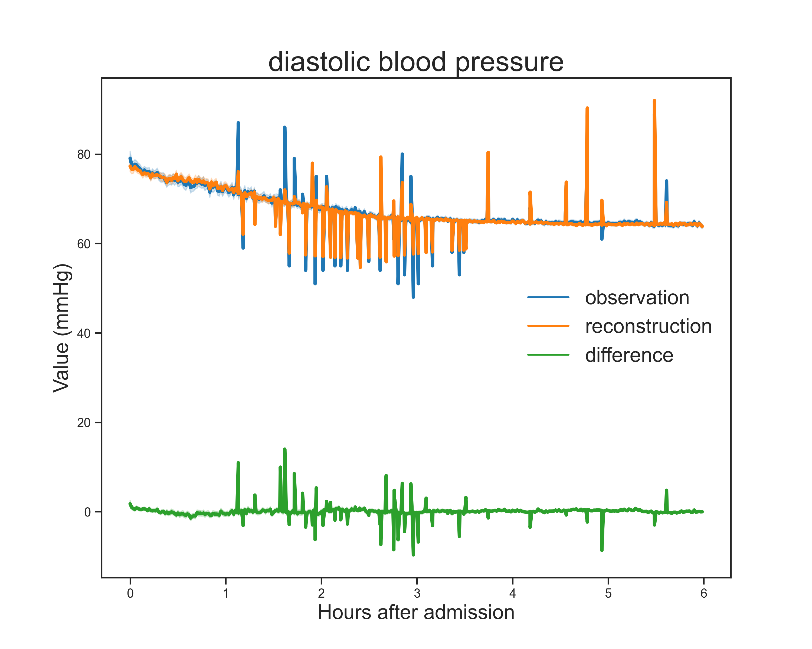

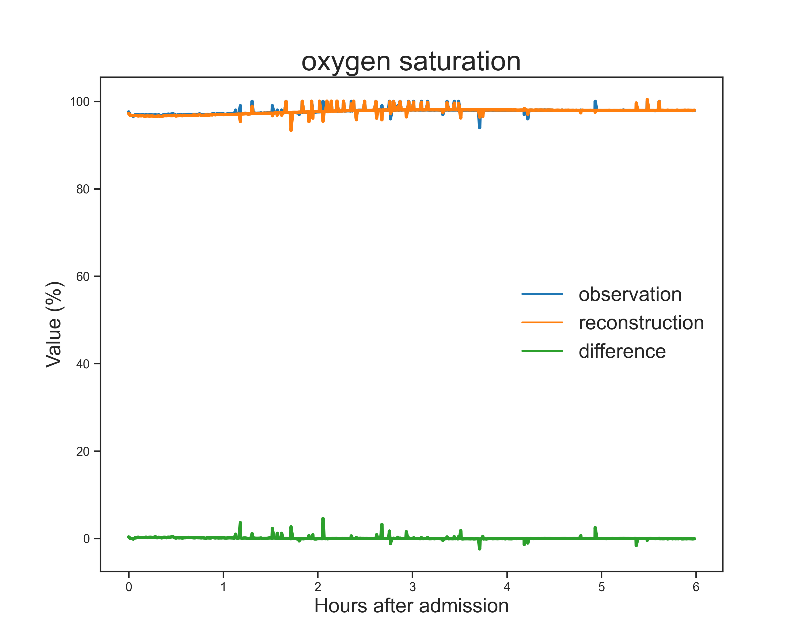

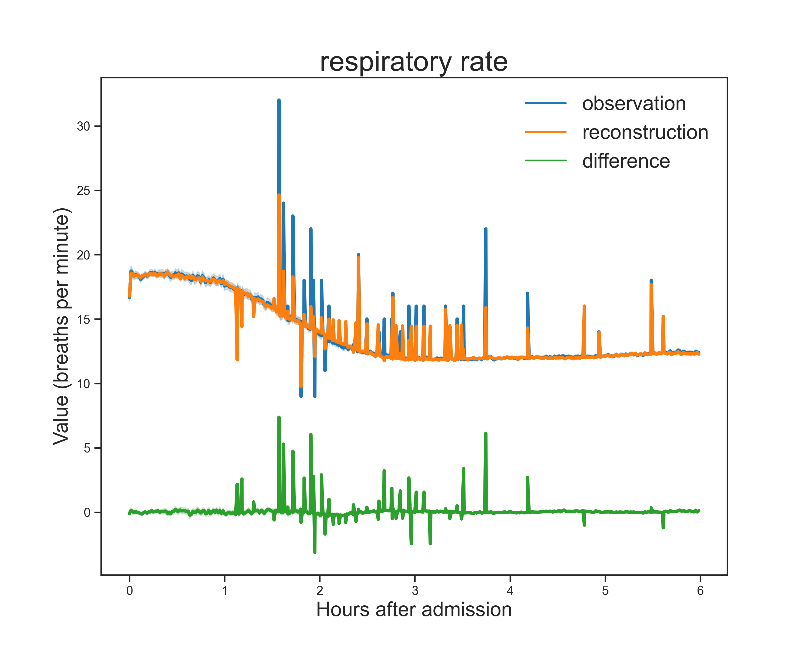

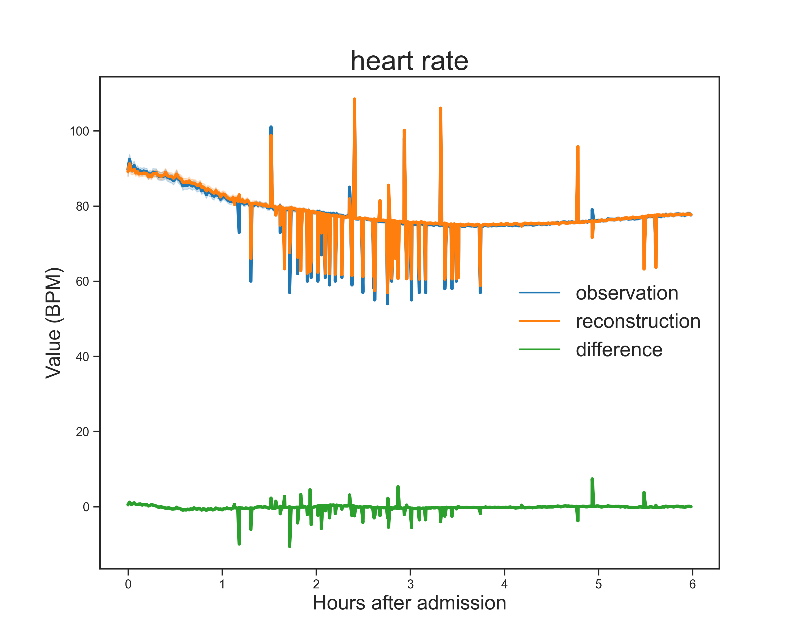

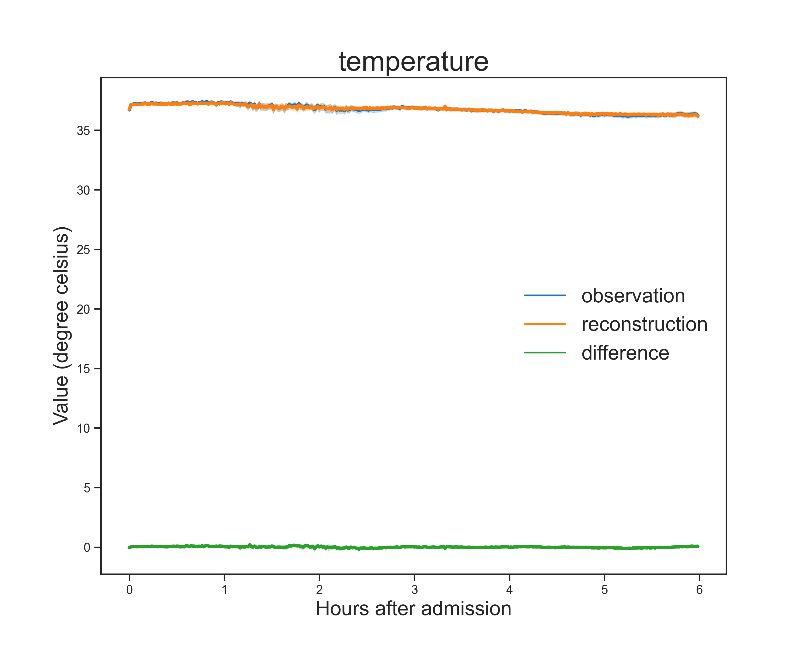


# eFigure 19. Reconstruction error of physiologic signatures measured within six hours of hospital admission in testing cohort using deep temporal interpolation and clustering network


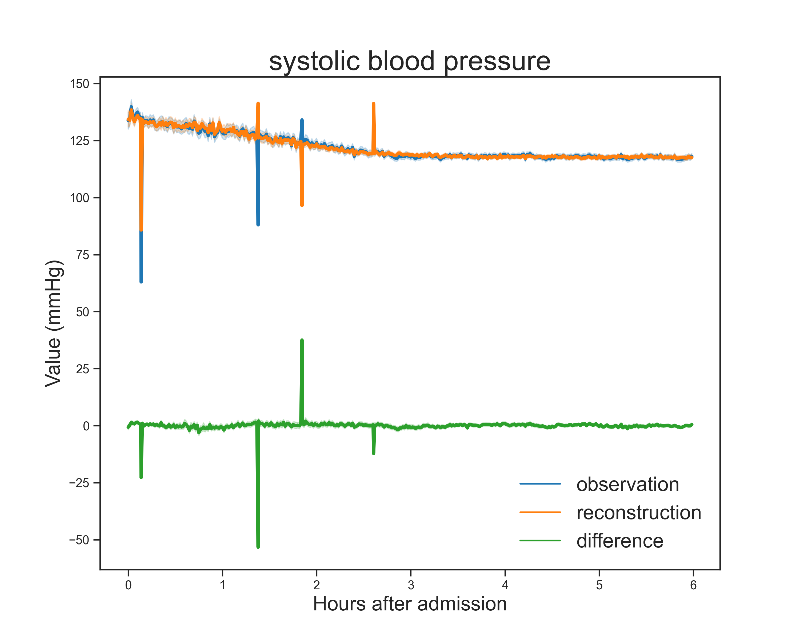

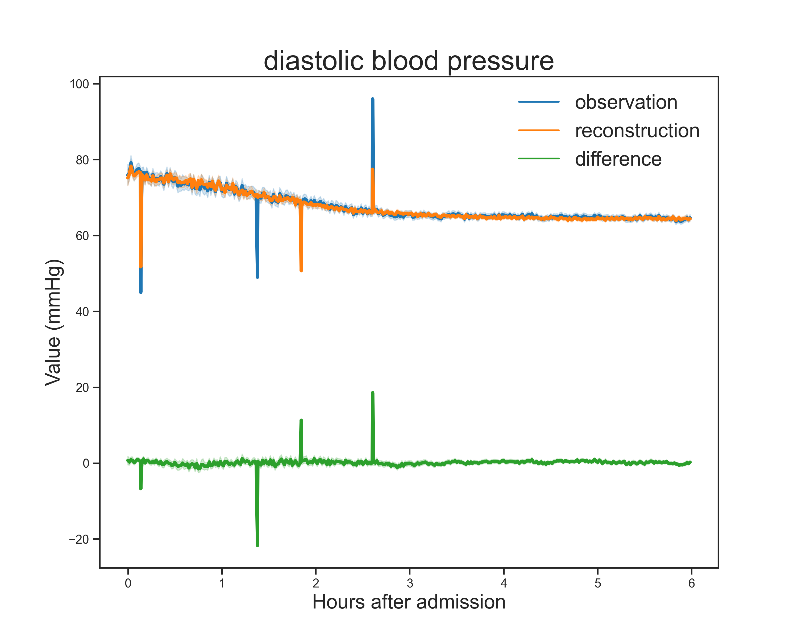

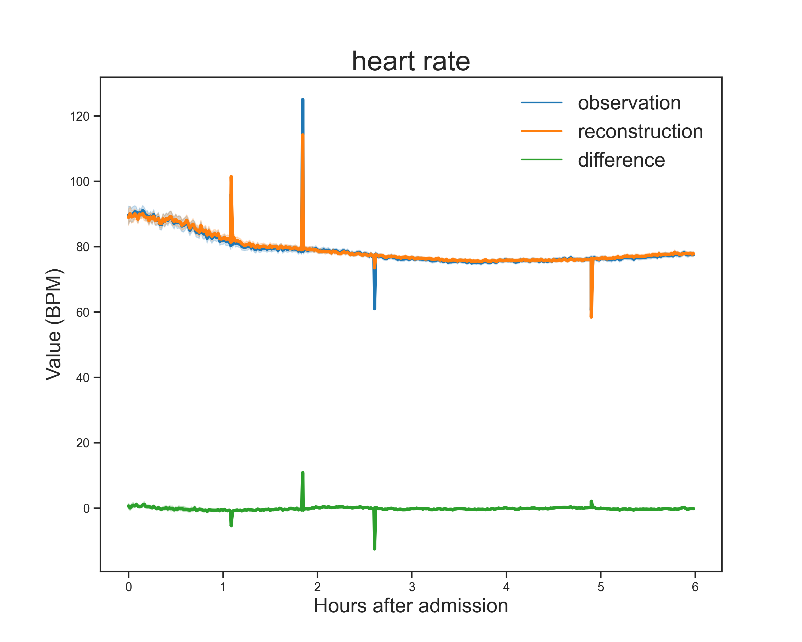

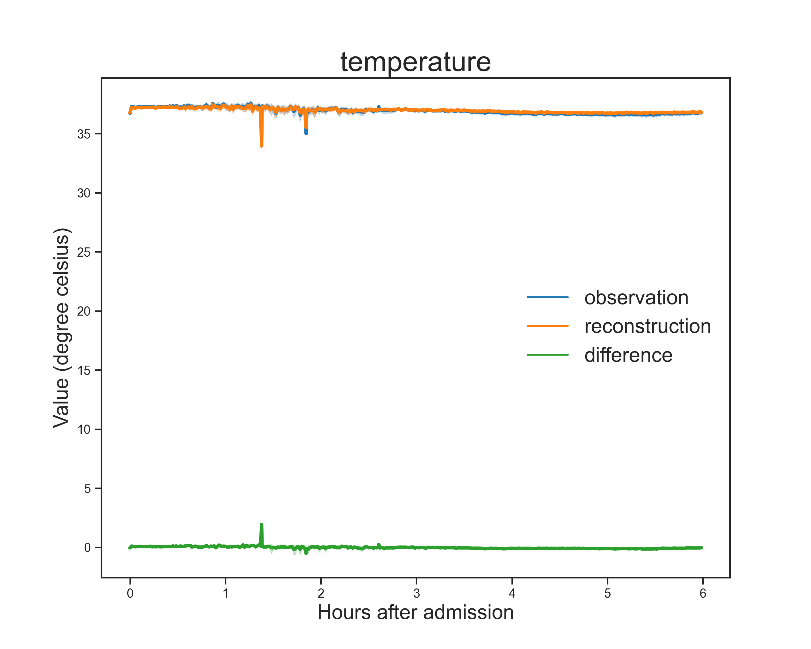

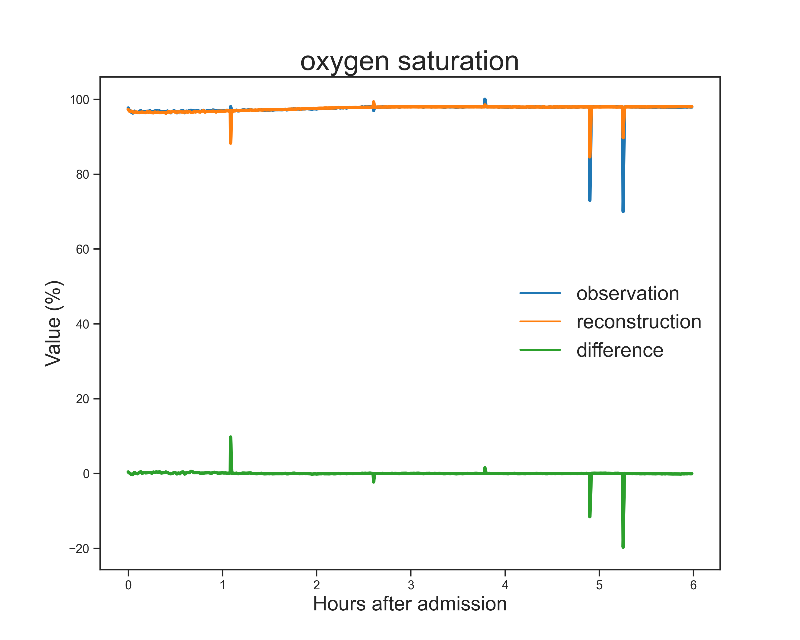

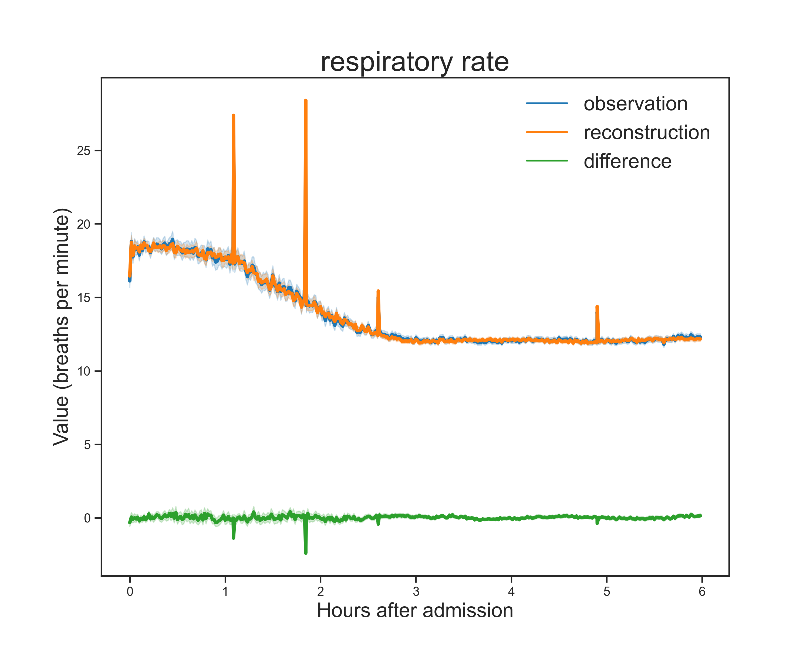


#

# eTable 1. Processing of vital sign time series

| Variables | Unit | Non-outlier range [min, max]^a^ | Frequency (per hour) | Missing any measurement (N = 75,762), n (%) | Values used to impute variable completely missing^b^ | Normal distribution |
| --- | --- | --- | --- | --- | --- | --- |
| Systolic blood pressure | mmHg | (20, 300) | 2 | 0 (0) | 119.1 | Yes |
| Diastolic blood pressure | mmHg | (5, 225) | 2 | 0 (0) | 66.1 | Yes |
| Heart rate | beats per minute | (0, 300] | 2 | 9 (0) | 76.8 | Yes |
| Temperature | degree Celsius | (24, 45) | 1 | 10,713 (14) | 36.7 | No |
| Peripheral capillary oxygen saturation | % | (1, 100] | 2 | 3,744 (5) | 97.8 | No |
| Respiratory rate | breaths per minute | (0, 60] | 2 | 470 (1) | 12.7 | Yes |

**^a^** Derived from expert-defined ranges. Open brackets “)” indicate value is not included and closed brackets “]” indicate value is included in the interval.

**^b^** For time series data missing entirely, including instances in which a variable was missing entirely from an admission, mean values of corresponding variables measured values in the training cohort were imputed.

#

# eTable 2. Used LOINCS, range of values, direction of abnormal values for lab variables

| Lab variables | LOINCS | LOINC Description | Plausible Range^a^ | Direction of abnormal value | Missingness in all cohort (N = 75,762), n (%) | Missingness in training cohort (N = 41,502), n (%) | Missingness in validation cohort (N = 17,415), n (%) | Missingness in testing cohort (N = 16,845), n (%) | Normal distribution |
| --- | --- | --- | --- | --- | --- | --- | --- | --- | --- |
| Basic metabolic Panel (BMP) | **89044-2/24321-2** | Basic metabolic and albumin panel - Serum or Plasma/Basic metabolic 2000 panel - Serum or Plasma |  |  |  |  |  |  |  |
| Glucose | 2339-0, 2340-8,  2345-7, 41651-1*,  41652-9*, 41653-7*,  74774-1*, | Glucose in serum or plasma/blood | 25 - 1400 | Maximum, Minimum | 9,090 (12) | 4,783 (12) | 2,129 (12) | 2,178 (13) | No |
| Creatinine | 2160-0, 38483-4 | Creatinine in blood | 0 - 30 | Maximum | 10,045 (13) | 5,276 (13) | 2,353 (14) | 2,416 (14) | No |
| Bilirubin | 1975-2 | Bilirubin total in serum or plasma | 0 - 50 | Maximum | 38,086 (50) | 20,319 (49) | 8,853 (51) | 8,914 (53) | No |
| Albumin | 1751-7, 2862-1,  61151-7 | Albumin in serum or plasma | 0.6 - 6.0 | Minimum | 37,735 (50) | 20,134 (49) | 8,779 (50) | 8,822 (52) | Yes |
| Anion Gap | 33037-3, 10366-1 | Anion gap in Serum or Plasma | 1 - 40 | Maximum | 14,876 (20) | 9,531 (23) | 2,645 (15) | 2,700 (16) | Yes |
| CBC Panel | 57021-8 | CBC W Auto Differential panel - Blood |  |  |  |  |  |  |  |
| White Blood Cell Count | 26464-8, 6690-2 | Leukocytes [#/volume] in Blood | 0.1 - 240 | Maximum,  Minimum | 7,118 (9) | 3,676 (9) | 1,665 (10) | 1,777 (11) | No |
| Hemoglobin | 718-7,  14775-1*, 30313-1*,  30352-9*, | Hemoglobin [Mass/volume] in Blood | 3 - 23 | Minimum | 6,108 (8) | 3,112 (7) | 1,443 (8) | 1,553 (9) | Yes |
| Platelets | 26515-7,  777-3, 49497-1* | Platelets [#/volume] in Blood | 2 - 1900 | Minimum | 7,143 (9) | 3,684 (9) | 1,671 (10) | 1,788 (11) | Yes |
| Bands % | 26508-2,  ,35332-6,  764-1* | Band form neutrophils/100 leukocytes in blood | 0.9 - 90 | Maximum | 72,187 (95) | 39,455 (95) | 16,625 (95) | 16,107 (96) | Yes |
| Lymphocytes % | 736-9, 737-7 | Lymphocytes/100 leukocytes in blood | 0 - 100 | Maximum,  Minimum | 22,653 (30) | 12,079 (29) | 5,275 (30) | 5,299 (31) | Yes |
| Gas Panel |  |  |  |  |  |  |  |  |  |
| Gas Panel-Arterial blood | 24336-0 |  |  |  |  |  |  |  |  |
| PH | 2744-1 | pH of arterial blood | 5 - 8 | Maximum,  Minimum | 64,519 (85) | 35,387 (85) | 14,894 (86) | 14,238 (85) | No |
| PO2 | 2703-7 | Oxygen [Partial pressure] in arterial blood | 0 - 800 | Minimum | 64,519 (85) | 35,386 (85) | 14,894 (86) | 14,239 (85) | No |
| Base deficit | 1922-4 | Base deficit in Arterial blood | 0 - 30 | Maximum | 69,565 (92) | 38,135 (92) | 15,986 (92) | 15,444 (92) | No |
| RDW | 788-0, 21000-5 | Erythrocyte distribution width [Ratio] | 2 - 40 | Maximum,  Minimum | 7,113 (9) | 3,672 (9) | 1,664 (10) | 1,777 (11) | Yes |
| Others |  |  |  |  |  |  |  |  |  |
| C-Reactive Protein (all sensitivity levels) | 30522-7, 1988-5 | C reactive protein in serum or plasma | 0 - 280 | Maximum | 65,389 (86) | 35,640 (86) | 15,159 (87) | 14,590 (87) | Yes |
| Lactate | 2518-9, 2524-7,  32693-4, 14118-4,  30242-2 | Lactate in blood | 0.3 - 28 | Maximum | 47,936 (63) | 26,055 (63) | 11,178 (64) | 10,703 (64) | No |
| ESR | 4537-7, 30341-2,  18184-2, 43402-7,  4538-5, 4539-3,  82477-1 | Erythrocyte sedimentation rate | 1 - 140 | Maximum | 69,214 (91) | 37,599 (91) | 16,033 (92) | 15,582 (93) | Yes |
| INR | 34714-6, 6301-6 | International normalized ratio | 0.8 - 18 | Maximum | 40,755 (54) | 21,145 (51) | 9,835 (56) | 9,775 (58) | No |
| Troponin (TnT, TnI) | 6598-7, 48425-3, 6597-9, 67151-1,10839-9, 42757-5,  49563-0 | Troponin T.cardiac in blood and Troponin I.cardiac in blood | 0 - 49 | Maximum | 49,379 (65) | 26,886 (65) | 11,553 (66) | 10,940 (65) | No |

^a^ Values out of the range values were removed.

Abbreviations: BUN: blood urea nitrogen; CO2: carbon dioxide; AST: aspartate transaminase; ALT: alanine transaminase; PO2: partial pressure of oxygen; RDW: red cell distribution width; ESR: erythrocyte sedimentation rate; INR: international normalized ratio.

*Included LOINCs that have same description from other panels.

# eTable 3. Clinic characteristics and biomarkers of the cohorts

| **Variables** | **Overall cohort** | **Training cohort** | **Validation Cohort** | **Testing Cohort** |
| --- | --- | --- | --- | --- |
| Number of encounters (%) | 75,762 | 41,502 (55) | 17,415 (23) | 16,845 (22) |
| **Preadmission clinical characteristics** |  |  |  |  |
| Age, mean (SD) | 54 (19) | 54 (19) | 54 (19)^a^ | 55 (19)^a,b^ |
| Female sex, n (%) | 41,449 (55) | 22,745 (55) | 9,499 (55) | 9,205 (55) |
| Race, n (%) |  |  |  |  |
| White | 53,101 (70) | 29,076 (70) | 12,171 (70) | 11,854 (70) |
| African American | 17,432 (23) | 9,634 (23) | 3,953 (23) | 3,845 (23) |
| Primary insurance, n (%) |  |  |  |  |
| Private | 17,641 (23) | 9,591 (23) | 4,115 (24) | 3,935 (23) |
| Medicare | 33,969 (45) | 18,499 (45) | 7,625 (44) | 7,845 (47)^a,b^ |
| Medicaid | 16,742 (22) | 9,231 (22) | 3,919 (23) | 3,592 (21)^a,b^ |
| Uninsured | 7,410 (10) | 4,181 (10) | 1,756 (10) | 1,473 (9)^a,b^ |
| Residency area characteristics |  |  |  |  |
| Total proportion of African American (%), mean (SD) | 18.8 (17.5) | 18.7 (17.5) | 18.9 (17.5) | 18.7 (17.4) |
| Proportion below poverty (%), mean (SD) | 22.6 (10.1) | 22.7 (10.1) | 22.7 (10.3) | 22.5 (10.2)^a^ |
| Distance from hospital (mile), median (IQR) | 18 (3, 34) | 18 (3, 34) | 18 (3, 34) | 18 (3, 34) |
| **Comorbidities** |  |  |  |  |
| Hypertension, n (%) | 38,985 (51) | 21,639 (52) | 8,878 (51)^a^ | 8,468 (50)^a^ |
| Cardiovascular disease, n (%)^c^ | 21,743 (29) | 12,058 (29) | 4,983 (29) | 4,702 (28)^a^ |
| Diabetes mellitus, n (%) | 18,127 (24) | 10,111 (24) | 4,071 (23)^a^ | 3,945 (23)^a^ |
| Chronic kidney disease, n (%) | 12,357 (16) | 6,518 (16) | 2,947 (17)^a^ | 2,892 (17)^a^ |
| **Admission characteristics of patients** |  |  |  |  |
| Emergent admission, n (%) | 55,008 (73) | 30,177 (73) | 12,542 (72) | 12,289 (73) |
| Transfer from another hospital, n (%) | 13,569 (18) | 7,115 (17) | 3,595 (21)^a^ | 2,859 (17)^b^ |
| **Primary admission diagnostic groups** |  |  |  |  |
| Diseases of the circulatory system, n (%) | 13,670 (18) | 7,719 (19) | 2,968 (17)^a^ | 2,983 (18)^a^ |
| Respiratory and infectious diseases, n (%) | 6,016 (8) | 3,306 (8) | 1,185 (7)^a^ | 1,525 (9)^a,b^ |
| Complications of pregnancy and childbirth, n (%) | 5,760 (8) | 3,148 (8) | 1,366 (8) | 1,246 (7) |
| Diseases of the digestive/genitourinary systems, n (%) | 9,532 (13) | 5,184 (12) | 2,201 (13) | 2,147 (13) |
| Diseases of the musculoskeletal/connective tissue and skin, n (%) | 6,591 (9) | 3,651 (9) | 1,522 (9) | 1,418 (8) |
| Neoplasms, n (%) | 4,953 (7) | 2,743 (7) | 1,136 (7) | 1,074 (6) |
| **Clinical biomarkers and interventions within 24 hours of admission** |  |  |  |  |
| Surgery on admission day, n (%) | 15,996 (21) | 8,644 (21) | 3,801 (22)^a^ | 3,551 (21) |
| ICU/IMC admission within first 24 hours, n (%) | 17,163 (23) | 9,426 (23) | 3,899 (22) | 3,838 (23) |
| **Cardiovascular system** |  |  |  |  |
| Hypotension (MAP < 60 mmHg) at any time, n (%) | 26,400 (35) | 14,470 (35) | 6,014 (35) | 5,916 (35) |
| Duration, median (IQR), minutes | 57 (15, 165) | 57 (15, 168) | 53 (14, 157) | 60 (15, 167)^b^ |
| Vasopressors used, n (%) | 13,991 (18) | 7,531 (18) | 3,294 (19) | 3,166 (19) |
| Out of operating room | 2,596 (3) | 1,403 (3) | 625 (4) | 568 (3) |
| Hypertension (SBP > 160 mmHg) at any time, n (%) | 2,7267 (36) | 14,838 (36) | 6,222 (36) | 6,207 (37)^a^ |
| Duration, median (IQR), minutes | 120 (26, 358) | 120 (27, 356) | 120 (25, 373) | 120 (26, 352) |
| Troponin, tested, n (%) | 26,383 (35) | 14,616 (35) | 5,862 (34)^a^ | 5,905 (35)^b^ |
| Abnormal result among tested, n (%) | 5,842 (22) | 3,398 (23) | 1,239 (21)^a^ | 1,205 (20)^a^ |
| **Respiratory system** |  |  |  |  |
| Highest administered FiO2, median (IQR) | 0.21 (0.21, 0.40) | 0.21 (0.21, 0.40) | 0.21 (0.21, 0.40) | 0.21 (0.21, 0.40) |
| Room air only, n (%) | 43,887 (58) | 23,963 (58) | 10,242 (59)^a^ | 9,682 (57)^b^ |
| 0.22 - 0.40, n (%) | 26,997 (36) | 14,790 (36) | 6,125 (35) | 6,082 (36) |
| > 0.4, n (%) | 4,878 (6) | 2,749 (7) | 1,048 (6)^a^ | 1,081 (6) |
| PaO2/FiO2, tested with arterial blood gas, n (%) | 11,235 (15) | 6,113 (15) | 2,519 (14) | 2,603 (15)^b^ |
| <200 among tested, n (%) | 4,137 (37) | 2,265 (37) | 908 (36) | 964 (37) |
| Mechanical ventilation, n (%) | 3,924 (5) | 2,123 (5) | 883 (5) | 918 (5) |
| **Kidney and acid-base status** |  |  |  |  |
| Preadmission estimated glomerular filtration rate^d^ (mL/min per 1.73 m2), median (IQR) | 95 (77, 111) | 95 (78, 111) | 95 (76, 111) | 94 (76, 110)^a^ |
| Highest / reference creatinine^d^, mean (SD) | 1.24 (0.75) | 1.24 (0.66) | 1.24 (0.74)^a^ | 1.23 (0.95)^a^ |
| Renal replacement therapy, n (%) | 1,166 (1.5) | 641 (1.5) | 257 (1.5) | 268 (1.6) |
| Highest anion gap, median (IQR), mmol/L | 14 (12, 17) | 14 (12, 17) | 14 (13, 17)^a^ | 14 (12, 17)^a,b^ |
| Arterial blood gas tested, n (%) | 11,242 (15) | 6,115 (15) | 2,521 (14) | 2,606 (15)^b^ |
| pH < 7.3 among tested, n (%) | 2,580 (23) | 1,437 (23) | 557 (22) | 586 (22) |
| Highest base deficit, mean (SD), mmol/L | 4.8 (4.7) | 4.8 (4.7) | 4.9 (4.6) | 4.6 (4.8)^a,b^ |
| Lactate, tested, n (%) | 27,826 (37) | 15,447 (37) | 6,237 (36)^a^ | 6,142 (36) |
| 2 - 4 mmol/L among tested, n (%) | 6,717 (24) | 3,739 (24) | 1,498 (24) | 1,480 (24) |
| > 4 mmol/L among tested, n (%) | 2,532 (9) | 1,374 (9) | 578 (9) | 580 (9) |
| **Inflammation** |  |  |  |  |
| Highest white blood cell count, median (IQR), x10^9/L | 9 (7, 13) | 9 (7, 13) | 9 (7, 13)^a^ | 9 (7, 12)^a^ |
| Highest premature neutrophils (bands)), median (IQR), % | 10 (4, 20) | 10 (4, 20) | 9 (3, 19) | 9 (4, 18) |
| Lowest lymphocytes, median (IQR), % | 16 (9, 24) | 16 (9, 24) | 16 (9, 24) | 16 (9, 24) |
| C-reactive protein, tested, n (%) | 10373 (14) | 5862 (14) | 2256 (13)^a^ | 2255 (13) |
| Highest C-reactive protein, median (IQR), mg/L | 18 (5, 81) | 18 (5, 77) | 17 (4, 80) | 28 (5, 93)^a,b^ |
| Erythrocyte sedimentation rate, tested, n (%) | 6548 (9) | 3903 (9) | 1382 (8)^a^ | 1263 (7)^a^ |
| Highest erythrocyte sedimentation rate, median (IQR), mm/h | 40 (19, 74) | 40 (19, 73) | 42 (19, 77) | 41 (20, 73) |
| Highest temperature, mean (SD), Celsius | 37.7 (0.6) | 37.7 (0.6) | 37.7 (0.6) | 37.7 (0.6)^a,b^ |
| 38 - 39, n (%) | 15,774 (21) | 8,633 (21) | 3,563 (20) | 3,578 (21) |
| > 39, n (%) | 2,779 (4) | 1,548 (4) | 604 (3) | 627 (4) |
| Lowest temperature, mean (SD), Celsius | 36.7 (0.9) | 36.7 (1.0) | 36.7 (0.9)^a^ | 36.8 (0.9)^a,b^ |
| **Hematologic** |  |  |  |  |
| Lowest hemoglobin, mean (SD), g/dL | 11.4 (2.3) | 11.5 (2.3) | 11.4 (2.3)^a^ | 11.2 (2.3)^a,b^ |
| Highest RDW, mean (SD), % | 15.4 (2.1) | 15.5 (2.1) | 15.2 (2.1)^a^ | 15.3 (2.1)^a,b^ |
| Lowest platelets, median (IQR), x10^9/L | 208 (160, 266) | 210 (161, 269) | 204 (157, 260)^a^ | 207 (160, 266)^a,b^ |
| Platelets < 200, n (%) | 31,078 (41) | 16,707 (40) | 7,489 (43)^a^ | 6,882 (41)^a^ |
| < 100 | 4,779 (15) | 2,643 (16) | 1,128 (15) | 1,008 (15) |
| 100 - 200 | 26,299 (85) | 14,064 (84) | 6,361 (85) | 5,874 (85) |
| International normalized ratio, tested, n (%) | 35,007 (46) | 20,357 (49) | 7,580 (44)^a^ | 7,070 (42)^a,b^ |
| >= 2 | 3,291 (9) | 1,836 (9) | 757 (10)^a^ | 698 (10) |
| **Neurologic** |  |  |  |  |
| Glasgow Coma Scale score, n (%) |  |  |  |  |
| Moderate (9 - 12) | 3,125 (4) | 1,708 (4) | 687 (4) | 730 (4) |
| Severe (<= 8) | 2,662 (4) | 1,482 (4) | 587 (3) | 593 (4) |
| **Liver and metabolic** |  |  |  |  |
| Bilirubin, tested, n (%) | 37,676 (50) | 21,183 (51) | 8,562 (49)^a^ | 7931 (47)^a,b^ |
| >= 2 mg/dL, n (%) | 2,530 (7) | 1,427 (7) | 607 (7) | 496 (6) |
| Highest glucose, median (IQR), mg/dL | 127 (104, 170) | 126 (104, 170) | 126 (104, 169) | 128 (104, 172)^a,b^ |
| Albumin, tested, n (%) | 38,027 (50) | 21,368 (51) | 8,636 (50)^a^ | 8,023 (48)^a,b^ |
| < 2.5 | 2,159 (6) | 1,243 (6) | 471 (5) | 445 (6) |
| 2.5 - 3.5 | 12,118 (32) | 6,904 (32) | 2,665 (31)^a^ | 2,549 (32) |

Abbreviation: ICU: intensive care unit; IMC: intermediate care unit; MAP: mean arterial pressure; SBP: systolic blood pressure; RDW: red cell distribution width; SD: standard deviation; IQR: interquartile range.

All p values were adjusted for multiple comparisons using the Bonferroni method.

^a^ p < 0.05 compared to training cohort.

^b^ p < 0.05 compared to validation cohort.

^c^ Cardiovascular disease was considered if there was a history of congestive heart failure, coronary artery disease, or peripheral vascular disease.

^d^ Reference glomerular filtration rate and reference creatinine were derived without use of race correction (see eMethods for details).

# eTable 4. Illness severity, clinical outcomes, and resource use of the cohorts

| **Variables** | **Overall cohort** | **Training cohort** | **Validation Cohort** | **Testing Cohort** |
| --- | --- | --- | --- | --- |
| Number of encounters (%) | 75,762 | 41,502 (55) | 17,415 (23) | 16,845 (22) |
| **Acuity scores within 24h of admission** |  |  |  |  |
| SOFA score > 6, n (%) | 6,463 (9) | 3,506 (8) | 1,503 (9) | 1,454 (9) |
| Patients in ICU/IMC, SOFA score <= 6, n (%) | 12,477 (16) | 6,882 (17) | 2,795 (16) | 2,800 (17) |
| Patients in ICU/IMC, SOFA score > 6, n (%) | 4,686 (6) | 2,544 (6) | 1,104 (6) | 1,038 (6) |
| Patients in ward, SOFA score <= 6, n (%) | 56,822 (75) | 31,114 (75) | 13,117 (75) | 12,591 (75) |
| Patients in ward, SOFA score > 6, n (%) | 1,777 (2) | 962 (2) | 399 (2) | 416 (2) |
| MEWS score > 4, n (%) | 5,033 (7) | 2,828 (7) | 1,115 (6) | 1,090 (6) |
| Patients in ICU/IMC, MEWS score <= 4, n (%) | 13,316 (18) | 7,235 (17) | 3,041 (17) | 3,040 (18) |
| Patients in ICU/IMC, MEWS score > 4, n (%) | 3,847 (5.1) | 2,191 (5.3) | 858 (4.9) | 798 (4.7)^a^ |
| Patients in ward, MEWS score <= 4, n (%) | 57,413 (76) | 31,439 (76) | 13,259 (76) | 12,715 (75) |
| Patients in ward, MEWS score > 4, n (%) | 1,186 (2) | 637 (2) | 257 (1) | 292 (2) |
| **Resource use during hospitalization** |  |  |  |  |
| Hospital days, median (IQR) | 4 (2, 7) | 4 (2, 7) | 4 (2, 7) | 4 (2, 7) |
| Surgery at any time, n (%) | 21,436 (28) | 11,634 (28) | 5,084 (29)^a^ | 4,718 (28)^b^ |
| Admitted to ICU/IMC^c^, n (%) | 20,380 (27) | 11,121 (27) | 4,643 (27) | 4,616 (27) |
| Days in ICU/IMC^d^, median (IQR) | 4 (2, 7) | 4 (2, 7) | 4 (2, 7) | 4 (2, 7)^a,b^ |
| More than 48 hrs in ICU/IMC^d^, n (%) | 15,201 (75) | 8,332 (75) | 3,468 (75) | 3,401 (74) |
| Mechanical ventilation, n (%) | 5,970 (8) | 3,218 (8) | 1,403 (8) | 1,349 (8) |
| Hours on mechanical ventilation^e^, median (IQR) | 32 (12, 108) | 35 (14, 113) | 31 (11, 105)^a^ | 28 (10, 92)^a^ |
| More than 48 hrs on mechanical ventilation^e^, n (%) | 2,993 (50) | 1,661 (52) | 699 (50) | 633 (47)^a^ |
| Renal replacement therapy, n (%) | 2,316 (3) | 1,262 (3) | 524 (3) | 530 (3) |
| **Complications** |  |  |  |  |
| Acute kidney injury overall, n (%) | 12,547 (17) | 6,905 (17) | 2,901 (17) | 2,741 (16) |
| Community-acquired AKI, n (%) | 7,007 (56) | 3,839 (56) | 1,603 (55) | 1,565 (57) |
| Hospital-acquired AKI, n (%) | 5,540 (44) | 3,066 (44) | 1,298 (45) | 1,176 (43) |
| Worst AKI staging, n (%) |  |  |  |  |
| Stage 1 | 8,036 (64) | 4,360 (63) | 1,878 (65) | 1,798 (66) |
| Stage 2 | 2,407 (19) | 1,362 (20) | 533 (18) | 512 (19) |
| Stage 3 | 1,496 (12) | 848 (12) | 348 (12) | 300 (11) |
| Stage 3 with RRT | 608 (5) | 335 (5) | 142 (5) | 131 (5) |
| Venous thromboembolism, n (%) | 2,902 (4) | 1,257 (3) | 708 (4)^a^ | 937 (6)^a,b^ |
| Sepsis, n (%) | 7,322 (10) | 3,750 (9) | 1,659 (10) | 1,913 (11)^a,b^ |
| Hospital disposition, n (%) |  |  |  |  |
| Hospital mortality | 2,134 (2.8) | 1,141 (2.7) | 480 (2.8) | 513 (3.0) |
| Another hospital, LTAC, SNF, Hospice | 8,423 (11.1) | 4,475 (10.8) | 2,002 (11.5)^a^ | 1,946 (11.6)^a^ |
| Home or short-term rehabilitation | 65,205 (86.1) | 35,886 (86.5) | 14,933 (85.7) | 14,386 (85.4)^a^ |
| 30-day mortality, n (%) | 2,984 (4) | 1,633 (4) | 646 (4) | 705 (4) |
| Three-year mortality, n (%) | 14,634 (19) | 8,013 (19) | 3,297 (19) | 3,324 (20) |

Abbreviation: SOFA: sequential organ failure assessment; MEWS: modified early warning score; ICU: intensive care unit; IMC: intermediate care unit; SD: standard deviation; IQR: interquartile range.

All p-values were adjusted for multiple comparisons using the Bonferroni method.

^a^ p < 0.05 compared to training cohort.

^b^ p < 0.05 compared to validation cohort.

^c^ ICU/IMC admission rate was derived from data collected at any point during the hospitalization period. When an encounter had multiple ICU admissions throughout the hospitalization, the admission rate will only be evaluated once.

^d^ The length of ICU stay and prolonged ICU stay (greater than 48 hours) were derived for only patients who were admitted to the ICU or IMC.

^e^ The duration of receiving mechanical ventilation and prolonged mechanical ventilation (greater than 48 hours) were derived for only patients who required mechanical ventilation.

# eTable 5. Statistic output from the deep interpolation network modeling in the training cohort.

|  | **Statics** | | **Class size (N = 41,502), n (%)** | | | | | | | | | |
| --- | --- | --- | --- | --- | --- | --- | --- | --- | --- | --- | --- | --- |
| **Class number** | **Sihouette^a^** | **Davies-Bouldin Index^b^** | **1** | **2** | **3** | **4** | **5** | **6** | **7** | **8** | **9** | **10** |
| 2 | 0.60 | 0.77 | 34,750 (84) | 6,752 (16) | . | . | . | . | . | . | . | . |
| 3 | 0.33 | 1.04 | 6,110 (15) | 17,450 (42) | 17,942 (43) | . | . | . | . | . | . | . |
| 4 | 0.30 | 1.27 | 14,356 (35) | 4,238 (10) | 5,078 (12) | 17,830 (43) | . | . | . | . | . | . |
| 5 | 0.30 | 1.24 | 5,016 (12) | 17,401 (42) | 4,844 (12) | 13,886 (33) | 355 (0.9) | . | . | . | . | . |
| 6 | 0.24 | 1.42 | 14,307 (34) | 3,985 (10) | 2,631 (6) | 8,261 (20) | 11,965 (29) | 353  (0.9) | . | . | . | . |
| 7 | 0.23 | 1.42 | 8,496 (20) | 3,030 (7) | 11,834 (29) | 1,956 (5) | 14,019 (34) | 354 (0.9) | 1,813 (4) | . | . | . |
| 8 | 0.24 | 1.453 | 11,824 (28) | 1,229 (3) | 13,990 (34) | 2,841 (7) | 8,477 (20) | 345 (0.8) | 1,068 (3) | 1,728 (4) | . | . |
| 9 | 0.19 | 1.60 | 10,106 (24) | 1,227 (3) | 10,218 (25) | 9,701 (23) | 344 (0.8) | 2,769 (7) | 1,038 (3) | 1,589 (4) | 4,510 (11) | . |
| 10 | 0.18 | 1.63 | 2,746 (7) | 6,096 (15) | 10,041 (24) | 1,582 (4) | 1,221 (3) | 5,690 (14) | 9,298 (22) | 344 (0.8) | 999 (2) | 3,485 (8) |

^a^ Higher Silhouette score indicates better clustering.

^b^ Lower Davies-Bouldin Index score indicates better clustering.

# eTable 6. Phenotype clinical characteristics and biomarkers in the training cohort

| **Variables** | **Total** | **Acute Illness Phenotypes** | | | |
| --- | --- | --- | --- | --- | --- |
|  |  | Phenotype A | Phenotype B | Phenotype C | Phenotype D |
| Number of encounters (%) | 41,502 | 7,647 (18) | 13,710 (33) | 12,901 (31) | 7,244 (17) |
| **Preadmission clinical characteristics** |  |  |  |  |  |
| Age, mean (SD), years | 54 (19) | 57 (19)^a,c^ | 53 (19)^a,b^ | 51 (19) | 57 (17)^a^ |
| Female sex, n (%) | 22,745 (55) | 3,963 (52)^a,c^ | 7,595 (55)^a,b^ | 7,391 (57) | 3,796 (52)^a^ |
| Race, n (%) |  |  |  |  |  |
| White | 29,076 (70) | 5,203 (68)^a,b^ | 9,421 (69)^b^ | 9,021 (70) | 5,431 (75)^a^ |
| African American | 9,634 (23) | 2,036 (27)^a,b,c^ | 3,411 (25)^a,b^ | 2,930 (23) | 1,257 (17)^a^ |
| Primary insurance, n (%) |  |  |  |  |  |
| Private | 9,591 (23) | 1,323 (17)^a,b,c^ | 2,917 (21)^a,b^ | 3,314 (26) | 2,037 (28)^a^ |
| Medicare | 18,499 (45) | 3,839 (50)^a,b,c^ | 6,120 (45)^a,b^ | 5,158 (40) | 3,382 (47)^a^ |
| Medicaid | 9,231 (22) | 1,641 (21)^a,b,c^ | 3,213 (23)^b^ | 3,104 (24) | 1,273 (18)^a^ |
| Uninsured | 4,181 (10) | 844 (11)^b^ | 1,460 (11)^b^ | 1,325 (10) | 552 (8)^a^ |
| Residing neighborhood characteristics |  |  |  |  |  |
| Proportion of African Americans (%), mean (SD) | 18.7 (17.5) | 19.6 (17.8)^a,b^ | 19.3 (17.8)^a,b^ | 18.6 (17.5) | 17.2 (16.1)^a^ |
| Proportion below poverty (%), mean (SD) | 22.7 (10.1) | 23.8 (10.1)^a,b,c^ | 23.1 (10.1)^a,b^ | 22.5 (10.0) | 21.2 (9.8)^a^ |
| Distance from hospital (mile), median (IQR) | 18 (3, 34) | 14 (3, 27)^a,b,c^ | 14 (3, 32)^a,b^ | 18 (3, 36) | 23 (9, 40)^a^ |
| **Comorbidities** |  |  |  |  |  |
| Hypertension, n (%) | 21,639 (52) | 4,129 (54)^a,b^ | 7,205 (53)^b^ | 6,704 (52) | 3,601 (50)^a^ |
| Cardiovascular disease, n (%)^d^ | 12,058 (29) | 2,413 (32)^a,b,c^ | 3,991 (29)^b^ | 3,682 (29) | 1,972 (27) |
| Diabetes mellitus, n (%) | 10,111 (24) | 1,972 (26)^a,b^ | 3,370 (25) | 3,100 (24) | 1,669 (23) |
| Chronic kidney disease, n (%) | 6,518 (16) | 1,450 (19)^a,b^ | 2,467 (18)^a,b^ | 1,802 (14) | 799 (11)^a^ |
| **Admission characteristics of patients** |  |  |  |  |  |
| Emergent admission, n (%) | 30,177 (73) | 7,257 (95)^a,b,c^ | 11,764 (86)^a,b^ | 8,064 (63) | 3,092 (43)^a^ |
| Transfer from another hospital, n (%) | 7,115 (17) | 1,986 (26)^a,b,c^ | 3,014 (22)^a,b^ | 1,087 (8) | 1,028 (14)^a^ |
| **Primary admission diagnostic groups** |  |  |  |  |  |
| Diseases of the circulatory system | 7,719 (19) | 1,934 (25)^a,b,c^ | 2,425 (18)^a,b^ | 1,834 (14) | 1,526 (21)^a^ |
| Respiratory and infectious diseases | 3,306 (8) | 961 (13)^a,b,c^ | 1,161 (8)^a,b^ | 705 (5) | 479 (7)^a^ |
| Complications of pregnancy and childbirth | 3,148 (8) | 391 (5)^a,c^ | 1,147 (8)^a,b^ | 1,248 (10) | 362 (5)^a^ |
| Diseases of the digestive/genitourinary systems | 5,184 (12) | 789 (10)^a,c^ | 1686 (12)^a^ | 1,876 (15) | 833 (11)^a^ |
| Diseases of the musculoskeletal/connective tissue and skin | 3,651 (9) | 317 (4)^a,b,c^ | 1042 (8)^a,b^ | 1,222 (9) | 1,070 (15)^a^ |
| Neoplasms | 2,743 (7) | 93 (1)^a,b,c^ | 665 (5)^a,b^ | 1,138 (9) | 847 (12)^a^ |
| **Clinical biomarkers and interventions within 24 hours of admission** |  |  |  |  |  |
| Surgical procedure on admission day, n (%) | 8,644 (21) | 272 (4)^a,b,c^ | 813 (6)^a,b^ | 3,466 (27) | 4,093 (57)^a^ |
| ICU/IMC admission within first 24 hours, n (%) | 9,426 (23) | 2,372 (31)^a,b,c^ | 1,979 (14)^b^ | 1,921 (15) | 3,154 (44)^a^ |
| **Cardiovascular system** |  |  |  |  |  |
| Hypotension (MAP < 60 mmHg) at any time, n (%) | 14,470 (35) | 2,234 (29)^a,b,c^ | 2,903 (21)^a,b^ | 4,445 (34) | 4,888 (67)^a^ |
| Duration, median (IQR), minutes | 57 (15, 168) | 86 (30, 224)^a,b^ | 92 (30, 233)^a,b^ | 33 (10, 120) | 37 (10, 129) |
| Vasopressors used, n (%) | 7,531 (18) | 421 (6)^a,b,c^ | 641 (5)^a,b^ | 2633 (20) | 3836 (53)^a^ |
| Out of operating room | 1,403 (3) | 242 (3)^a,b,c^ | 146 (1)^a,b^ | 229 (2) | 786 (11)^a^ |
| Hypertension (SBP > 160 mmHg) at any time, n (%) | 14,838 (36) | 2,923 (38)^a,b,c^ | 3,684 (27)^a,b^ | 4,272 (33) | 3,959 (55)^a^ |
| Duration, median (IQR), minutes | 120 (27, 356) | 174 (52, 445)^a,b,c^ | 214 (73, 477)^a,b^ | 114 (19, 336) | 44 (9, 165)^a^ |
| Troponin, tested, n (%) | 14,616 (35) | 4,502 (59)^a,b,c^ | 4,862 (35)^a,b^ | 3,055 (24) | 2,197 (30)^a^ |
| Abnormal result among tested, n (%) | 3,398 (23) | 1,109 (25)^a,b,c^ | 884 (18)^b^ | 585 (19) | 820 (37)^a^ |
| **Respiratory system** |  |  |  |  |  |
| Highest administered FiO2, median (IQR), % | 0.21 (0.21, 0.40) | 0.21 (0.21, 0.29)^a,b,c^ | 0.21 (0.21, 0.28)^a,b^ | 0.21 (0.21, 0.40) | 0.40 (0.29, 0.40)^a^ |
| Room air only, n (%) | 23,963 (58) | 4,615 (60)^b,c^ | 10,130 (74)^a,b^ | 7,874 (61) | 1,344 (19)^a^ |
| 0.22 – 0.40, n (%) | 14,790 (36) | 2,496 (33)^a,b,c^ | 3,252 (24)^a,b^ | 4,484 (35) | 4,558 (63)^a^ |
| > 0.40, n (%) | 2,749 (7) | 536 (7)^a,b,c^ | 328 (2)^a,b^ | 543 (4) | 1,342 (19)^a^ |
| P_a_O2/FiO_2_, tested with arterial blood gas, n (%) | 6,113 (15) | 1,352 (18)^a,b,c^ | 1,033 (8)^a,b^ | 1,273 (10) | 2,455 (34)^a^ |
| <200 among tested, n (%) | 2,265 (37) | 496 (37)^b,c^ | 301 (29)^b^ | 430 (34) | 1,038 (42)^a^ |
| Mechanical ventilation, n (%) | 2,123 (5) | 434 (6)^a,b,c^ | 191 (1)^a,b^ | 314 (2) | 1,184 (16)^a^ |
| **Kidney and acid-base status** |  |  |  |  |  |
| Preadmission estimated glomerular filtration rate^e^ (mL/min per 1.73 m^2^), median (IQR) | 95 (78, 111) | 93 (74, 109)^a,c^ | 96 (77, 111)^a,b^ | 97 (80, 113) | 93 (79, 106)^a^ |
| Highest /reference creatinine^e^ ratio, mean (SD) | 1.24 (0.66) | 1.30 (0.70)^a,b,c^ | 1.22 (0.59)^a,b^ | 1.20 (0.67) | 1.28 (0.74)^a^ |
| Renal replacement therapy, n (%) | 641 (2) | 160 (2)^a,c^ | 168 (1)^b^ | 175 (1) | 138 (2)^a^ |
| Highest anion gap, median (IQR), mmol/L | 14 (12, 17) | 15 (12, 18)^a,c^ | 14 (12, 16)^b^ | 14 (11, 16) | 15 (12, 18)^a^ |
| Arterial blood gas tested, n (%) | 6,115 (15) | 1,353 (18)^a,b,c^ | 1,033 (8)^a,b^ | 1,274 (10) | 2,455 (34)^a^ |
| pH < 7.3 among tested, n (%) | 1437 (23) | 298 (22)^b,c^ | 160 (15)^b^ | 242 (19) | 737 (30)^a^ |
| Highest base deficit among tested, mean (SD), mmol/L | 4.8 (4.7) | 5.4 (5.0)^a,c^ | 4.5 (4.6) | 4.4 (4.3) | 4.8 (4.6) |
| Lactate, tested, n (%) | 15,447 (37) | 3,935 (51)^a,b,c^ | 4,308 (31)^a,b^ | 3,706 (29) | 3,498 (48)^a^ |
| 2 – 4 mmol/L among tested, n (%) | 3,739 (24) | 978 (25)^c^ | 956 (22)^b^ | 870 (23) | 935 (27)^a^ |
| > 4 mmol/L among tested, n (%) | 1,374 (9) | 331 (8)^a,b,c^ | 207 (5)^b^ | 216 (6) | 620 (18)^a^ |
| **Inflammation** |  |  |  |  |  |
| Highest white blood cell count, median (IQR), x10^9^/L | 9 (7, 13) | 9 (7, 13)^b,c^ | 9 (6, 12)^a,b^ | 9 (7, 12) | 11 (8, 15)^a^ |
| Highest premature neutrophils (bands), median (IQR), % | 10 (4, 20) | 12 (4, 22)^a,b,c^ | 7 (3, 15)^b^ | 9 (3, 18) | 15 (7, 26)^a^ |
| Lowest lymphocytes, median (IQR), % | 16 (9, 24) | 15 (8, 24)^a,b,c^ | 16 (10, 25)^b^ | 17 (10, 26) | 10 (6, 18)^a^ |
| C-reactive protein, tested, n (%) | 5,862 (14) | 1,246 (16)^a,b^ | 2,396 (17)^a,b^ | 1,759 (14) | 461 (6)^a^ |
| Highest C-reactive protein, median (IQR), mg/L | 18 (5, 77) | 20 (5, 89)^a,b^ | 18 (5, 73)^a,b^ | 15 (4, 70) | 39 (7, 112)^a^ |
| Erythrocyte sedimentation rate, tested, n (%) | 3,903 (9) | 775 (10)^b,c^ | 1,591 (12)^a,b^ | 1,253 (10) | 284 (4)^a^ |
| Highest erythrocyte sedimentation rate, median (IQR), mm/h | 40 (19, 73) | 42 (18, 72) | 41 (20, 75)^b^ | 39 (19, 71) | 32 (15, 66) |
| Highest temperature, mean (SD), Celsius | 37.7 (0.6) | 37.7 (0.6)^b,c^ | 37.6 (0.6)^a,b^ | 37.7 (0.6) | 37.9 (0.6)^a^ |
| 38 - 39, n (%) | 8,633 (21) | 1,519 (20)^b,c^ | 2,238 (16)^a,b^ | 2,486 (19) | 2,390 (33)^a^ |
| > 39, n (%) | 1,548 (4) | 354 (5)^a,c^ | 453 (3)^b^ | 368 (3) | 373 (5)^a^ |
| Lowest temperature, mean (SD), Celsius | 36.7 (1.0) | 36.7 (0.8)^b,c^ | 36.8 (0.7)^a,b^ | 36.7 (0.7) | 36.3 (1.7)^a^ |
| **Hematologic** |  |  |  |  |  |
| Lowest hemoglobin, mean (SD), g/dL | 11.5 (2.3) | 11.6 (2.4)^b^ | 11.7 (2.3)^b^ | 11.6 (2.2) | 10.9 (2.3)^a^ |
| Highest RDW, mean (SD), % | 15.5 (2.1) | 15.6 (2.1)^a,b^ | 15.6 (2.3)^a,b^ | 15.4 (2.1) | 15.3 (1.9) |
| Lowest platelets, median (IQR), x10^9^/L | 210 (161, 269) | 209 (160, 271)^a,b,c^ | 216 (165, 277)^b^ | 214 (165, 270) | 195 (150, 247)^a^ |
| Platelets < 200, n (%), x10^9^/L | 16,707 (40) | 3,289 (43)^a,b,c^ | 5,347 (39)^a,b^ | 4,769 (37) | 3,302 (46)^a^ |
| < 100 | 2,643 (16) | 526 (7) | 910 (7)^a^ | 715 (6) | 492 (7) |
| 100 - 200 | 14,064 (84) | 2,763 (84) | 4,437 (83)^a^ | 4,054 (85) | 2,810 (85) |
| International normalized ratio, tested, n (%) | 20,357 (49) | 4,830 (63)^a,b,c^ | 6,942 (51)^a,b^ | 5,201 (40) | 3,384 (47)^a^ |
| >= 2 | 1,836 (9) | 432 (9) | 666 (10) | 433 (8) | 305 (9) |
| **Neurologic** |  |  |  |  |  |
| Glasgow Coma Scale score, n (%) |  |  |  |  |  |
| Moderate neurologic dysfunction (9 - 12) | 1,708 (4) | 385 (5)^a,b,c^ | 340 (2)^b^ | 284 (2) | 699 (10)^a^ |
| Severe neurologic dysfunction (<= 8) | 1,482 (4) | 359 (5)^a,b,c^ | 144 (1)^a,b^ | 207 (2) | 772 (11)^a^ |
| **Liver and metabolic** |  |  |  |  |  |
| Bilirubin tested, n (%), mg/dL | 21,183 (51) | 4,759 (62)^a,b,c^ | 8,018 (58)^a,b^ | 5,894 (46) | 2,512 (35)^a^ |
| ≥ 2 | 1,427 (7) | 271 (6)^b^ | 542 (7)^b^ | 395 (7) | 219 (9)^a^ |
| Highest glucose, median (IQR), mg/dL | 126 (104, 170) | 127 (105, 175)^a,b,c^ | 120 (101, 161)^b^ | 123 (101, 164) | 144 (116, 187)^a^ |
| Albumin, tested, n (%) | 21,368 (51) | 4,780 (63)^a,b,c^ | 8,070 (59)^a,b^ | 5,951 (46) | 2,567 (35)^a^ |
| < 2.5 | 1,243 (6) | 304 (6)^a,b,c^ | 419 (5)^b^ | 260 (4) | 260 (10)^a^ |
| 2.5 - 3.5 | 6,904 (32) | 1,678 (35)^a,b,c^ | 2,515 (31)^a,b^ | 1,719 (29) | 992 (39)^a^ |

Abbreviation: ICU: intensive care unit; IMC: intermediate care unit; MAP: mean arterial pressure; SBP: systolic blood pressure; RDW: red cell distribution width; SD: standard deviation; IQR: interquartile range.

All p-values were adjusted for multiple comparisons using the Bonferroni method.

^a^ p < 0.05 compared to Phenotype C.

^b^ p < 0.05 compared to Phenotype D.

^c^ p < 0.05 compared to Phenotype B.

^d^ Cardiovascular disease was considered if there was a history of congestive heart failure, coronary artery disease, or peripheral vascular disease.

^e^ Reference glomerular filtration rate and reference creatinine were derived without use of race correction (see eMethods for details).

# eTable 7. Phenotype illness severity, clinical outcomes, and resource use in the training cohort

| **Variables** | **Total** | **Acute Illness Phenotypes** | | | |
| --- | --- | --- | --- | --- | --- |
|  |  | Phenotype A | Phenotype B | Phenotype C | Phenotype D |
| Number of encounters (%) | 41,502 | 7,647 (18) | 13,710 (33) | 12,901 (31) | 7,244 (17) |
| **Acuity scores within 24h of admission** |  |  |  |  |  |
| SOFA score > 6, n (%) | 3,506 (8) | 656 (9)^a,b,c^ | 508 (4)^a,b^ | 768 (6) | 1,574 (22)^a^ |
| Patients in ICU/IMC, SOFA score ≤ 6, n (%) | 6,882 (17) | 1,822 (24)^a,c^ | 1,690 (12)^b^ | 1,514 (12) | 1,856 (26)^a^ |
| Patients in ICU/IMC, SOFA score > 6, n (%) | 2,544 (6) | 550 (7)^a,b,c^ | 289 (2)^a,b^ | 407 (3) | 1,298 (18)^a^ |
| Patients on ward, SOFA score ≤ 6, n (%) | 31,114 (75) | 5,169 (68)^a,b,c^ | 11,512 (84)^a,b^ | 10,619 (82) | 3,814 (53)^a^ |
| Patients on ward, SOFA score > 6, n (%) | 962 (2) | 106 (1)^a,b^ | 219 (2)^a,b^ | 361 (3) | 276 (4)^a^ |
| MEWS score ≥ 5, n (%) | 2,828 (7) | 873 (11)^a,b,c^ | 575 (4)^a,b^ | 387 (3) | 993 (14)^a^ |
| Patients in ICU/IMC, MEWS score ≤ 4, n (%) | 7,235 (17) | 1,703 (22)^a,b,c^ | 1,618 (12)^b^ | 1,643 (13) | 2,271 (31)^a^ |
| Patients in ICU/IMC, MEWS score > 4, n (%) | 2,191 (5) | 669 (9)^a,b,c^ | 361 (3)^b^ | 278 (2) | 883 (12)^a^ |
| Patients on ward, MEWS score ≤ 4, n (%) | 31,439 (76) | 5,071 (66)^a,b,c^ | 11,517 (84)^b^ | 10,871 (84) | 3,980 (55)^a^ |
| Patients on ward, MEWS score > 4, n (%) | 637 (2) | 204 (3)^a,b,c^ | 214 (2)^a^ | 109 (1) | 110 (2)^a^ |
| **Resource use during hospitalization** |  |  |  |  |  |
| Hospital days, median (IQR) | 4 (2, 7) | 4 (2, 7)^a,b,c^ | 4 (2, 7)^a,b^ | 3 (2, 6) | 4 (3, 7)^a^ |
| Surgery at any time, n (%) | 11,634 (28) | 860 (11)^a,b,c^ | 2,006 (15)^a,b^ | 4,452 (35) | 4,316 (60)^a^ |
| Admitted to ICU/IMC^d^, n (%) | 11,121 (27) | 2,700 (35)^a,b,c^ | 2,673 (19)^b^ | 2,446 (19) | 3,302 (46)^a^ |
| Days in ICU/IMC^e^, median (IQR) | 4 (2, 7) | 4 (3, 7)^a^ | 4 (3, 7)^a^ | 4 (2, 6) | 4 (3, 8)^a^ |
| More than 48 hrs in ICU/IMC^e^, n (%) | 8,332 (75) | 2,068 (77)^a^ | 2,008 (75)^a^ | 1,751 (72) | 2,505 (76)^a^ |
| Mechanical ventilation, n (%) | 3,218 (8) | 695 (9)^a,b,c^ | 554 (4)^a,b^ | 628 (5) | 1341 (19)^a^ |
| Hours on mechanical ventilation^f^, median (IQR)^d^ | 35 (14, 113) | 44 (17, 127)^a,b^ | 35 (13, 116) | 25 (11, 101) | 33 (14, 113) |
| More than 48 hrs on mechanical ventilation^f^, n (%) | 1,661 (52) | 403 (58)^a,b^ | 284 (51) | 302 (48) | 672 (50) |
| Renal replacement therapy, n (%) | 1,262 (3) | 314 (4)^a,b,c^ | 396 (3) | 322 (2) | 230 (3)^a^ |
| **Complications** |  |  |  |  |  |
| Acute kidney injury, n (%) | 6905 (17) | 1,598 (21)^a,b,c^ | 2,279 (17)^a,b^ | 1,680 (13) | 1,348 (19)^a^ |
| Community-acquired AKI, n (%) | 3839 (56) | 924 (58)^c^ | 1,200 (53)^b^ | 897 (53) | 818 (61)^a^ |
| Hospital-acquired AKI, n (%) | 3066 (44) | 674 (42)^c^ | 1,079 (47)^b^ | 783 (47) | 530 (39)^a^ |
| Worst AKI staging, n (%) |  |  |  |  |  |
| Stage 1 | 4360 (63) | 961 (60)^a,c^ | 1,479 (65)^b^ | 1,112 (66) | 808 (60)^a^ |
| Stage 2 | 1362 (20) | 346 (22)^a^ | 425 (19) | 300 (18) | 291 (22) |
| Stage 3 | 848 (12) | 206 (13) | 270 (12) | 202 (12) | 170 (13) |
| Stage 3 with RRT | 335 (5) | 85 (5) | 105 (5) | 66 (4) | 79 (6) |
| Venous thromboembolism, n (%) | 1257 (3) | 261 (3)^a,b^ | 481 (4)^a,b^ | 334 (3) | 181 (2) |
| Sepsis, n (%) | 3750 (9) | 1,049 (14)^a,b,c^ | 1,102 (8)^a,b^ | 754 (6) | 845 (12)^a^ |
| Hospital disposition, n (%) |  |  |  |  |  |
| Hospital mortality | 1141 (3) | 294 (4)^a,b,c^ | 278 (2)^a,b^ | 184 (1) | 385 (5)^a^ |
| Another hospital, LTAC, SNF, Hospice | 4475 (11) | 1,140 (15)^a,b,c^ | 1,591 (12)^a^ | 932 (7) | 812 (11)^a^ |
| Home or short-term rehabilitation | 35886 (86) | 6,213 (81)^a,b,c^ | 11,841 (86)^a,b^ | 11,785 (91) | 6,047 (83)^a^ |
| 30-day mortality, n (%) | 1633 (3.9) | 439 (6)^a,c^ | 458 (3)^a,b^ | 278 (2) | 458 (6)^a^ |
| Three-year mortality, n (%) | 8013 (19) | 1,892 (25)^a,b,c^ | 2,861 (21)^a,b^ | 1,975 (15) | 1,285 (18)^a^ |

Abbreviation: SOFA: sequential organ failure assessment; MEWS: modified early warning score; ICU: intensive care unit; IMC: intermediate care unit; IQR: interquartile range.

All p-values were adjusted for multiple comparisons using the Bonferroni method.

^a^ p < 0.05 compared to Phenotype C.

^b^ p < 0.05 compared to Phenotype D.

^c^ p < 0.05 compared to Phenotype B.

^d^ ICU/IMC admission rate was derived from data collected at any point during the hospitalization period. When an encounter had multiple ICU admissions throughout the hospitalization, the admission rate will only be evaluated once.

^e^ The length of ICU stay and prolonged ICU stay (greater than 48 hours) were derived for only patients who were admitted to the ICU or IMC.

^f^ The duration of receiving mechanical ventilation and prolonged mechanical ventilation (greater than 48 hours) were derived for only patients who required mechanical ventilation.

# eTable 8. Frequency distribution of admission diagnoses across phenotypes in the training cohort

| **Variables** | **Level 2 label^*^** | **Level 1 label^*^** | **Phenotype A** | **Phenotype B** | **Phenotype C** | **Phenotype D** | **Total** |
| --- | --- | --- | --- | --- | --- | --- | --- |
| Number of Encounters with codes, n (%) |  |  | 6683 (18.4) | 11908 (32.7) | 11309 (31.1) | 6477 (17.8) | 36377 |
| Top five diagnosis code groups |  |  |  |  |  |  |  |
| Top 1 |  |  | Nonspecific chest pain [102.] | Nonspecific chest pain [102.] | Abdominal pain [251.] | Osteoarthritis [203.] | Nonspecific chest pain [102.] |
| Top 2 |  |  | Other and unspecified lower respiratory disease | Abdominal pain [251.] | Nonspecific chest pain [102.] | Acute cerebrovascular disease [109.] | Abdominal pain [251.] |
| Top 3 |  |  | Septicemia (except in labor) [2.] | Other and unspecified lower respiratory disease | Complication of device; implant or graft [237.] | Complication of device; implant or graft [237.] | Other and unspecified lower respiratory disease |
| Top 4 |  |  | Abdominal pain [251.] | Congestive heart failure; nonhypertensive [108.] | Other and unspecified lower respiratory disease | Septicemia (except in labor) [2.] | Complication of device; implant or graft [237.] |
| Top 5 |  |  | Acute cerebrovascular disease [109.] | Complication of device; implant or graft [237.] | Cellulitis and abscess | Cardiac dysrhythmias [106.] | Septicemia (except in labor) [2.] |
| Diagnosis code group, n (%) |  |  |  |  |  |  |  |
| Nonspecific chest pain [102.] | Diseases of the heart | Diseases of the circulatory system | 636 (9.5) | 843 (7.1) | 478 (4.2) | 130 (2.0) | 2087 (5.7) |
| Abdominal pain [251.] | Symptoms; signs; and ill-defined conditions | Symptoms; signs; and ill-defined conditions and factors influencing health status | 303 (4.5) | 730 (6.1) | 737 (6.5) | 77 (1.2) | 1847 (5.1) |
| Other and unspecified lower respiratory disease | Other lower respiratory disease [133.] | Diseases of the respiratory system | 378 (5.7) | 519 (4.4) | 304 (2.7) | 160 (2.5) | 1361 (3.7) |
| Complication of device; implant or graft [237.] | Complications | Injury and poisoning | 128 (1.9) | 336 (2.8) | 358 (3.2) | 317 (4.9) | 1139 (3.1) |
| Septicemia (except in labor) [2.] | Bacterial infection | Infectious and parasitic diseases | 354 (5.3) | 267 (2.2) | 165 (1.5) | 290 (4.5) | 1076 (3.0) |
| Acute cerebrovascular disease [109.] | Cerebrovascular disease | Diseases of the circulatory system | 285 (4.3) | 212 (1.8) | 85 (0.8) | 349 (5.4) | 931 (2.6) |
| Cardiac dysrhythmias [106.] | Diseases of the heart | Diseases of the circulatory system | 207 (3.1) | 233 (2.0) | 196 (1.7) | 244 (3.8) | 880 (2.4) |
| Congestive heart failure; nonhypertensive [108.] | Diseases of the heart | Diseases of the circulatory system | 206 (3.1) | 399 (3.4) | 191 (1.7) | 58 (0.9) | 854 (2.3) |
| Osteoarthritis [203.] | Non-traumatic joint disorders | Diseases of the musculoskeletal system and connective tissue | 3 (0.0) | 6 (0.1) | 98 (0.9) | 610 (9.4) | 717 (2.0) |
| Complications of surgical procedures or medical care [238.] | Complications | Injury and poisoning | 90 (1.3) | 249 (2.1) | 224 (2.0) | 76 (1.2) | 639 (1.8) |
| Syncope [245.] | Symptoms; signs; and ill-defined conditions | Symptoms; signs; and ill-defined conditions and factors influencing health status | 193 (2.9) | 240 (2.0) | 135 (1.2) | 39 (0.6) | 607 (1.7) |
| Cellulitis and abscess | Skin and subcutaneous tissue infections [197.] | Diseases of the skin and subcutaneous tissue | 70 (1.0) | 261 (2.2) | 256 (2.3) | 15 (0.2) | 602 (1.7) |
| Chemotherapy | Maintenance chemotherapy; radiotherapy [45.] | Neoplasms | 4 (0.1) | 267 (2.2) | 252 (2.2) | 2 (0.0) | 525 (1.4) |
| Other back problems | Spondylosis; intervertebral disc disorders; other back problems [205.] | Diseases of the musculoskeletal system and connective tissue | 56 (0.8) | 192 (1.6) | 214 (1.9) | 60 (0.9) | 522 (1.4) |
| Other complications of pregnancy [181.] | Complications mainly related to pregnancy | Complications of pregnancy; childbirth; and the puerperium | 47 (0.7) | 212 (1.8) | 205 (1.8) | 36 (0.6) | 500 (1.4) |
| Pneumonia (except that caused by tb or std) [122.] | Respiratory infections | Diseases of the respiratory system | 147 (2.2) | 192 (1.6) | 88 (0.8) | 49 (0.8) | 476 (1.3) |
| Nausea and vomiting [250.] | Symptoms; signs; and ill-defined conditions | Symptoms; signs; and ill-defined conditions and factors influencing health status | 89 (1.3) | 202 (1.7) | 153 (1.4) | 14 (0.2) | 458 (1.3) |
| Outcome of delivery (v codes) | Normal pregnancy and/or delivery [196.] | Complications of pregnancy; childbirth; and the puerperium | 58 (0.9) | 159 (1.3) | 174 (1.5) | 56 (0.9) | 447 (1.2) |
| Aortic; peripheral; and visceral artery aneurysms [115.] | Diseases of arteries; arterioles; and capillaries | Diseases of the circulatory system | 81 (1.2) | 53 (0.4) | 97 (0.9) | 196 (3.0) | 427 (1.2) |
| Acute and unspecified renal failure [157.] | Diseases of the urinary system | Diseases of the genitourinary system | 108 (1.6) | 179 (1.5) | 102 (0.9) | 21 (0.3) | 410 (1.1) |
| Obstructive chronic bronchitis | Chronic obstructive pulmonary disease and bronchiectasis [127.] | Diseases of the respiratory system | 140 (2.1) | 135 (1.1) | 76 (0.7) | 58 (0.9) | 409 (1.1) |
| Other and unspecified gastrointestinal disorders | Other gastrointestinal disorders [155.] | Diseases of the digestive system | 50 (0.7) | 123 (1.0) | 164 (1.5) | 63 (1.0) | 400 (1.1) |
| Convulsions | Epilepsy; convulsions [83.] | Diseases of the nervous system and sense organs | 116 (1.7) | 158 (1.3) | 84 (0.7) | 38 (0.6) | 396 (1.1) |

Admission diagnoses that constituted at least 1% of the cohort were listed.

* The classification of diagnosis code was generated based on clinical classification software for ICD-10-CM v2019.119. eTable 9. Phenotype clinical characteristics and biomarkers in the testing cohort

| **Variables** | **Total** | **Acute Illness Phenotypes** | | | |
| --- | --- | --- | --- | --- | --- |
|  |  | Phenotype A | Phenotype B | Phenotype C | Phenotype D |
| Number of encounters (%) | 16,845 | 3,036 (18) | 5,880 (35) | 5,201 (31) | 2,728 (16) |
| **Preadmission clinical characteristics** |  |  |  |  |  |
| Age, mean (SD), years | 55 (19) | 57 (19)^a,b,c^ | 54 (19)^a,b^ | 52 (19) | 58 (17)^a^ |
| Female sex, n (%) | 9,205 (55) | 1,592 (52)^a,c^ | 3,287 (56)^b^ | 2,932 (56) | 1,394 (51)^a^ |
| Race, n (%) |  |  |  |  |  |
| White | 11,854 (70) | 2,096 (69)^b^ | 4,063 (69)^b^ | 3,646 (70) | 2,049 (75)^a^ |
| African American | 3,845 (23) | 780 (26)^a,b^ | 1,415 (24)^b^ | 1,181 (23) | 469 (17)^a^ |
| Primary insurance, n (%) |  |  |  |  |  |
| Private | 3,935 (23) | 557 (18)^a,b,c^ | 1281 (22)^a,b^ | 1339 (26) | 758 (28) |
| Medicare | 7,845 (47) | 1,574 (52)^a,c^ | 2,717 (46)^a,b^ | 2,196 (42) | 1,358 (50)^a^ |
| Medicaid | 3,592 (21) | 623 (21)^b^ | 1,330 (23)^b^ | 1,172 (23) | 467 (17)^a^ |
| Uninsured | 1,473 (9) | 282 (9)^b^ | 552 (9)^b^ | 494 (9) | 145 (5)^a^ |
| Residing neighborhood characteristics |  |  |  |  |  |
| Proportion of African Americans (%), mean (SD) | 18.7 (17.4) | 19.7 (17.9)^a,b^ | 19.3 (17.6)^a,b^ | 18.8 (17.8) | 16.4 (15.7)^a^ |
| Proportion below poverty (%), mean (SD) | 22.5 (10.2) | 23.5 (10.3)^a,b,c^ | 22.7 (10.2)^b^ | 22.5 (10.2) | 20.7 (9.9)^a^ |
| Distance from hospital (mile), median (IQR) | 18 (3, 34) | 14 (3, 27)^a,b,c^ | 14 (3, 32)^a,b^ | 18 (3, 34) | 24 (9, 40)^a^ |
| **Comorbidities** |  |  |  |  |  |
| Hypertension, n (%) | 8,468 (50) | 1,510 (50) | 2,966 (50) | 2,625 (50) | 1,367 (50) |
| Cardiovascular disease, n (%)^d^ | 4,702 (28) | 850 (28) | 1,671 (28) | 1,415 (27) | 766 (28) |
| Diabetes mellitus, n (%) | 3,945 (23) | 761 (25) | 1,334 (23) | 1,203 (23) | 647 (24) |
| Chronic kidney disease, n (%) | 2,892 (17) | 638 (21)^b^ | 1,169 (20)^a,b^ | 787 (15) | 298 (11)^a^ |
| **Admission characteristics of patients** |  |  |  |  |  |
| Emergent admission, n (%) | 12,289 (73) | 2,888 (95)^a,b,c^ | 5,048 (86)^a,b^ | 3,353 (64) | 1,000 (37)^a^ |
| Transfer from another hospital, n (%) | 2,859 (17) | 743 (24)^a,b,c^ | 1287 (22)^a,b^ | 464 (9) | 365 (13)^a^ |
| **Primary admission diagnostic groups** |  |  |  |  |  |
| Diseases of the circulatory system | 2,983 (18) | 688 (23)^a,c^ | 989 (17)^a,b^ | 734 (14) | 572 (21)^a^ |
| Respiratory and infectious diseases | 1,525 (9) | 436 (14)^a,b,c^ | 576 (10)^a,b^ | 351 (7) | 162 (6) |
| Complications of pregnancy and childbirth | 1,246 (7) | 151 (5)^a,c^ | 471 (8)^a,b^ | 497 (10) | 127 (5)^a^ |
| Diseases of the digestive/genitourinary systems | 2,147 (13) | 316 (10)^a,c^ | 759 (13)^a^ | 772 (15) | 300 (11)^a^ |
| Diseases of the musculoskeletal/connective tissue and skin | 1,418 (8) | 91 (3)^a,b,c^ | 393 (7)^a,b^ | 480 (9) | 454 (17)^a^ |
| Neoplasms | 1,074 (6) | 54 (2)^a,b,c^ | 306 (5)^a,b^ | 344 (7) | 370 (14)^a^ |
| **Clinical biomarkers and interventions within 24 hours of admission** |  |  |  |  |  |
| Surgical procedure on admission day, n (%) | 3,551 (21) | 109 (4)^a,b,c^ | 329 (6)^a,b^ | 1398 (27) | 1,715 (63)^a^ |
| ICU/IMC admission within first 24 hours, n (%) | 3,838 (23) | 977 (32)^a,b,c^ | 891 (15)^b^ | 827 (16) | 1,143 (42)^a^ |
| **Cardiovascular system** |  |  |  |  |  |
| Hypotension (MAP < 60 mmHg) at any time, n (%) | 5,916 (35) | 940 (31)^a,b,c^ | 1,307 (22)^a,b^ | 1,768 (34) | 1,901 (70)^a^ |
| Duration, median (IQR), minutes | 60 (15, 167) | 90 (35, 240)^a,b^ | 83 (30, 222)^a,b^ | 36 (11, 129) | 33 (10, 120) |
| Vasopressors used, n (%) | 3,166 (19) | 185 (6)^a,b^ | 286 (5)^a,b^ | 1,088 (21) | 1,607 (59)^a^ |
| Out of operating room | 568 (3) | 106 (3)^a,b,c^ | 61 (1)^a,b^ | 107 (2) | 294 (11)^a^ |
| Hypertension (SBP > 160 mmHg) at any time, n (%) | 6,207 (37) | 1,144 (38)^b,c^ | 1,643 (28)^a,b^ | 1,820 (35) | 1,600 (59)^a^ |
| Duration, median (IQR), minutes | 120 (26, 352) | 161 (45, 440)^a,b,c^ | 207 (65, 486)^a,b^ | 111 (18, 345) | 45 (9, 164)^a^ |
| Troponin, tested, n (%) | 5,905 (35) | 1,753 (58)^a,b,c^ | 2,174 (37)^a,b^ | 1,258 (24) | 720 (26) |
| Abnormal result among tested, n (%) | 1,205 (20) | 398 (23)^b,c^ | 357 (16)^b^ | 240 (19) | 210 (29)^a^ |
| **Respiratory system** |  |  |  |  |  |
| Highest administered FiO2, median (IQR), % | 0.21 (0.21, 0.40) | 0.21 (0.21, 0.33)^b,c^ | 0.21 (0.21, 0.28)^a,b^ | 0.21 (0.21, 0.40) | 0.40 (0.40, 0.40)^a^ |
| Room air only, n (%) | 9,682 (57) | 1,772 (58)^a,b,c^ | 4,269 (73)^a,b^ | 3,205 (62) | 436 (16)^a^ |
| 0.22 – 0.40, n (%) | 6,082 (36) | 1,029 (34)^b,c^ | 1,446 (25)^a,b^ | 1,763 (34) | 1,844 (68)^a^ |
| > 0.40, n (%) | 1,081 (6) | 235 (8)^a,b,c^ | 165 (3)^a,b^ | 233 (4) | 448 (16)^a^ |
| P_a_O2/FiO_2_, tested with arterial blood gas, n (%) | 2,603 (15) | 621 (20)^a,b,c^ | 526 (9)^b^ | 537 (10) | 919 (34)^a^ |
| <200 among tested, n (%) | 964 (37) | 239 (38) | 172 (33)^b^ | 181 (34) | 372 (40) |
| Mechanical ventilation, n (%) | 918 (5) | 207 (7)^a,b,c^ | 101 (2)^a,b^ | 154 (3) | 456 (17)^a^ |
| **Kidney and acid-base status** |  |  |  |  |  |
| Preadmission estimated glomerular filtration rate^e^ (mL/min per 1.73 m^2^), median (IQR) | 94 (76, 110) | 92 (72, 107)^a,c^ | 94 (76, 112)^b^ | 96 (79, 112) | 92 (78, 106)^a^ |
| Highest /reference creatinine^e^ ratio, mean (SD) | 1.23 (0.95) | 1.27 (0.70)^a,b,c^ | 1.22 (0.63)^a^ | 1.22 (1.44) | 1.23 (0.63) |
| Renal replacement therapy, n (%) | 268 (2) | 64 (2)^c^ | 75 (1) | 87 (2) | 42 (2) |
| Highest anion gap, median (IQR), mmol/L | 14 (12, 17) | 15 (13, 18)^a,b,c^ | 14 (12, 16)^a,b^ | 14 (12, 17) | 15 (12, 18)^a^ |
| Arterial blood gas tested, n (%) | 2,606 (15) | 622 (20)^a,b,c^ | 526 (9)^b^ | 538 (10) | 920 (34)^a^ |
| pH < 7.3 among tested, n (%) | 586 (22) | 98 (16)^a,b^ | 86 (16)^a,b^ | 125 (23) | 277 (30)^a^ |
| Highest base deficit among tested, mean (SD), mmol/L | 4.6 (4.8) | 4.3 (4.5) | 4.8 (5.5) | 4.4 (4.3) | 4.9 (4.9) |
| Lactate, tested, n (%) | 6,142 (36) | 1,559 (51)^a,b,c^ | 1,826 (31)^a,b^ | 1,486 (29) | 1,271 (47)^a^ |
| 2 – 4 mmol/L among tested, n (%) | 1,480 (24) | 425 (27)^a,c^ | 415 (23) | 308 (21) | 332 (26)^a^ |
| > 4 mmol/L among tested, n (%) | 580 (9) | 154 (10)^b,c^ | 93 (5)^a,b^ | 113 (8) | 220 (17)^a^ |
| **Inflammation** |  |  |  |  |  |
| Highest white blood cell count, median (IQR), x10^9^/L | 9 (7, 12) | 9 (7, 13)^a,b,c^ | 9 (6, 12)^a,b^ | 9 (7, 12) | 11 (8, 14)^a^ |
| Highest premature neutrophils (bands), median (IQR), % | 9 (4, 18) | 9 (5, 17)^b,c^ | 7 (2, 15)^b^ | 9 (4, 20) | 15 (7, 26)^a^ |
| Lowest lymphocytes, median (IQR), % | 16 (9, 24) | 14 (8, 23)^a,b,c^ | 16 (9, 25)^a,b^ | 17 (10, 26) | 10 (6, 18)^a^ |
| C-reactive protein, tested, n (%) | 2,255 (13) | 514 (17)^a,b^ | 914 (16)^a,b^ | 650 (12) | 177 (6)^a^ |
| Highest C-reactive protein, median (IQR), mg/L | 28 (5, 93) | 34 (7, 99)^b^ | 20 (5, 86)^b^ | 17 (4, 77) | 66 (12, 160)^a^ |
| Erythrocyte sedimentation rate, tested, n (%) | 1,263 (7) | 255 (8)^b,c^ | 549 (9)^a,b^ | 398 (8) | 61 (2)^a^ |
| Highest erythrocyte sedimentation rate, median (IQR), mm/h | 41 (20, 73) | 39 (19, 67) | 42 (22, 74) | 39 (19, 75) | 36 (21, 57) |
| Highest temperature, mean (SD), Celsius | 37.7 (0.6) | 37.8 (0.7)^b,c^ | 37.7 (0.6)^a,b^ | 37.7 (0.5) | 37.9 (0.6)^a^ |
| 38 - 39, n (%) | 3,578 (21) | 609 (20)^b,c^ | 1,010 (17)^a,b^ | 1,051 (20) | 908 (33)^a^ |
| > 39, n (%) | 627 (4) | 164 (5)^a,c^ | 207 (4)^a^ | 127 (2) | 129 (5)^a^ |
| Lowest temperature, mean (SD), Celsius | 36.8 (0.9) | 36.8 (0.9)^b^ | 36.8 (0.6)^b^ | 36.8 (0.7) | 36.4 (1.5)^a^ |
| **Hematologic** |  |  |  |  |  |
| Lowest hemoglobin, mean (SD), g/dL | 11.2 (2.3) | 11.4 (2.4)^b^ | 11.3 (2.3)^b^ | 11.4 (2.2) | 10.7 (2.2)^a^ |
| Highest RDW, mean (SD), % | 15.3 (2.1) | 15.4 (2.1)^a,b^ | 15.4 (2.2)^a,b^ | 15.2 (1.9) | 15.1 (1.8) |
| Lowest platelets, median (IQR), x10^9^/L | 207 (160, 266) | 208 (159, 265)^b,c^ | 213 (164, 279)^a^ | 208 (164, 266) | 192 (149, 242)^a^ |
| Platelets < 200, n (%), x10^9^/L | 6,882 (41) | 1,324 (44)^a,b,c^ | 2,329 (40)^a,b^ | 1,992 (38) | 1,237 (45)^a^ |
| < 100 | 1,008 (15) | 204 (15)^a^ | 406 (17)^a,b^ | 238 (12) | 160 (13) |
| 100 - 200 | 5,874 (85) | 1,120 (85)^a^ | 1,923 (83)^a,b^ | 1,754 (88) | 1,077 (87) |
| International normalized ratio, tested, n (%) | 7,070 (42) | 1,579 (52)^a,b,c^ | 2,575 (44)^a^ | 1,778 (34) | 1,138 (42)^a^ |
| >= 2 | 698 (10) | 163 (10) | 276 (11) | 158 (9) | 101 (9) |
| **Neurologic** |  |  |  |  |  |
| Glasgow Coma Scale score, n (%) |  |  |  |  |  |
| Moderate neurologic dysfunction (9 - 12) | 730 (4) | 172 (6)^a,b,c^ | 131 (2)^b^ | 148 (3) | 279 (10)^a^ |
| Severe neurologic dysfunction (<= 8) | 593 (4) | 161 (5)^a,b,c^ | 69 (1)^b^ | 77 (1) | 286 (10)^a^ |
| **Liver and metabolic** |  |  |  |  |  |
| Bilirubin tested, n (%), mg/dL | 7,931 (47) | 1,830 (60)^a,b,c^ | 3,186 (54)^a,b^ | 2,104 (40) | 811 (30)^a^ |
| ≥ 2 | 496 (6) | 113 (6) | 196 (6) | 119 (6) | 68 (8) |
| Highest glucose, median (IQR), mg/dL | 128 (104, 172) | 131 (107, 181)^a,b,c^ | 122 (102, 164)^b^ | 123 (101, 165) | 143 (117, 185)^a^ |
| Albumin, tested, n (%) | 8,023 (48) | 1,838 (61)^a,b,c^ | 3,209 (55)^a,b^ | 2,138 (41) | 838 (31)^a^ |
| < 2.5 | 445 (6) | 117 (6)^a,b^ | 160 (5)^b^ | 85 (4) | 83 (10)^a^ |
| 2.5 - 3.5 | 2,549 (32) | 639 (35)^a,c^ | 979 (31)^b^ | 599 (28) | 332 (40)^a^ |

Abbreviation: ICU: intensive care unit; IMC: intermediate care unit; MAP: mean arterial pressure; SBP: systolic blood pressure; RDW: red cell distribution width; SD: standard deviation; IQR: interquartile range.

All p-values were adjusted for multiple comparisons using the Bonferroni method.

^a^ p < 0.05 compared to Phenotype C.

^b^ p < 0.05 compared to Phenotype D.

^c^ p < 0.05 compared to Phenotype B.

^d^ Cardiovascular disease was considered if there was a history of congestive heart failure, coronary artery disease, or peripheral vascular disease.

^e^ Reference glomerular filtration rate and reference creatinine were derived without use of race correction (see eMethods for details).

# eTable 10. Phenotype illness severity, clinical outcomes, and resource use in the testing cohort

| **Variables** | **Total** | **Acute Illness Phenotypes** | | | |
| --- | --- | --- | --- | --- | --- |
|  |  | Phenotype A | Phenotype B | Phenotype C | Phenotype D |
| Number of encounters (%) | 16,845 | 3,036 (18) | 5,880 (35) | 5,201 (31) | 2,728 (16) |
| **Acuity scores within 24h of admission** |  |  |  |  |  |
| SOFA score > 6, n (%) | 1,454 (9) | 286 (9)^a,b,c^ | 244 (4)^a,b^ | 336 (6) | 588 (22)^a^ |
| Patients in ICU/IMC, SOFA score ≤ 6, n (%) | 2,800 (17) | 741 (24)^a,c^ | 742 (13)^b^ | 631 (12) | 686 (25)^a^ |
| Patients in ICU/IMC, SOFA score > 6, n (%) | 1,038 (6) | 236 (8)^a,b,c^ | 149 (3)^a,b^ | 196 (4) | 457 (17)^a^ |
| Patients on ward, SOFA score ≤ 6, n (%) | 12,591 (75) | 2,009 (66)^a,b,c^ | 4,894 (83)^b^ | 4,234 (81) | 1,454 (53)^a^ |
| Patients on ward, SOFA score > 6, n (%) | 416 (2) | 50 (2)^a,b^ | 95 (2)^a,b^ | 140 (3) | 131 (5)^a^ |
| MEWS score ≥ 5, n (%) | 1,090 (6) | 358 (12)^a,c^ | 271 (5)^a,b^ | 175 (3) | 286 (10)^a^ |
| Patients in ICU/IMC, MEWS score ≤ 4, n (%) | 3,040 (18) | 722 (24)^a,b,c^ | 728 (12)^b^ | 702 (13) | 888 (33)^a^ |
| Patients in ICU/IMC, MEWS score > 4, n (%) | 798 (5) | 255 (8)^a,c^ | 163 (3)^b^ | 125 (2) | 255 (9)^a^ |
| Patients on ward, MEWS score ≤ 4, n (%) | 12,715 (75) | 1,956 (64)^a,b,c^ | 4,881 (83)^b^ | 4,324 (83) | 1,554 (57)^a^ |
| Patients on ward, MEWS score > 4, n (%) | 292 (2) | 103 (3)^a,b,c^ | 108 (2)^a^ | 50 (1) | 31 (1) |
| **Resource use during hospitalization** |  |  |  |  |  |
| Hospital days, median (IQR) | 4 (2, 7) | 4 (2, 7)^a,c^ | 4 (2, 7)^a,b^ | 3 (2, 6) | 4 (3, 7)^a^ |
| Surgery at any time, n (%) | 4,718 (28) | 338 (11)^a,b,c^ | 856 (15)^a,b^ | 1,728 (33) | 1,796 (66)^a^ |
| Admitted to ICU/IMC^d^, n (%) | 4,616 (27) | 1,130 (37)^a,b,c^ | 1,233 (21)^b^ | 1,055 (20) | 1,198 (44)^a^ |
| Days in ICU/IMC^e^, median (IQR) | 4 (2, 7) | 4 (2, 7) | 4 (2, 7)^b^ | 4 (2, 6) | 4 (3, 7)^a^ |
| More than 48 hrs in ICU/IMC^e^, n (%) | 3,401 (74) | 821 (73) | 906 (73) | 756 (72) | 918 (77)^a^ |
| Mechanical ventilation, n (%) | 1,349 (8) | 307 (10)^a,b,c^ | 258 (4)^b^ | 268 (5) | 516 (19)^a^ |
| Hours on mechanical ventilation^f^, median (IQR) | 28 (10, 92) | 31 (14, 90) | 29 (9, 92) | 20 (7, 69) | 29 (12, 98) |
| More than 48 hrs on mechanical ventilation^f^, n (%) | 633 (47) | 151 (49) | 128 (50) | 114 (43) | 240 (47) |
| Renal replacement therapy, n (%) | 530 (3) | 127 (4)^c^ | 160 (3) | 161 (3) | 82 (3) |
| **Complications** |  |  |  |  |  |
| Acute kidney injury, n (%) | 2,741 (16) | 636 (21)^a,b,c^ | 970 (16)^a^ | 670 (13) | 465 (17)^a^ |
| Community-acquired AKI, n (%) | 1,565 (57) | 366 (58) | 521 (54)^b^ | 395 (59) | 283 (61) |
| Hospital-acquired AKI, n (%) | 1,176 (43) | 270 (42) | 449 (46)^b^ | 275 (41) | 182 (39) |
| Worst AKI staging, n (%) |  |  |  |  |  |
| Stage 1 | 1,798 (66) | 401 (63) | 654 (67) | 455 (68) | 288 (62) |
| Stage 2 | 512 (19) | 127 (20) | 169 (17) | 131 (20) | 85 (18) |
| Stage 3 | 300 (11) | 78 (12) | 109 (11) | 57 (9) | 56 (12) |
| Stage 3 with RRT | 131 (5) | 30 (5) | 38 (4)^b^ | 27 (4) | 36 (8) |
| Venous thromboembolism, n (%) | 937 (6) | 203 (7)^a,b^ | 364 (6)^a,b^ | 256 (5) | 114 (4) |
| Sepsis, n (%) | 1,913 (11) | 550 (18)^a,b,c^ | 661 (11)^a^ | 400 (8) | 302 (11)^a^ |
| Hospital disposition, n (%) |  |  |  |  |  |
| Hospital mortality | 513 (3) | 130 (4)^a,c^ | 149 (3)^a,b^ | 75 (1) | 159 (6)^a^ |
| Another hospital, LTAC, SNF, Hospice | 1,946 (12) | 464 (15)^a,b,c^ | 730 (12)^a^ | 435 (8) | 317 (12)^a^ |
| Home or short-term rehabilitation | 14,386 (85) | 2,442 (80)^a,c^ | 5,001 (85)^a,b^ | 4,691 (90) | 2,252 (83)^a^ |
| 30-day mortality, n (%) | 705 (4) | 181 (6)^a,c^ | 221 (4)^a,b^ | 123 (2) | 180 (7)^a^ |
| Three-year mortality, n (%) | 3,324 (20) | 781 (26)^a,b,c^ | 1,228 (21)^a,b^ | 815 (16) | 500 (18)^a^ |

Abbreviation: SOFA: sequential organ failure assessment; MEWS: modified early warning score; ICU: intensive care unit; IMC: intermediate care unit; IQR: interquartile range.

All p-values were adjusted for multiple comparisons using the Bonferroni method.

^a^ p < 0.05 compared to Phenotype C.

^b^ p < 0.05 compared to Phenotype D.

^c^ p < 0.05 compared to Phenotype B.

^d^ ICU/IMC admission rate was derived from data collected at any point during the hospitalization period. When an encounter had multiple ICU admissions throughout the hospitalization, the admission rate will only be evaluated once.

^e^ The length of ICU stay and prolonged ICU stay (greater than 48 hours) were derived for only patients who were admitted to the ICU or IMC.

^f^ The duration of receiving mechanical ventilation and prolonged mechanical ventilation (greater than 48 hours) were derived for only patients who required mechanical ventilation.

# eTable 11. Frequency distribution of admission diagnoses across phenotypes in the testing cohort

| **Variables** | **Level 2 label^*^** | **Level 1 label^*^** | **Phenotype A** | **Phenotype B** | **Phenotype C** | **Phenotype D** | **Total** |
| --- | --- | --- | --- | --- | --- | --- | --- |
| Number of Encounters with codes, n (%) |  |  | 2727 (18.1) | 5208 (34.6) | 4627 (30.7) | 2491 (16.5) | 15053 |
| Top five diagnosis code groups |  |  |  |  |  |  |  |
| Top 1 |  |  | Other and unspecified lower respiratory disease | Nonspecific chest pain [102.] | Abdominal pain [251.] | Osteoarthritis [203.] | Nonspecific chest pain [102.] |
| Top 2 |  |  | Septicemia (except in labor) [2.] | Other and unspecified lower respiratory disease | Nonspecific chest pain [102.] | Acute cerebrovascular disease [109.] | Other and unspecified lower respiratory disease |
| Top 3 |  |  | Nonspecific chest pain [102.] | Abdominal pain [251.] | Complication of device; implant or graft [237.] | Complication of device; implant or graft [237.] | Septicemia (except in labor) [2.] |
| Top 4 |  |  | Acute cerebrovascular disease [109.] | Septicemia (except in labor) [2.] | Other and unspecified lower respiratory disease | Septicemia (except in labor) [2.] | Abdominal pain [251.] |
| Top 5 |  |  | Abdominal pain [251.] | Complication of device; implant or graft [237.] | Other complications of pregnancy [181.] | Aortic; peripheral; and visceral artery aneurysms [115.] | Complication of device; implant or graft [237.] |
| Diagnosis code group, n (%) |  |  |  |  |  |  |  |
| Nonspecific chest pain [102.] | Diseases of the heart | Diseases of the circulatory system | 208 (7.6) | 358 (6.9) | 183 (4.0) | 32 (1.3) | 781 (5.2) |
| Other and unspecified lower respiratory disease | Other lower respiratory disease [133.] | Diseases of the respiratory system | 228 (8.4) | 282 (5.4) | 162 (3.5) | 71 (2.9) | 743 (4.9) |
| Septicemia (except in labor) [2.] | Bacterial infection | Infectious and parasitic diseases | 227 (8.3) | 193 (3.7) | 109 (2.4) | 114 (4.6) | 643 (4.3) |
| Abdominal pain [251.] | Symptoms; signs; and ill-defined conditions | Symptoms; signs; and ill-defined conditions and factors influencing health status | 93 (3.4) | 257 (4.9) | 208 (4.5) | 4 (0.2) | 562 (3.7) |
| Complication of device; implant or graft [237.] | Complications | Injury and poisoning | 56 (2.1) | 160 (3.1) | 177 (3.8) | 143 (5.7) | 536 (3.6) |
| Acute cerebrovascular disease [109.] | Cerebrovascular disease | Diseases of the circulatory system | 97 (3.6) | 64 (1.2) | 46 (1.0) | 162 (6.5) | 369 (2.5) |
| Cardiac dysrhythmias [106.] | Diseases of the heart | Diseases of the circulatory system | 76 (2.8) | 115 (2.2) | 71 (1.5) | 65 (2.6) | 327 (2.2) |
| Osteoarthritis [203.] | Non-traumatic joint disorders | Diseases of the musculoskeletal system and connective tissue | 3 (0.1) | 3 (0.1) | 40 (0.9) | 264 (10.6) | 310 (2.1) |
| Other complications of pregnancy [181.] | Complications mainly related to pregnancy | Complications of pregnancy; childbirth; and the puerperium | 20 (0.7) | 130 (2.5) | 112 (2.4) | 27 (1.1) | 289 (1.9) |
| Congestive heart failure; nonhypertensive [108.] | Diseases of the heart | Diseases of the circulatory system | 76 (2.8) | 103 (2.0) | 55 (1.2) | 12 (0.5) | 246 (1.6) |
| Cellulitis and abscess | Skin and subcutaneous tissue infections [197.] | Diseases of the skin and subcutaneous tissue | 24 (0.9) | 110 (2.1) | 102 (2.2) | 7 (0.3) | 243 (1.6) |
| Complications of surgical procedures or medical care [238.] | Complications | Injury and poisoning | 38 (1.4) | 93 (1.8) | 66 (1.4) | 30 (1.2) | 227 (1.5) |
| Other fractures [231.] | Fractures | Injury and poisoning | 42 (1.5) | 69 (1.3) | 78 (1.7) | 17 (0.7) | 206 (1.4) |
| Syncope [245.] | Symptoms; signs; and ill-defined conditions | Symptoms; signs; and ill-defined conditions and factors influencing health status | 55 (2.0) | 102 (2.0) | 42 (0.9) | 6 (0.2) | 205 (1.4) |
| Hypertension with complications and secondary hypertension [99.] | Hypertension | Diseases of the circulatory system | 54 (2.0) | 49 (0.9) | 58 (1.3) | 37 (1.5) | 198 (1.3) |
| Other back problems | Spondylosis; intervertebral disc disorders; other back problems [205.] | Diseases of the musculoskeletal system and connective tissue | 14 (0.5) | 66 (1.3) | 92 (2.0) | 20 (0.8) | 192 (1.3) |
| Chemotherapy | Maintenance chemotherapy; radiotherapy [45.] | Neoplasms | 0 (0.0) | 123 (2.4) | 65 (1.4) | 2 (0.1) | 190 (1.3) |
| Aortic; peripheral; and visceral artery aneurysms [115.] | Diseases of arteries; arterioles; and capillaries | Diseases of the circulatory system | 27 (1.0) | 32 (0.6) | 38 (0.8) | 93 (3.7) | 190 (1.3) |
| Obstructive chronic bronchitis | Chronic obstructive pulmonary disease and bronchiectasis [127.] | Diseases of the respiratory system | 56 (2.1) | 84 (1.6) | 33 (0.7) | 15 (0.6) | 188 (1.2) |
| Chronic obstructive asthma | Asthma [128.] | Diseases of the respiratory system | 56 (2.1) | 84 (1.6) | 33 (0.7) | 15 (0.6) | 188 (1.2) |
| Hypertension complicating pregnancy; childbirth and the puerperium [183.] | Complications mainly related to pregnancy | Complications of pregnancy; childbirth; and the puerperium | 63 (2.3) | 49 (0.9) | 41 (0.9) | 24 (1.0) | 177 (1.2) |
| Acute and unspecified renal failure [157.] | Diseases of the urinary system | Diseases of the genitourinary system | 37 (1.4) | 78 (1.5) | 43 (0.9) | 10 (0.4) | 168 (1.1) |
| Pneumonia (except that caused by tb or std) [122.] | Respiratory infections | Diseases of the respiratory system | 43 (1.6) | 77 (1.5) | 33 (0.7) | 10 (0.4) | 163 (1.1) |
| Urinary tract infections [159.] | Diseases of the urinary system | Diseases of the genitourinary system | 37 (1.4) | 75 (1.4) | 46 (1.0) | 4 (0.2) | 162 (1.1) |
| Fracture of lower limb [230.] | Fractures | Injury and poisoning | 19 (0.7) | 49 (0.9) | 74 (1.6) | 15 (0.6) | 157 (1.0) |
| Convulsions | Epilepsy; convulsions [83.] | Diseases of the nervous system and sense organs | 41 (1.5) | 62 (1.2) | 37 (0.8) | 11 (0.4) | 151 (1.0) |

Admission diagnoses that constituted at least 1% of the cohort were listed.

^*^ The classification of diagnosis code was generated based on clinical classification software for ICD-10-CM v2019.1^19^

References

1 Gu, Z., Gu, L., Eils, R., Schlesner, M. & Brors, B. circlize Implements and enhances circular visualization in R. *Bioinformatics* **30**, 2811-2812, doi:10.1093/bioinformatics/btu393 (2014).

2 *Alluvial: R Package for Creating Alluvial Diagrams. Version: 0.1-2. Bojanowski M and Edwards R*, <<https://github.com/mbojan/alluvial>> (2016).

3 Pedregosa, F. *et al.* Scikit-learn: Machine learning in Python. *the Journal of machine Learning research* **12**, 2825-2830 (2011).

4 Waskom, M. L. Seaborn: statistical data visualization. *Journal of Open Source Software* **6**, 3021 (2021).

5 Deyo, R. A., Cherkin, D. C. & Ciol, M. A. Adapting a clinical comorbidity index for use with ICD-9-CM administrative databases. *J Clin Epidemiol* **45**, 613-619, doi:10.1016/0895-4356(92)90133-8 (1992).

6 Wald, R. *et al.* Acute renal failure after endovascular vs open repair of abdominal aortic aneurysm. *J Vasc Surg* **43**, 460-466; discussion 466, doi:10.1016/j.jvs.2005.11.053 (2006).

7 Shickel, B. *et al.* DeepSOFA: A Continuous Acuity Score for Critically Ill Patients using Clinically Interpretable Deep Learning. *Sci Rep* **9**, 1879, doi:10.1038/s41598-019-38491-0 (2019).

8 Rice, T. W. *et al.* Comparison of the SpO2/FIO2 ratio and the PaO2/FIO2 ratio in patients with acute lung injury or ARDS. *Chest* **132**, 410-417, doi:10.1378/chest.07-0617 (2007).

9 Selby, N. M., Hill, R. & Fluck, R. J. Standardizing the Early Identification of Acute Kidney Injury: The NHS England National Patient Safety Alert. *Nephron* **131**, 113-117, doi:10.1159/000439146 (2015).

10 Levey, A. S. *et al.* A new equation to estimate glomerular filtration rate. *Ann Intern Med* **150**, 604-612, doi:10.7326/0003-4819-150-9-200905050-00006 (2009).

11 Kidney Disease: Improving Global Outcomes (KDIGO) CKD Work Group. KDIGO 2012 clinical practice guideline for the evaluation and management of chronic kidney disease. *Kidney Int, Suppl.* **3**, 1-150 (2013).

12 Stevens, P. & Levin, A. Kidney Disease: Improving Global Outcomes Chronic Kidney Disease Guideline Development Work Group M. Evaluation and management of chronic kidney disease: synopsis of the kidney disease: improving global outcomes 2012 clinical practice guideline. *Ann Intern Med* **158**, 825-830 (2013).

13 *General equivalence mappings to assist with the conversion ICD-10-CM codes to ICD-9-CM codes*, <<https://www.cms.gov/Medicare/Coding/ICD10/downloads/ICD-10_GEM_fact_sheet.pdf>> (2009).

14 LaPar, D. J. *et al.* Primary payer status affects mortality for major surgical operations. *Ann Surg* **252**, 544-550; discussion 550-541, doi:10.1097/SLA.0b013e3181e8fd75 (2010).

15 Guller, U. *et al.* Laparoscopic versus open appendectomy: outcomes comparison based on a large administrative database. *Ann Surg* **239**, 43-52, doi:10.1097/01.sla.0000103071.35986.c1 (2004).

16 Dombrovskiy, V. Y., Martin, A. A., Sunderram, J. & Paz, H. L. Rapid increase in hospitalization and mortality rates for severe sepsis in the United States: a trend analysis from 1993 to 2003. *Crit Care Med* **35**, 1244-1250, doi:10.1097/01.Ccm.0000261890.41311.E9 (2007).

17 Thottakkara, P. *et al.* Application of Machine Learning Techniques to High-Dimensional Clinical Data to Forecast Postoperative Complications. *PLoS One* **11**, e0155705, doi:10.1371/journal.pone.0155705 (2016).

18 Hobson, C. *et al.* Cost and Mortality Associated With Postoperative Acute Kidney Injury. *Ann Surg* **261**, 1207-1214, doi:10.1097/sla.0000000000000732 (2015).

19 *Clinical Classification Software (CCS)*, <<https://www.hcup-us.ahrq.gov/db/vars/dxmccsn/nisnote.jsp>> (2019).
